# A metabolic–epigenetic switch governs multicellular cardiac repair following succinate dehydrogenase inhibition

**DOI:** 10.1038/s44161-026-00881-9

**Published:** 2026-09-28

**Authors:** Yi Fan, Dakota J. Nuttall, Yi Zhao, Dina Abbasian, Clarissa Shoffler, Christopher Petucci, Wyatt G. Paltzer, Yu-Ling Chang, James Marchant, Diana Presas-Ramos, Kai P. Atlass, Stephen A. Nemr, Jiyoung Bae, Sanjeev S. Ranade, Alexandre R. Colas, Xiao Li, James F. Martin, Ahmed I. Mahmoud

**Affiliations:** 1 Center for Cardiovascular and Muscular Diseases, Sanford Burnham Prebys Medical Discovery Institute, La Jolla, CA USA; 2 Genetics Training Program, University of Wisconsin-Madison, Madison, WI USA; 3 Texas Heart Institute at Baylor College of Medicine, Houston, TX USA; 4 Penn Cardiovascular Institute, Perelman School of Medicine, University of Pennsylvania, Philadelphia, PA USA; 5 Department of Nutritional Sciences, Oklahoma State University, Stillwater, OK USA; 6 Department of Molecular and Human Genetics, Baylor College of Medicine, Houston, TX USA; 7 Department of Integrative Physiology, Baylor College of Medicine, Houston, TX USA

**Keywords:** Disease model, Transcriptomics

## Abstract

Cardiac regeneration is limited in the adult mammalian heart because of restricted cardiomyocyte proliferation and persistent fibrosis. We previously demonstrated that transient pharmacological inhibition of succinate dehydrogenase (SDH) with malonate promotes regeneration after myocardial infarction. Here, we integrate single-nucleus RNA sequencing and ATAC-seq to reveal multicellular transcriptional and epigenetic reprogramming underlying this response. Cell-specific *Sdhb* deletion dissected contributions of SDH inhibition; cardiomyocyte-specific *Sdhb* deletion transiently increased cardiomyocyte proliferation but did not improve post-myocardial infarction function, whereas myofibroblast-specific *Sdhb* deletion suppressed myofibroblast activation and fibrosis and improved cardiac function. SDH inhibition promoted reductive mitochondrial metabolism and remodeled H3K4me3-marked and H3K27me3-marked chromatin states in cardiomyocytes and fibroblasts. Integration of CUT&RUN, chromatin accessibility and transcriptional profiling identified regulatory targets linking metabolic and epigenetic remodeling to regenerative responses. Together, these findings establish SDH as a multicellular regulator of cardiac regeneration and define distinct cell-specific mechanisms contributing to the regenerative effects of transient pharmacological SDH inhibition.

## Abstract (web summary)

Using integrated multiomics and cell-specific genetic models, Fan et al. reveal how succinate dehydrogenase inhibition promotes multicellular cardiac repair after myocardial infarction through coordinated metabolic and epigenetic reprogramming, while defining distinct contributions of cardiomyocytes and myofibroblasts to this response.

## Main

The adult mammalian heart exhibits only a negligible capacity for regeneration following injury^1^. This loss of regenerative potential coincides with the permanent cell-cycle exit of postnatal cardiomyocytes (CMs), which become binucleated or multinucleated^2^. CM maturation is accompanied by a metabolic switch from fetal glycolysis to adult oxidative phosphorylation^3^, epigenetic remodeling of the chromatin landscape that enforces a nonregenerative gene program and a switch from fetal to adult sarcomeric protein isoforms^4,5^.

Metabolic reprogramming has emerged as a promising strategy to enhance regenerative capacity in the adult heart^6–9^. Succinate dehydrogenase (SDH), which links the tricarboxylic acid (TCA) cycle and electron transport chain^10^, has been extensively studied in ischemia–reperfusion injury, where malonate reduces succinate accumulation, mitochondrial reactive oxygen species and tissue injury following reperfusion across multiple mammalian models^11–15^. Moreover, mutations in *SDH* drive profound metabolic reprogramming in multiple cancers characterized by enhanced glycolysis and Warburg-like metabolic states^16^. Therefore, we hypothesized that transient SDH inhibition could metabolically reprogram the adult heart toward a more regenerative state.

Our previous work demonstrated that transient malonate treatment increased CM proliferation and angiogenesis in the adult heart, reduced fibrosis and improved cardiac function following myocardial infarction (MI)^6^. Importantly, delayed treatment beginning 7 days after MI similarly restored cardiac function and reduced fibrosis, supporting a regenerative response beyond acute cardioprotection. SDH inhibition also promoted a metabolic shift toward glycolysis and reactivation of a regenerative-like metabolic program in the adult heart^6^. However, how transient post-MI SDH inhibition, distinct from acute inhibition during ischemia–reperfusion, drives multicellular transcriptional, metabolic and epigenetic programs underlying cardiac regeneration remains unclear. To address this, we integrated single-nucleus RNA sequencing (snRNA-seq), single-nucleus assay for transposase-accessible chromatin with sequencing (snATAC-seq), targeted metabolomics and cell-type-specific *Sdhb* deletion, to dissect responses to SDH inhibition across multiple cardiac cell types following MI.

Our findings reveal that transient SDH inhibition with malonate induces coordinated transcriptional, metabolic and epigenetic reprogramming across multiple cardiac cell types. By contrast, cell-specific *Sdhb* deletion produces distinct effects, including transiently enhanced CM proliferation or reduced fibrotic scarring, without fully recapitulating the regenerative response observed with transient, systemic malonate treatment. Mechanistically, SDH inhibition remodels H3K4me3-associated and H3K27me3-associated chromatin states in CMs and fibroblasts, with integrated CUT&RUN, chromatin accessibility and transcriptional profiling identifying regulatory targets of this response. Together, these results highlight the importance of transient, multicellular metabolic reprogramming in reshaping the cardiac molecular landscape to promote heart regeneration.

## Results

### Global transcriptional and epigenetic analysis of adult mouse hearts following SDH inhibition

**Fig. 1:**
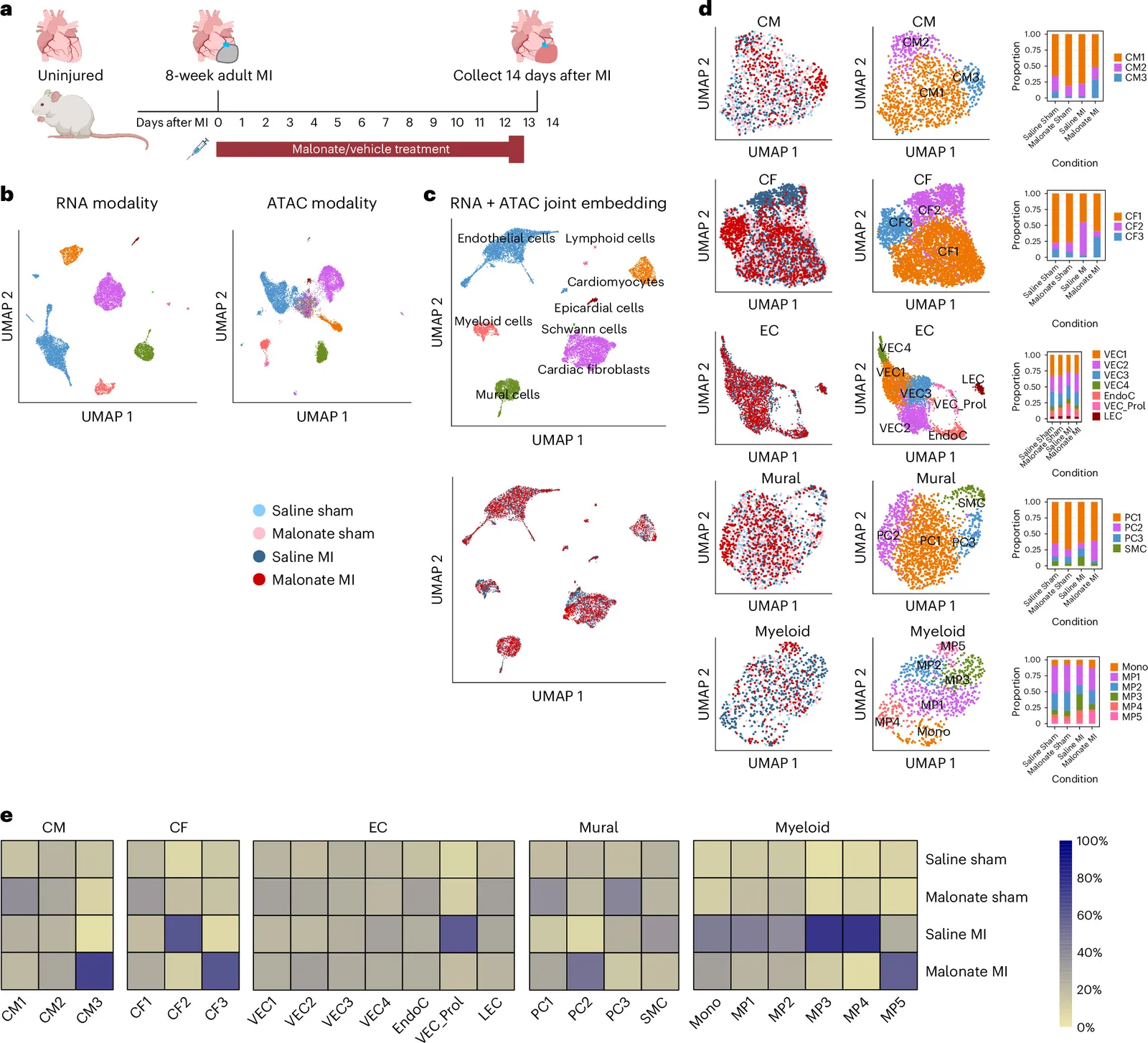
SDH inhibition by malonate after MI induces a distinct transcriptional and epigenetic profile in multiple cardiac cell types. **a**, Schematic of the MI and malonate or vehicle injection strategy in adult mice. **b**, UMAP plots of treatment integrated snRNA (left) and snATAC (right) modalities. **c**, Joint RNA and ATAC embedded UMAP plots clustered by cell type (top) and treatment condition (bottom). **d**, Treatment condition UMAP plots (left), subcluster UMAP plots (middle) and column plots (right) showing the proportion of the subclusters within each treatment condition for the following cardiac cell types that displayed condition-predominant subclustering (in descending order): CMs, CFs, ECs, mural cells and myeloid cells (cell types with population > 800). **e**, Heat maps displaying the proportion of each treatment condition within each subcluster for CMs, CFs, ECs, mural cells and myeloid cells.

**Extended Data Fig. 1:**
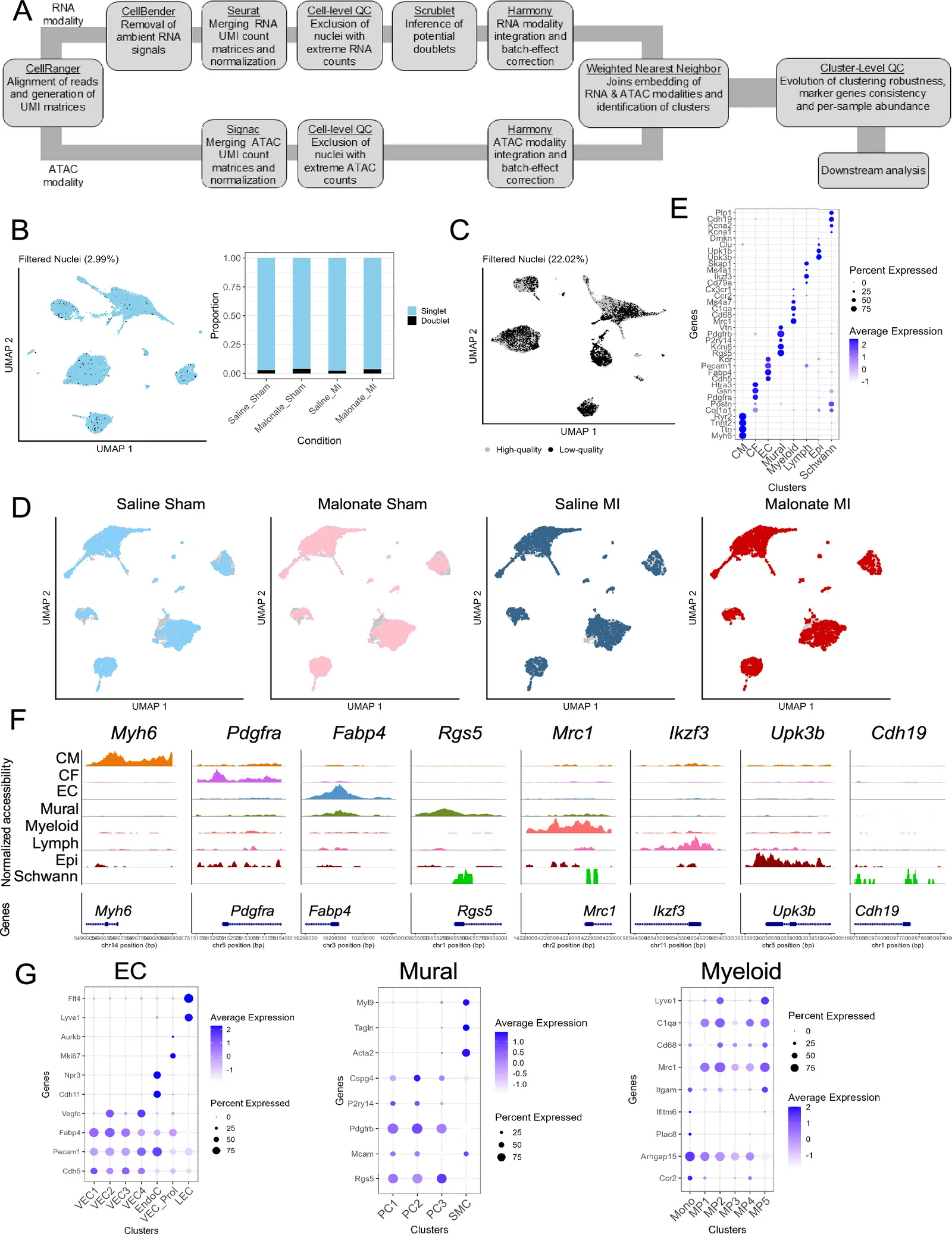
Processing and annotating multiomic single nucleus RNA and ATAC seq data. **a**, The bioinformatical workflow for processing single nucleus RNA and ATAC data for downstream analysis. **b**, RNA UMAP plot and treatment condition proportion plot of annotated doublets identified by Scrublet, and **c**, an RNA UMAP displaying low-quality nuclei clusters with median UMI counts<1000 that were filtered as the final step of quality control. **d**, Treatment condition UMAP plots derived from the final joint embedded RNA & ATAC UMAP in Fig. 1. **e**, Dot plot of key marker genes across all cell types. **f**, Chromatin accessibility coverage plots (bottom) of marker genes used for annotating the following cell types (from left to right): cardiomyocytes (CM)-*Myh6*, cardiac fibroblasts (CF)-*Pdgfra*, endothelial cells (EC)-*Fabp4*, mural cells (Mural)-*Rgs5*, myeloid cells (Myeloid)-*Mrc1*, lymphoid cells (Lymph)-*Izkf3*, epicardial cells (Epi)-*Upk3b*, and Schwann cells (Schwann)-*Cdh19*. **g**, Dot plot of marker genes for defining subpopulations of EC, Mural, and Myeloid populations.

To define the transcriptional and epigenetic landscape of the adult cardiac injury response to SDH inhibition, we used our established pharmacological model of malonate treatment after MI^6^. Adult male mice (8 weeks old) underwent sham or MI surgery followed by 14 daily intraperitoneal (i.p.) injections of saline or dimethyl malonate (Fig. 1a; treatment groups: saline sham, malonate sham, saline MI and malonate MI; four pooled hearts per group). We performed integrated snRNA-seq and snATAC-seq on these hearts and, following quality control, we jointly embedded both modalities to resolve cellular states across conditions (Fig. 1b,c and Extended Data Fig. 1a–d). Cell types were annotated using established markers in combination with differential gene and peak analysis, before subclustering major populations with >800 nuclei (CMs, cardiac fibroblasts (CFs), endothelial cells (ECs), mural cells and myeloid cells) to resolve cellular states^17–23^ (Fig. 1d, Extended Data Fig. 1e–g and Supplementary Table 1).

**Extended Data Fig. 2:**
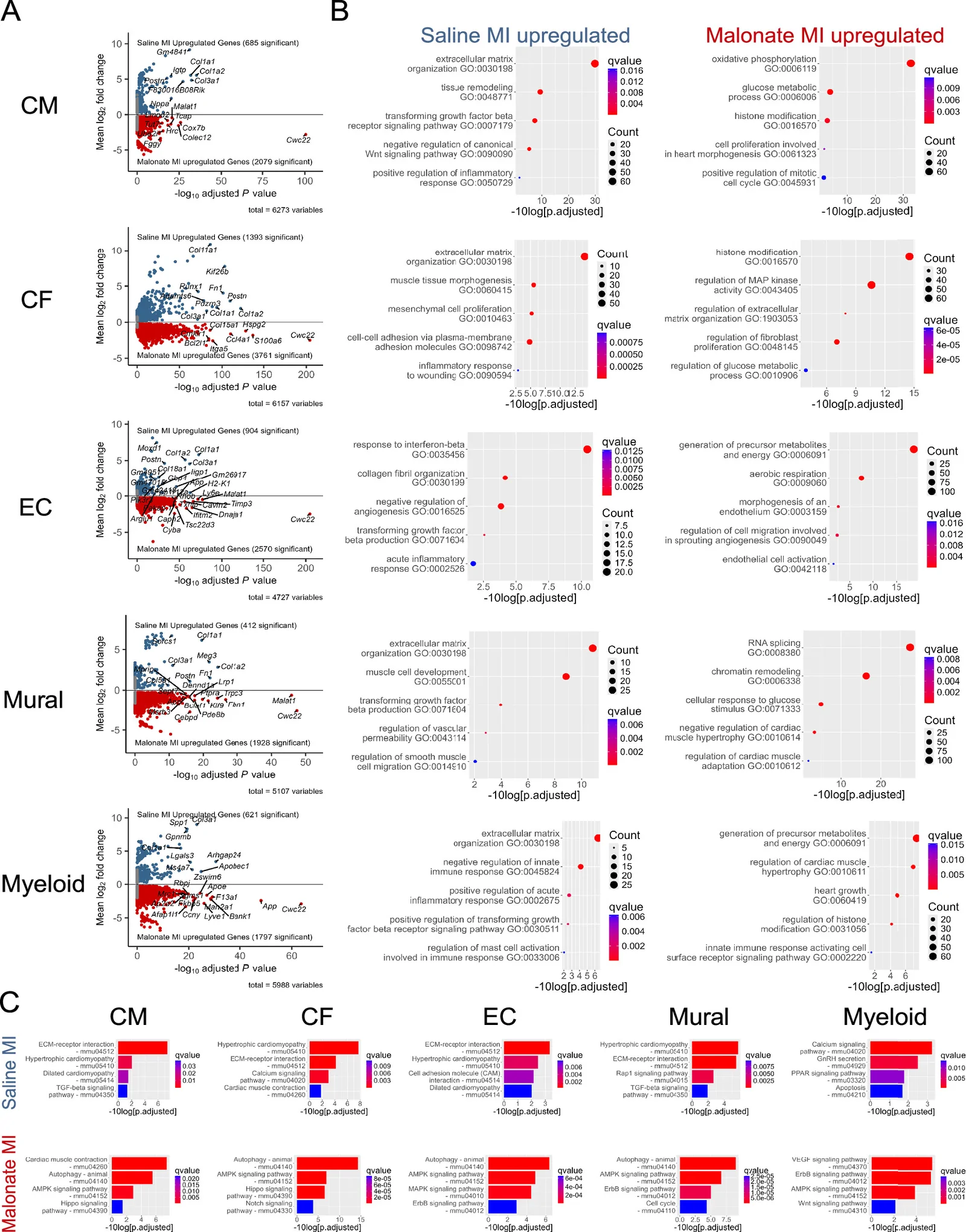
Malonate treatment alters the transcriptional profiles of cardiac injury following MI. **a**, Volcano plots comparing differentially expressed genes between Saline Sham and Malonate Sham as well as Saline MI and Malonate MI treated pseudobulked CMs, CFs, ECs, Mural, and Myeloid populations, displaying an increase in terms associated with scar formation all Saline MI populations and calcium transport in Malonate MI populations. Differentially expressed genes were generated using MAST (likelihood ratio test) differential gene expression analysis with Benjamini-Hochberg adjusted, for multiple comparison, two-sided p values. **b**, One-sided GO plots were then generated from significant differentially expressed genes from the Saline MI vs Malonate MI analysis, all p values are Benjamini-Hochberg adjusted. **c**, One-sided KEGG Pathway enrichment plots generated from the same differentially expressed genes comparing Saline MI to Malonate MI, and p values are Benjamini-Hochberg adjusted. All adjusted p values are significant at P < 0.05.

Each major cell type showed treatment condition-predominant subclusters (>50% of population; Fig. 1e). Saline MI versus malonate MI differential expression and Gene Ontology (GO) revealed enrichment of profibrotic and extracellular matrix programs in saline MI populations, with the strongest gene expression and GO *q* values being in CFs, whereas malonate MI populations were enriched for metabolic, chromatin remodeling and proregenerative programs, particularly in CMs (Extended Data Fig. 2a,b and Supplementary Tables 2 and 3). Kyoto Encyclopedia of Genes and Genomes (KEGG) pathway analysis similarly showed extracellular matrix (ECM) receptor enrichment in the saline MI^24^ and cardioprotective or proglycolytic AMPK signaling in malonate MI populations^25,26^ (Extended Data Fig. 2c and Supplementary Table 4). In sham hearts, saline sham CMs were enriched for mature metabolic pathways, whereas malonate sham CMs and CFs demonstrated programs associated with cardiac development (Supplementary Fig. 1 and Supplementary Tables 2–4). Because CMs and CFs, the primary mediators of regeneration and fibrotic remodeling, respectively, showed the most pronounced treatment-dependent transcriptional and compositional differences, we focused subsequent analyses on these two populations.

### SDH inhibition promotes a proregenerative transcriptional and epigenetic response in CMs

**Fig. 2:**
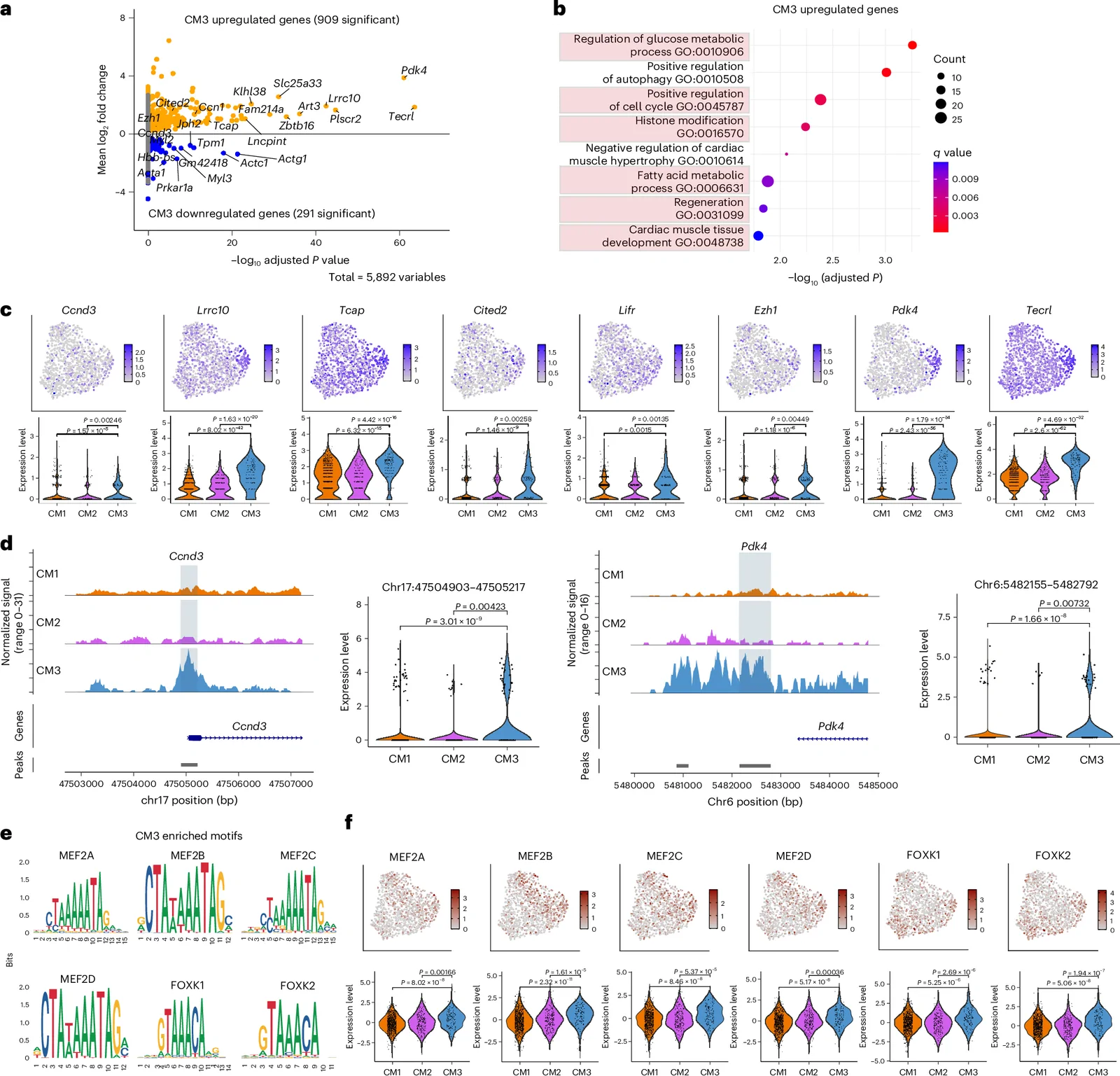
SDH inhibition reprograms CMs into a proregenerative transcriptional and epigenetic state after MI. **a**, A volcano plot showing the significantly upregulated genes and significantly downregulated genes identified using MAST (likelihood ratio test) differential gene expression analysis with Benjamini–Hochberg-adjusted (for multiple comparisons) two-sided *P* values of the malonate MI-predominant CM3 cluster. **b**, A one-sided GO plot displaying upregulated cardiomyogenic and metabolomic functions in CM3. Key terms of interest upregulated in CM3 are highlighted in red; all *P* values are Benjamini–Hochberg-adjusted for multiple comparisons. **c**, Gene expression feature UMAP plots (top) and violin subcluster plots (bottom) of the metabolic, cardiomyogenic and cardioprotective genes *Ccnd3* (*P* = 1.57 × 10^−5^, *P* = 0.00246), *Lrrc10* (*P* = 8.02 × 10^−42^, *P* = 1.63 × 10^−20^), *Tcap* (*P* = 6.32 × 10^−15^, *P* = 4.42 × 10^−16^), *Cited2* (*P* = 1.46 × 10^−9^, *P* = 0.00258), *Lifr* (*P* = 0.0015, *P* = 0.00135), *Ezh1* (*P* = 1.18 × 10^−6^, *P* = 0.00449), *Pdk4* (*P* = 2.43 × 10^−56^, *P* = 1.79 × 10^−34^) and *Tecrl* (*P* = 2.6 × 10^−62^, *P* = 4.69 × 10^−32^). Statistical analysis of genes performed using MAST with two-sided *P* values (*P* values ordered CM3 versus CM1, CM3 versus CM2) that are Benjamini–Hochberg-adjusted for multiple comparisons. **d**, Chromatin accessibility plots showing increased accessibility at the *Ccnd3* (*P* = 3.01 × 10^−9^, *P* = 0.00423) and *Pdk4* (*P* = 1.66 × 10^−8^, *P* = 0.00732) loci in CM3; regions with significantly increased accessibility are highlighted in blue with corresponding violin plots (right) with Benjamini–Hochberg-adjusted *P* values (two-tailed Wilcoxon rank-sum test; *P* values ordered CM3 versus CM1, CM3 versus CM2). **e**,**f**, Motif enrichment plots (**e**) for the cardiomyogenic motifs MEF2A (*P* = 8.02 × 10^−8^, *P* = 0.00166), MEF2B (*P* = 2.32 × 10^−11^, *P* = 1.61 × 10^−5^), MEF2C (*P* = 8.46 × 10^−8^, *P* = 5.37 × 10^−5^), MEF2D (*P* = 5.17 × 10^−8^, *P* = 0.00036), FOXK1 (*P* = 5.25 × 10^−6^, *P* = 2.69 × 10^−6^) and FOXK2 (*P* = 5.06 × 10^−8^, *P* = 1.94 × 10^−7^) observed to have significantly increased accessibility in CM3 and their corresponding motif feature UMAP plots (**f**, top) and violin plots with Benjamini–Hochberg-adjusted *P* values for multiple comparisons (**f**, bottom) (two-tailed Wilcoxon rank-sum test; *P* values ordered CM3 versus, CM1, CM3 versus CM2). All adjusted *P* values were considered significant at *P* < 0.05.

**Extended Data Fig. 3:**
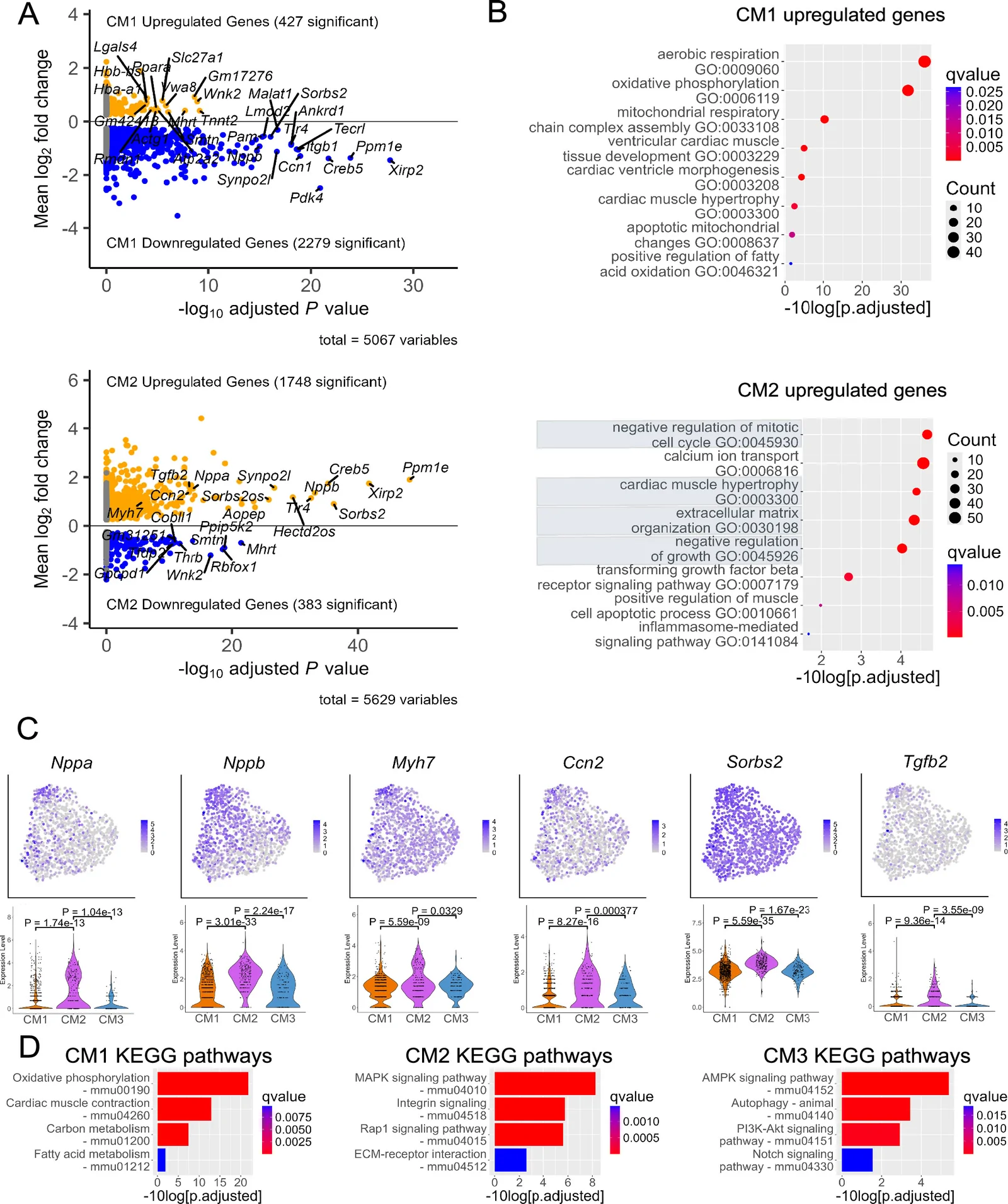
The hypertrophic CM response is distinct from the regenerative response. **a**, Volcano plots showing the significantly upregulated/downregulated genes for CM1 (top) and CM2 (bottom) identified using MAST (likelihood ratio test) differential gene expression analysis with Benjamini-Hochberg adjusted, for multiple comparison, two-sided p values. **b**, One-sided GO plots displaying upregulated terms associated with general cardiac function and respiration in CM1 and CM hypertrophy in CM2. Key terms of interest upregulated in CM2 are highlighted in blue, all p values are Benjamini-Hochberg adjusted. **c**, Gene expression feature UMAP plots (top) and violin subcluster plots (bottom) of hypertrophic and profibrotic genes (*Nppa*: P = 1.74e − 13, P = 1.04e − 13; *Nppb*: P = 3.01e − 33, P = 2.24e − 17; *Myh7*: P = 5.59e − 09, P = 0.0329; *Ccn2*: P = 8.27e − 16, P = 0.000377; *Sorbs2*: P = 5.59e − 35, P = 1.67e − 23; *Tgfb2*: P = 9.36e − 14, P = 3.55e − 09*)*. Statistical analysis of genes performed using MAST with two-sided p values (p values ordered: CM2 vs. CM1, CM2 vs. CM3) that were Benjamini-Hochberg adjusted for multiple comparison. **d**, KEGG pathway enrichment plots generated from two-sided MAST differentially expressed genes for aerobic respiratory pathways in CM1 (left), scar formation signaling pathways in CM2 (middle), and growth signaling pathways in CM3 (right), and p values are Benjamini-Hochberg adjusted. All adjusted p values are significant at P < 0.05.

**Extended Data Fig. 4:**
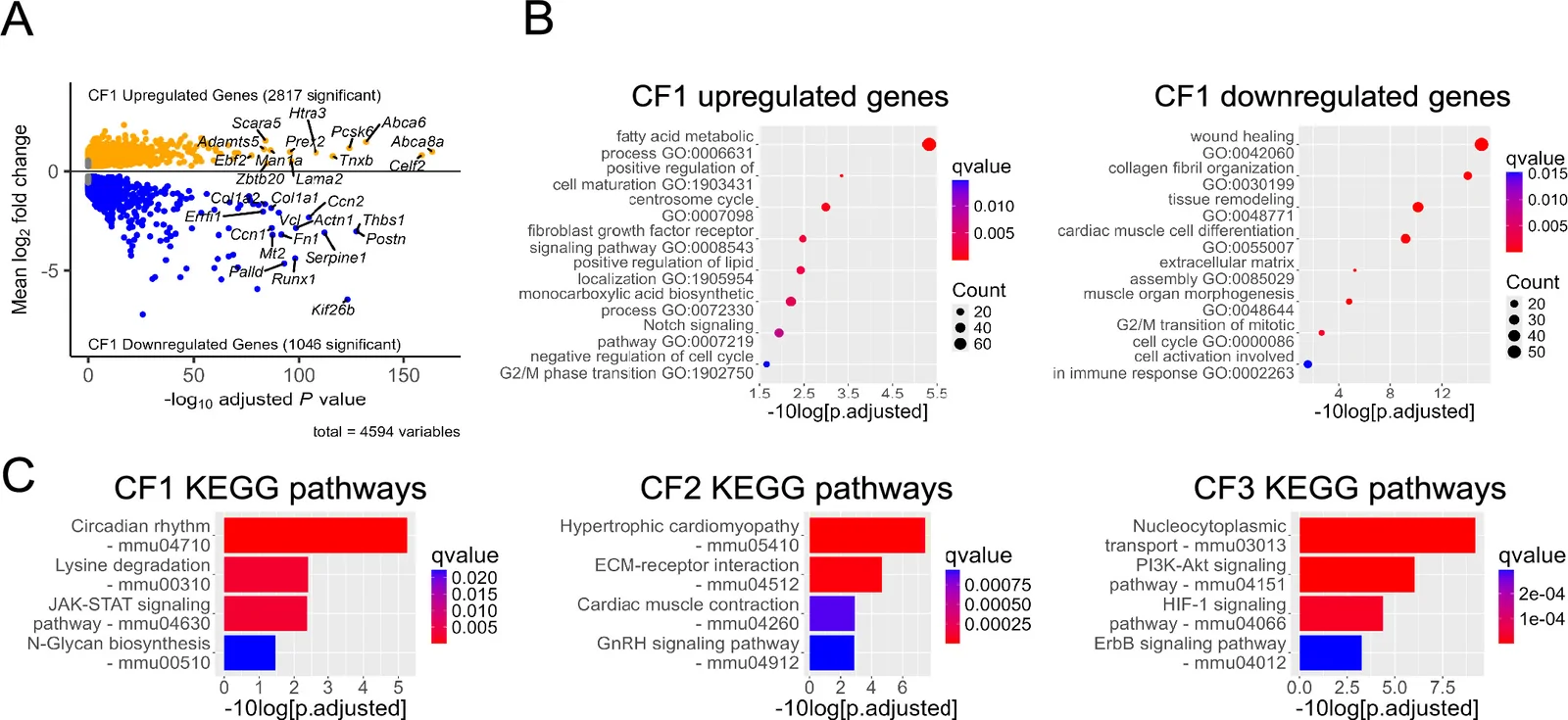
SDH inhibition suppresses cardiomyopathic pathways in cardiac fibroblasts. **a**, A volcano plot showing significantly upregulated/downregulated genes of the CF1 cluster using MAST (likelihood ratio test) differential gene expression analysis with Benjamini-Hochberg adjusted, for multiple comparison, two-sided p values and significant at P < 0.05. **b**, One-sided GO plot displaying upregulated terms associated with fibroblast function and maintenance (left) and downregulated terms associated with myofibroblast activity (right) in CF1, all p values are Benjamini-Hochberg adjusted. **c**, One-sided KEGG pathway enrichment plots generated from MAST differentially expressed genes for pro-fibrotic pathways in CF1 (left), cardiomyopathic pathways in CF2 (middle), and fibroblast senescent pathways in CF3 (right), and p values are Benjamini-Hochberg adjusted. All adjusted p values are significant at P < 0.05.

Subclustering of CM nuclei identified three subclusters, including CM2 (a mixture of saline MI and malonate MI nuclei) and CM3 (predominantly malonate MI) (Fig. 1e), which were then compared using differential expression analysis (Fig. 2a, Extended Data Fig. 4a and Supplementary Table 5). GO analysis of CM3 upregulated genes revealed enrichment of proregenerative processes mirroring the overall Malonate MI CM population, including cell-cycle progression with *Ccnd3* (ref. ^27^) expression, cardiac development with *Lrrc10* (ref. ^28^), *Tcap*^29^ and *Cited2* (ref. ^30^) expression, regeneration with *Lifr*^31^ and *Ezh1* (ref. ^32^) expression and histone modification also with *Ezh1* expression, hallmarks of neonatal-like regenerative CMs (Fig. 2b,c and Supplementary Tables 3 and 5). CM3 also showed enrichment of glucose and fatty acid metabolic processes with expression of *Pdk4* (refs. ^7,9^) and *Tecrl*^33,34^ and activation of PI3K–AKT and AMPK signaling pathways according to KEGG analysis, both of which regulate cellular metabolism and have been implicated in cardioprotective and proregenerative responses^25,26,35^ (Extended Data Fig. 3d and Supplementary Table 4). By contrast, GO analysis revealed CM1 displayed mature metabolic programs, while CM2 was enriched for GO terms injury-associated hypertrophic remodeling with *Nppa*, *Nppb*, *Myh7* and *Ccn2* (ref. ^36^) expression, as well fibrosis-related pathways with *Sorbs2* (ref. ^37^) and *Tgfb2* (ref. ^38^) expression, including ECM receptor interactions and GnRH signaling^39^ identified by KEGG (Extended Data Fig. 3b–d and Supplementary Tables 3–5).

To link these transcriptional patterns with epigenetic remodeling, we assessed chromatin accessibility at CM gene transcription start sites (TSSs). CM3 showed increased accessibility at the cell-cycle gene *Ccnd3* and metabolic regulator *Pdk4* (Fig. 2d and Supplementary Table 6). Differential motif accessibility analysis was then performed on each CM subpopulation and those observed to display significant enrichment can be found in Supplementary Table 7 (Fig. 2e,f and Supplementary Fig. 2a,b). Interestingly, motifs for all four myocyte enhancer factors (MEF2A, MEF2B, MEF2C and MEF2D), which have critical roles in cardiac development and regeneration^40,41^, and motifs for FOXK1 and FOXK2, which promote CM proliferation and regeneration^42^, were also significantly enriched in CM3 (Fig. 2e,f). Differential expression identified few significant transcription factor expression changes (Supplementary Fig. 2c and Supplementary Table 5). Because transcription factor activity can be uncoupled from transcript abundance, motif accessibility may better reflect activity^43^.

Together, these findings support a distinct CM3 state characterized by coordinated transcriptional and epigenetic remodeling toward cardiac growth and regeneration.

### SDH inhibition suppresses fibroblast activation after MI and reprograms CFs toward an antifibrotic state

**Fig. 3:**
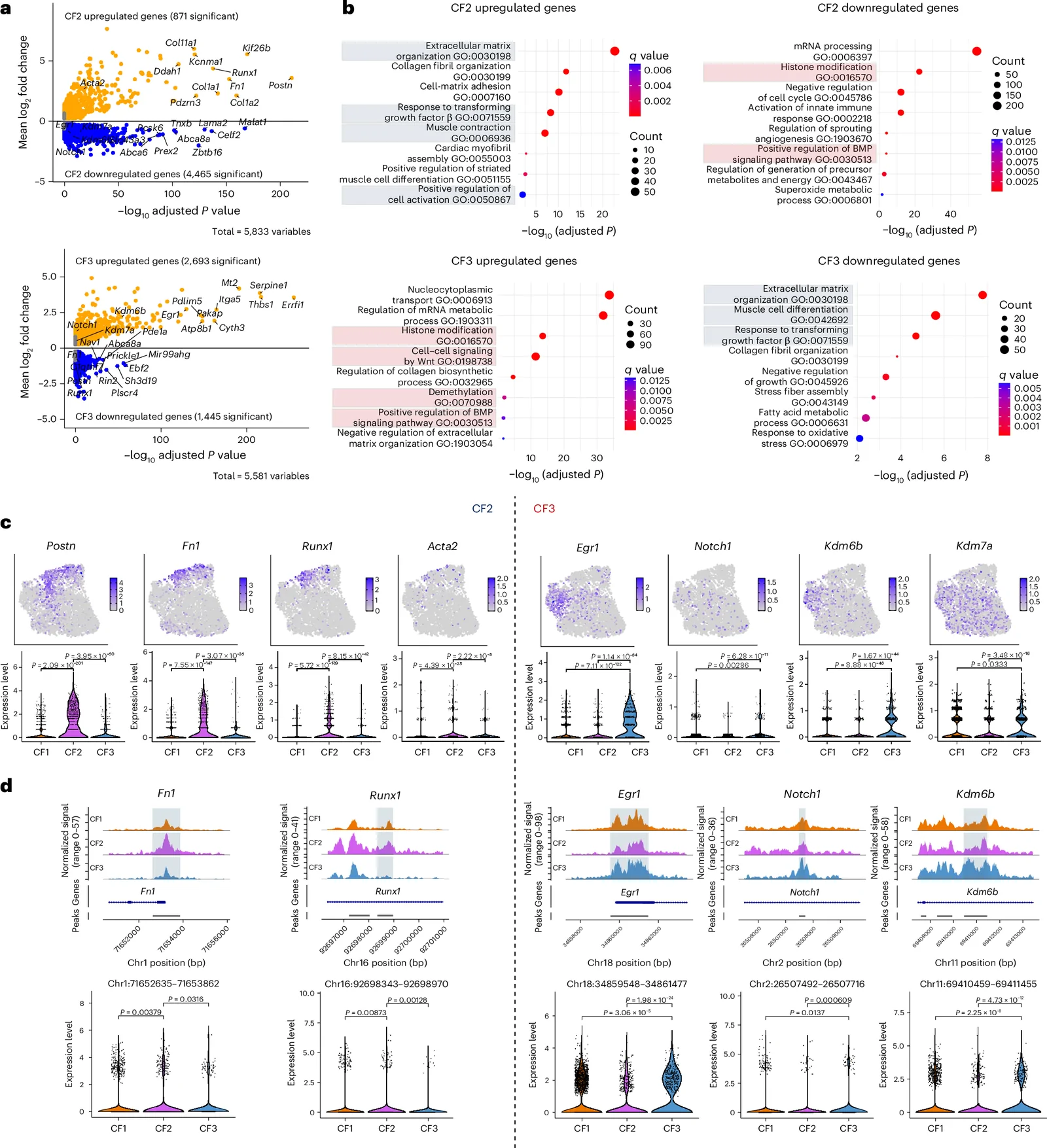
SDH inhibition through malonate inhibits myofibroblast activation after MI. **a**, Volcano plots showing the significantly upregulated and downregulated genes identified using MAST (likelihood ratio test) differential gene expression analysis with Benjamini–Hochberg-adjusted (for multiple comparisons) two-sided *P* values of CF2 (top) and CF3 (bottom). **b**, One-sided GO plots displaying upregulated (left) and downregulated (right) functions of CF2 (top) and CF3 (bottom), which demonstrate that fibrotic functions are observed exclusively in CF2 and absent in CF3. Key terms of interest upregulated in CF2 and CF3 are highlighted in blue and red, respectively; all *P* values are Benjamini–Hochberg-adjusted for multiple comparisons. **c**, Gene expression UMAP plots (top) and violin plots by subcluster (bottom) for profibrotic or myofibroblast genes (*P* values ordered CF2 versus CF1, CF2 versus CF3: *Postn*, *P* = 2.09 × 10^−201^, *P* = 3.95 × 10^−80^; *Fn1*, *P* = 7.55 × 10^−147^, *P* = 3.07 × 10^−58^; *Runx1*, *P* = 5.72 × 10^−139^, *P* = 8.15 × 10^−42^; *Acta2*, *P* = 4.39 × 10^−25^, *P* = 2.22 × 10^−05^) predominantly expressed in CF2 (left of dotted line) and antifibrotic or chromatin remodeling genes (*P* values ordered CF3 versus CF1, CF3 versus CF2: *Egr1*, *P* = 7.11 × 10^−122^, *P* = 1.14 × 10^−84^; *Notch1*, *P* = 0.00286, *P* = 6.28 × 10^−11^; *Kdm6b*, *P* = 8.88 × 10^−48^, *P* = 1.67 × 10^−44^; *Kdm7a*, *P* = 0.0333, *P* = 3.48 × 10^−16^) that are enriched in CF3 (right of dotted line). Statistical analysis of genes performed using MAST framework with two-sided *P* values that are Benjamini–Hochberg-adjusted for multiple comparisons. **d**, Chromatin accessibility plots (top) of genomic regions corresponding to profibrotic genes (*P* values ordered CF2 versus CF1, CF2 versus CF3: *Fn1*, *P* = 0.00379, *P* = 0.0316; *Runx1*, *P* = 0.00873, *P* = 00128; left of dotted line) and antifibrotic or chromatin remodeling genes (*P* values ordered CF3 versus CF1, CF3 versus CF2: *Egr1*, *P* = 3.06 × 10^−5^, *P* = 1.98 × 10^−24^; *Notch1*, *P* = 0.0137, *P* = 0.000609; *Kdm6b*, *P* = 2.25 × 10^−8^, *P* = 4.73 × 10^−12^; right of dotted line); regions with significantly increased accessibility are highlighted in blue, with corresponding violin plots (right) showing Benjamini–Hochberg-adjusted *P* values for multiple comparisons (two-tailed Wilcoxon rank-sum test). All adjusted *P* values were considered significant at *P* < 0.05.

CF subclustering identified two post-MI populations, with CF2 enriched in saline MI and CF3 enriched in malonate MI hearts (Fig. 1f), which were then compared to CF1 using differential expression analysis (Fig. 3a, Extended Data Fig. 4a and Supplementary Table 5). CF2 upregulated genes and GO terms were associated with fibroblast activation and extracellular remodeling, with *Postn*, *Fn1* and *Runx1* expression, as well as myofibroblast differentiation with *Acta2* (ref. ^44^) expression (Fig. 3b,c and Supplementary Tables 3 and 5). By contrast, these fibrosis-associated GO terms were reduced in CF3, which instead showed GO enrichment of reparative WNT/BMP signaling with *Egr1* (ref. ^45^) and *Notch1* (refs. ^46,47^) expression, as well as chromatin remodeling pathways with *Kdm6b* and *Kdm7a* (refs. ^48,49^) expression (Fig. 3b,c and Supplementary Tables 3 and 5). KEGG analysis similarly identified hypertrophic and cell adhesion pathways in CF2 and antifibrotic ErbB^50^ and HIF1 (ref. ^51^) signaling pathways in CF3 (Extended Data Fig. 4c and Supplementary Table 4). Chromatin accessibility mirrored these transcriptional states, with increased accessibility at profibrotic genes in CF2 (*Fn1* and *Runx1*), reparative or histone remodeling genes in CF3 (*Egr1*, *Notch1* and *Kdm6b*) and the reverse pattern at reciprocal loci in each subcluster (Fig. 3d and Supplementary Table 6).

Together, these findings demonstrate that SDH inhibition suppresses the transcriptional and chromatin programs driving fibroblast activation and fibrotic remodeling after MI, shifting fibroblasts toward an antifibrotic, repair-permissive state.

### CM-specific *Sdhb* deletion induces transient CM hyperplasia

**Fig. 4:**
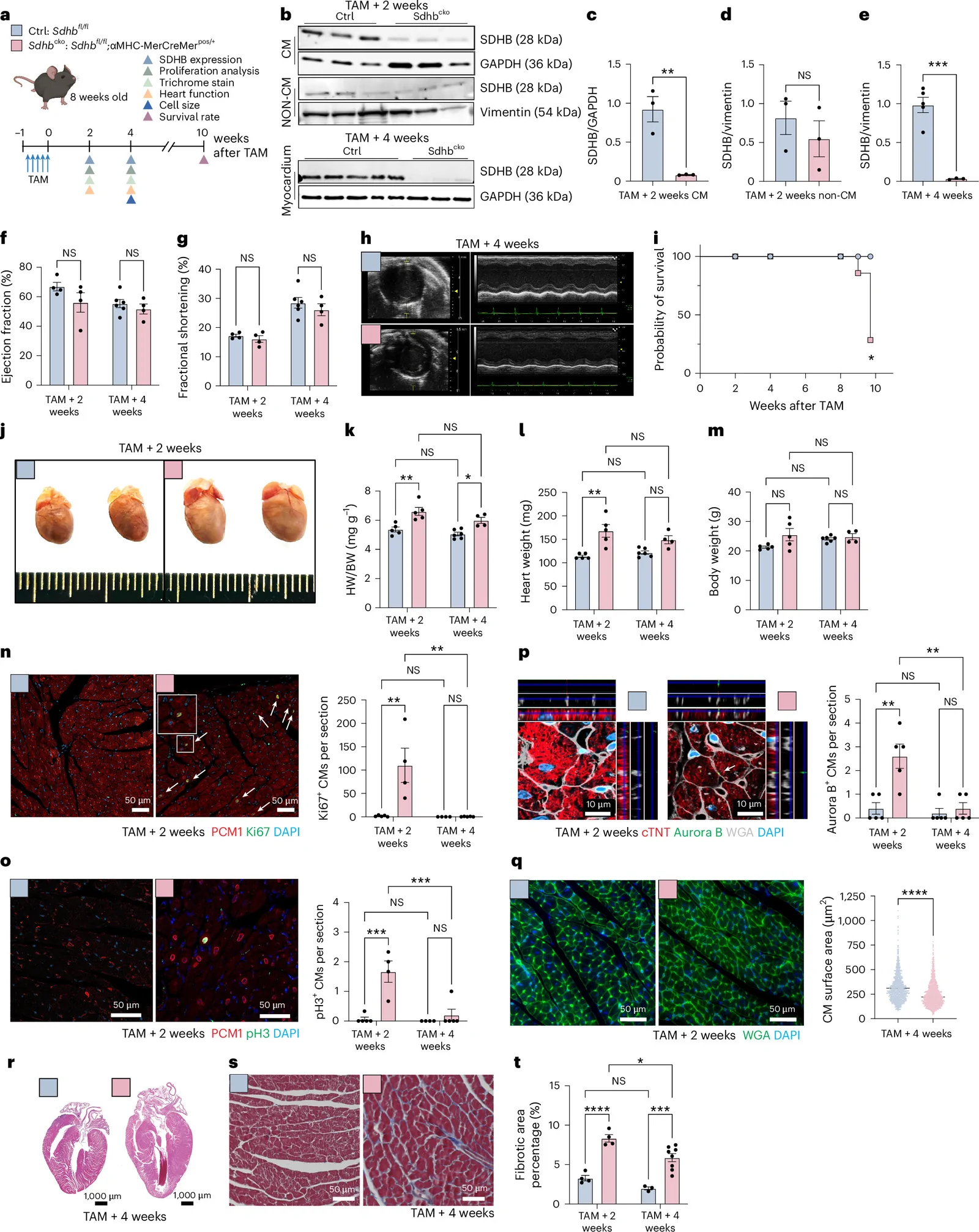
CM-specific *Sdhb* deletion induces transient CM hyperplasia. **a**, Schematic of TAM-inducible CM-specific *Sdhb* deletion and experimental timeline. **b**, Representative western blots showing SDHB protein levels in isolated CMs, non-CMs and whole myocardium from control and *Sdhb*^cko^ hearts at the indicated time points. GAPDH or vimentin was used as a loading control. **c**–**e**, Quantification of SDHB protein levels in isolated CMs at 2 weeks after TAM (*n* = 3 control (Ctrl), *n* = 3 *Sdhb*^cko^ mice; *P* = 0.0067) (**c**), non-CMs at 2 weeks after TAM (*n* = 3 Ctrl, *n* = 3 *Sdhb*^cko^ mice; *P* = 0.4415) (**d**) and myocardium at 4 weeks after TAM (*n* = 5 Ctrl, *n* = 3 *Sdhb*^cko^ mice; *P* = 0.0004) (**e**). Each dot represents an independent biological replicate. An unpaired two-tailed Student’s *t*-test was applied. **f**,**g**, Echocardiographic quantification of ejection fraction (TAM + 2 weeks, Ctrl versus *Sdhb*^cko^, *P* = 0.3140; TAM + 4 weeks, Ctrl versus *Sdhb*^cko^, *P* = 0.9062) (**f**) and fractional shortening (TAM + 2 weeks, Ctrl versus *Sdhb*^cko^, *P* = 0.9653; TAM + 4 weeks, Ctrl versus *Sdhb*^cko^, *P* = 0.7233) (**g**) in control and *Sdhb*^cko^ mice at 2 and 4 weeks after TAM injection. Each dot represents an individual mouse (TAM + 2 weeks, *n* = 4 Ctrl, *n* = 4 *Sdhb*^cko^ mice; TAM + 4 weeks, *n* = 6 Ctrl, *n* = 4 *Sdhb*^cko^ mice). An unpaired two-tailed Student’s *t*-test was applied. **h**, Representative echocardiographic images from control and *Sdhb*^cko^ mice at 4 weeks after TAM. **i**, Kaplan–Meier survival curve of control (*n* = 4) and *Sdhb*^cko^ (*n* = 4) mice after TAM induction (*P* = 0.0387). Survival was analyzed using a log-rank test. **j**, Representative gross heart images from control and *Sdhb*^cko^ mice at 2 weeks after TAM. **k**,**m**, Quantification of HW/BW ratio (TAM + 2 weeks, Ctrl versus *Sdhb*^cko^, *P* = 0.0018; TAM + 4 weeks, Ctrl versus *Sdhb*^cko^, *P* = 0.0195; Ctrl, TAM + 2 weeks versus TAM + 4 weeks, *P* = 0.6508; *Sdhb*^cko^, TAM + 2 weeks versus TAM + 4 weeks, *P* = 0.1983) (**k**), HW (TAM + 2 weeks, Ctrl versus *Sdhb*^cko^, *P* = 0.0011; TAM + 4 weeks, Ctrl versus *Sdhb*^cko^, *P* = 0.1224; Ctrl, TAM + 2 weeks versus TAM + 4 weeks, *P* = 0.8908; *Sdhb*^cko^, TAM + 2 weeks versus TAM + 4 weeks, *P* = 0.4167) (**l**) and BW (TAM + 2 weeks, Ctrl versus *Sdhb*^cko^, *P* = 0.0876; TAM + 4 weeks, Ctrl versus *Sdhb*^cko^, *P* = 0.9686; Ctrl, TAM + 2 weeks versus TAM + 4 weeks, *P* = 0.3215; *Sdhb*^cko^, TAM + 2 weeks versus TAM + 4 weeks, *P* = 0.9803) (**m**) at 2 and 4 weeks after TAM (TAM + 2 weeks, *n* = 5 Ctrl, *n* = 5 *Sdhb*^cko^ mice; TAM + 4 weeks, *n* = 6 Ctrl, *n* = 4 *Sdhb*^cko^ mice). Each dot represents an individual mouse. A two-way ANOVA with Tukey’s post hoc test was applied. **n**, Representative immunofluorescence images and quantification of Ki67^+^ CMs in control and *Sdhb*^cko^ hearts at 2 and 4 weeks after TAM (TAM + 2 weeks, *n* = 5 Ctrl, *n* = 4 *Sdhb*^cko^ mice; TAM + 4 weeks, *n* = 4 Ctrl, *n* = 5 *Sdhb*^cko^ mice; TAM + 2 weeks, Ctrl versus *Sdhb*^cko^, *P* = 0.0015; TAM + 4 weeks, Ctrl versus *Sdhb*^cko^, *P* = 0.9999; Ctrl, TAM + 2 weeks versus TAM + 4 weeks, *P* = 0.9998; *Sdhb*^cko^, TAM + 2 weeks versus TAM + 4 weeks, *P* = 0.0014). CM nuclei were identified by PCM1 staining. Arrows indicate Ki67^+^PCM1^+^ CMs. Each dot represents one heart section from an individual mouse. A two-way ANOVA with Tukey’s post hoc test was applied. **o**, Representative immunofluorescence images and quantification of pH3^+^ CMs in control and *Sdhb*^cko^ hearts at 2 and 4 weeks after TAM (TAM + 2 weeks, *n* = 5 Ctrl, *n* = 4 *Sdhb*^cko^ mice; TAM + 4 weeks, *n* = 4 Ctrl, *n* = 5 *Sdhb*^cko^ mice; TAM + 2 weeks, Ctrl versus *Sdhb*^cko^, *P* = 0.0003; TAM + 4 weeks, Ctrl versus *Sdhb*^cko^, *P* = 0.8901; Ctrl, TAM + 2 weeks versus TAM + 4 weeks, *P* = 0.9950; *Sdhb*^cko^, TAM + 2 weeks versus TAM + 4 weeks, *P* = 0.0006). Each dot represents the average of two sections from an individual mouse. A two-way ANOVA with Tukey’s post hoc test was applied. **p**, Representative immunofluorescence images and quantification of Aurora B^+^ CMs in control and *Sdhb*^cko^ hearts at 2 and 4 weeks after TAM (TAM + 2 weeks, *n* = 5 Ctrl, *n* = 5 *Sdhb*^cko^ mice; TAM + 4 weeks, *n* = 5 Ctrl, *n* = 5 *Sdhb*^cko^ mice; TAM + 2 weeks, Ctrl versus *Sdhb*^cko^, *P* = 0.0010; TAM + 4 weeks, Ctrl versus *Sdhb*^cko^, *P* = 0.9713; Ctrl, TAM + 2 weeks versus TAM + 4 weeks, *P* = 0.9713; *Sdhb*^cko^, TAM + 2 weeks versus TAM + 4 weeks, *P* = 0.0010). CMs were identified by cTnT staining and cell borders were visualized by WGA. Each dot represents one heart section from an individual mouse. A two-way ANOVA with Tukey’s post hoc test was applied. **q**, Representative WGA staining and quantification of CM cross-sectional area in hearts from control (*n* = 3 mice, 971 CMs in total included) and *Sdhb*^cko^ (*n* = 4 mice, 1,232 CMs in total included) at 4 weeks after TAM injection (*P* = 0.0001). An unpaired two-tailed Student’s *t*-test was applied. **r**, Representative H&E-stained heart sections from control and *Sdhb*^cko^ mice at 4 weeks after TAM (one heart per group). **s**,**t**, Representative Masson’s trichrome staining (**s**) and quantification of fibrotic area (**t**) in control and *Sdhb*^cko^ hearts at 2 and 4 weeks after TAM (TAM + 2 weeks, *n* = 4 Ctrl, *n* = 4 *Sdhb*^cko^ mice; TAM + 4 weeks, *n* = 3 Ctrl, *n* = 8 *Sdhb*^cko^ mice; TAM + 2 weeks, Ctrl versus *Sdhb*^cko^, *P* = 0.0001; TAM + 4 weeks, Ctrl versus *Sdhb*^cko^, *P* = 0.0007; Ctrl, TAM + 2 weeks versus TAM + 4 weeks, *P* = 0.4583; *Sdhb*^cko^, TAM + 2 weeks versus TAM + 4 weeks, *P* = 0.0144). Each dot represents the average of five images from an individual mouse. A two-way ANOVA with Tukey’s post hoc test was applied. Data are presented as the mean ± s.e.m. NS, not significant; *P < 0.05, **P < 0.01, ***P < 0.001 and ****P < 0.0001. Scale bars are indicated. Source data

Our single-cell and histological analyses demonstrated that transient SDH inhibition through systemic malonate treatment significantly enhances CM proliferation^6^. However, whether this effect is mediated specifically through CM-intrinsic SDH inhibition remains unclear. To address this, we generated a CM-specific *Sdhb*-knockout mouse. *Sdhb* encodes a core subunit of the SDH complex and its deletion enables CM-specific inhibition of SDH activity. We used tamoxifen (TAM)-inducible αMHC-MerCreMer^pos/+^;*Sdhb*^*fl/fl*^ mice, hereafter referred to as *Sdhb*^cko^ mice (Fig. 4a). Following five consecutive daily TAM injections in adult mice, we performed serial phenotypic analyses at 2 and 4 weeks after induction.

At 2 weeks after induction, SDHB protein was efficiently depleted in isolated CMs from *Sdhb*^cko^ hearts, whereas SDHB expression was preserved in non-CMs (Fig. 4b–d). Consistent with the cardiac-restricted nature of this model, SDHB protein levels were unchanged in liver tissue (Supplementary Fig. 3). Efficient SDHB depletion was also maintained in the myocardium at 4 weeks after induction (Fig. 4b,e). Echocardiographic analysis showed that cardiac systolic function, including ejection fraction and fractional shortening, was preserved at both 2 and 4 weeks after induction (Fig. 4f–h). Despite this initially preserved cardiac function, *Sdhb*^cko^ mice exhibited a rapid decline in survival by 11 weeks after induction (Fig. 4i). Morphometric analysis revealed that *Sdhb*^cko^ mice had significantly increased heart weight and an increased heart weight/body weight ratio at 2 weeks after induction, without a change in body weight. However, this increase in heart weight and the heart weight/body weight ratio was not further increased at 4 weeks after induction (Fig. 4j–m).

To determine the basis for the early increase in heart weight following *Sdhb* deletion, we examined CM proliferation, size and fibrosis at 2 and 4 weeks after induction. CM proliferation was assessed by costaining of the CM nuclear marker PCM1 with the cell-cycle reentry marker Ki67 and the mitotic marker phospho-histone H3 (pH3). Cytokinesis was evaluated by Aurora B staining in combination with cardiac troponin T (cTNT) and whole-genome amplification (WGA). At 2 weeks after induction, *Sdhb*^cko^ hearts exhibited a striking increase in proliferating CMs compared to *Sdhb*^*fl/fl*^ controls, as evidenced by a marked increase in Ki67^+^ CMs (approximately 100-fold), as well as increased percentages of pH3^+^ and Aurora B^+^ CMs (each approximately threefold; Fig. 4n–p). Interestingly, these changes were transient and returned to baseline levels by 4 weeks after induction (Fig. 4n–p). In addition, CM size was significantly reduced in *Sdhb*^cko^ hearts at 4 weeks after induction (Fig. 4q). Together, these findings indicate that CM-specific *Sdhb* deletion induces a transient burst of proliferative activity. Overall cardiac histological architecture appeared largely preserved at 4 weeks after induction, as shown by hematoxylin and eosin (H&E) staining (Fig. 4r); however, Masson’s trichrome staining revealed a mild increase in fibrosis at 2 weeks after induction, which was partially reduced by 4 weeks (Fig. 4s,t).

### MI does not extend the transient hyperplastic response induced by CM-specific *Sdhb* deletion

**Extended Data Fig. 5:**
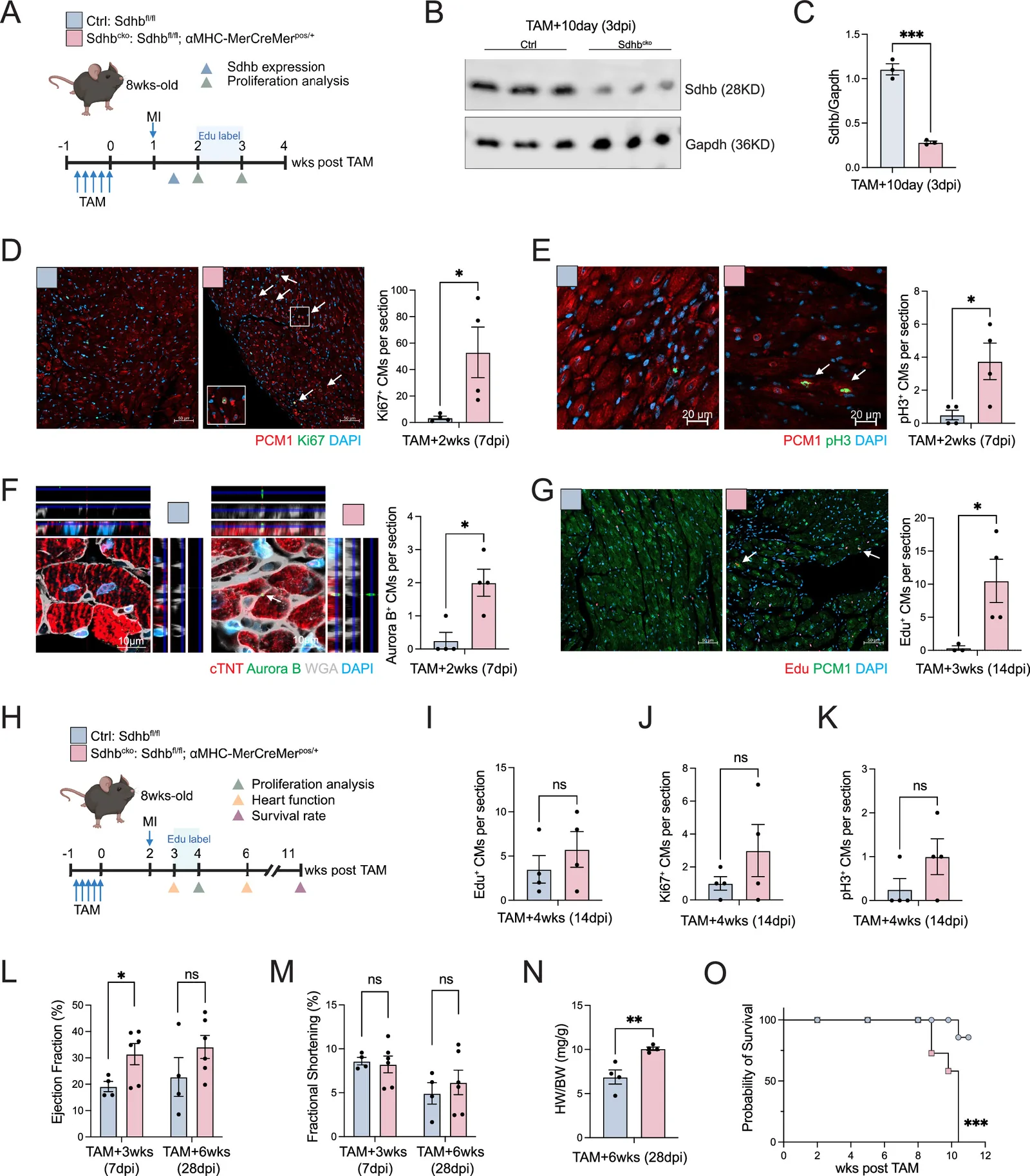
MI does not enhance or prolong Sdhb deletion-induced transient cardiomyocyte hyperplasia. **a**, Schematic of the experimental strategy to determine whether MI further enhances cardiomyocyte proliferation during the transient proliferative window induced by cardiomyocyte-specific *Sdhb* deletion. **b, c**, Representative western blot (**b**) and quantification (**c**) of SDHB protein levels in myocardium from control and *Sdhb*^cko^ hearts at 10 days post-TAM, corresponding to 3 days post-MI (n = 3 mice in Ctrl, n = 3 mice in *Sdhb*^cko^; P = 0.0002). GAPDH was used as a loading control. Each dot represents an independent biological replicate. Unpaired two-tailed Student’s t-test was applied. **d-f**, Representative immunofluorescence images and quantification of Ki67+ cardiomyocytes (P = 0.0417) (**d**), pH3+ cardiomyocytes (P = 0.0297) (**e**) and Aurora B+ cardiomyocytes (P = 0.0106) (**f**) at 2 weeks post-TAM, corresponding to 7 days post-MI (n = 4 mice in Ctrl, n = 4 mice in *Sdhb*^cko^). Cardiomyocytes were identified by PCM1 or cTnT staining, and cell borders were visualized by WGA where indicated. Arrows indicate marker-positive cardiomyocytes. Each dot represents one section collected 250 μm from the ligation level in an individual mouse. Unpaired two-tailed Student’s t-test was applied. **g**, Representative immunofluorescence images and quantification of EdU+ cardiomyocytes at 3 weeks post-TAM, corresponding to 14 days post-MI (n = 3 mice in Ctrl, n = 4 mice in *Sdhb*^cko^; P = 0.0474). EdU was administered from 2 to 3 weeks post-TAM. Each dot represents one section collected 250 μm from the ligation level in an individual mouse. Unpaired two-tailed Student’s t-test was applied. **h**, Schematic of the experimental strategy to determine whether MI prolongs the transient proliferative response induced by cardiomyocyte-specific *Sdhb* deletion. **i-k**, Quantification of EdU+ cardiomyocytes (P = 0.4107) (**i**), Ki67+ cardiomyocytes (P = 0.2666) (**j**) and pH3+ cardiomyocytes (P = 0.1682) (**k**) at 4 weeks post-TAM, corresponding to 14 days post-MI (n = 4 mice in Ctrl, n = 4 mice in *Sdhb*^cko^). Each dot represents one section collected 250 μm from the ligation level in an individual mouse. Unpaired two-tailed Student’s t-test was applied. **l, m**, Echocardiographic quantification of ejection fraction (TAM+3wks, Ctrl vs. *Sdhb*^cko^: P = 0.0473; TAM+6wks, Ctrl vs. *Sdhb*^cko^: P = 0.1918) (**l**) and fractional shortening (TAM+3wks, Ctrl vs. *Sdhb*^cko^: P = 0.7701; TAM+6wks, Ctrl vs. *Sdhb*^cko^: P = 0.5485) (**m**) at 3 and 6 weeks post-TAM, corresponding to 7 and 28 days post-MI, respectively (TAM+3wks: n = 4 mice in Ctrl, n = 6 mice in *Sdhb*^cko^; TAM+6wks: n = 4 mice in Ctrl, n = 6 mice in *Sdhb*^cko^). Each dot represents an individual mouse. Unpaired two-tailed Student’s t-test was applied. **n**, Quantification of heart weight-to-body weight ratio at 6 weeks post-TAM, corresponding to 28 days post-MI. Each dot represents an individual mouse (n = 4 mice in Ctrl, n = 4 mice in *Sdhb*^cko^; P = 0.0080). Unpaired two-tailed Student’s t-test was applied. **o**, Kaplan–Meier survival curve of control (n = 4 mice) and *Sdhb*^cko^(n = 6 mice) mice following TAM induction and MI. Survival was analyzed by log-rank test (P = 0.0009). Data are presented as mean ± s.e.m. ns, not significant; *P < 0.05, **P < 0.01, ***P < 0.001, ****P < 0.0001. Scale bars are indicated in the images. Source data

To determine whether the proliferative response induced by CM-specific *Sdhb* deletion is sufficient to improve cardiac repair after MI, we first asked whether injury could further enhance or prolong this transient response. To this end, adult mice underwent MI surgery at 1 week after induction and CM proliferation was assessed at 2 weeks after induction, corresponding to 7 days after MI. In parallel, EdU was administered from 2 to 3 weeks after induction (7–14 days after MI) to label proliferating CMs during this period (Extended Data Fig. 5a). Efficient deletion of *Sdhb* was confirmed at 10 days after induction (3 days after MI) (Extended Data Fig. 5b,c). At 2 weeks after induction, the percentage of Ki67^+^, pH3^+^, Aurora B^+^ and cumulative EdU^+^ CMs were all increased in *Sdhb*^cko^ hearts compared to *Sdhb*^*fl/fl*^ controls; however, none of these levels exceeded the proliferative response observed in uninjured *Sdhb*^cko^ hearts at the same time point, indicating that MI does not further augment *Sdhb*^cko^-driven proliferation (Extended Data Fig. 5d–g).

We next tested whether MI could prolong the transient proliferative response in *Sdhb*^cko^ hearts. For this, MI surgery was performed at 2 weeks after induction, EdU was administered from 3 to 4 weeks after induction (7–14 days after MI) and CM proliferation was evaluated at 4 weeks after induction (14 days after MI) (Extended Data Fig. 5h). Under these conditions, the percentage of EdU^+^, Ki67^+^ and pH3^+^ CMs were not increased compared to *Sdhb*^*fl/fl*^ controls (Extended Data Fig. 5i–k). Cardiac function was monitored at 3 and 6 weeks after induction, corresponding to 7 and 28 days after MI, respectively and no improvement was observed in *Sdhb*^cko^ mice (Extended Data Fig. 5l,m). The heart weight/body weight ratio remained significantly increased at 6 weeks after induction, consistent with the early hyperplastic phenotype (Extended Data Fig. 5n). Moreover, survival analysis showed that all *Sdhb*^cko^ mice died by approximately 11 weeks after induction regardless of MI, indicating that sustained CM-specific SDH inhibition is ultimately detrimental despite the early proliferative response (Extended Data Fig. 5o). Together, these data demonstrate that, while CM-specific *Sdhb* deletion induces a robust but transient proliferative response at baseline and after MI, sustained SDH deletion in CMs results in increased mortality, underscoring the importance of transient rather than sustained SDH inhibition for a beneficial outcome.

### CM-specific *Sdhb* deletion induces CM proliferation and reduces scar area after MI but does not restore cardiac function

**Fig. 5:**
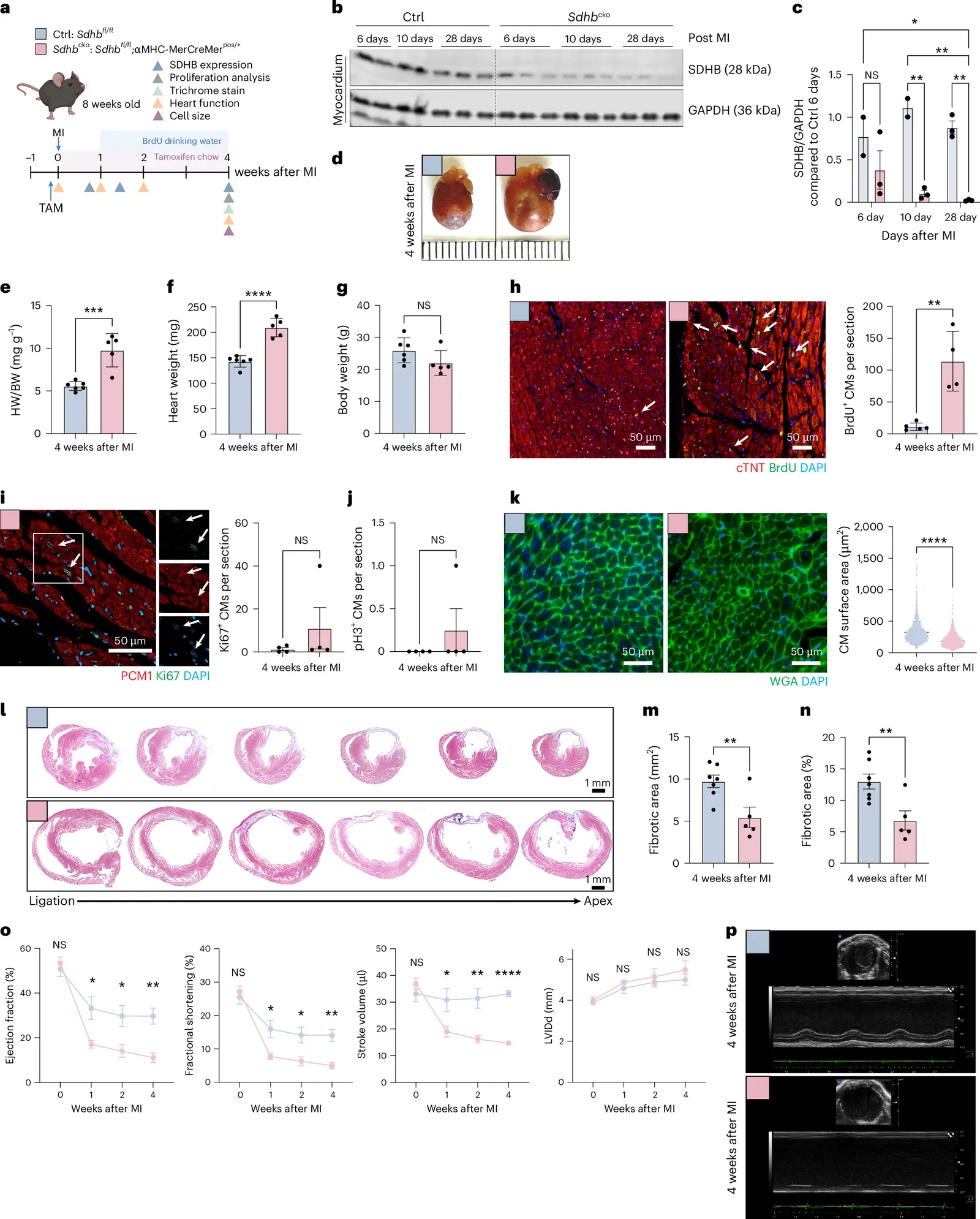
Sustained CM-specific *Sdhb* deletion promotes CM proliferation and reduces scar formation after MI but does not restore cardiac function. **a**, Schematic of the experimental strategy for sustained CM-specific *Sdhb* deletion after MI. **b**,**c**, Representative western blot (**b**) and quantification (**c**) of SDHB protein levels in myocardium from control and *Sdhb*^cko^ hearts at 6, 10 and 28 days after MI (6 days, *n* = 2 Ctrl, *n* = 3 *Sdhb*^cko^ mice; 10 days, *n* = 2 Ctrl, *n* = 3 *Sdhb*^cko^ mice; 28 days, *n* = 3 Ctrl, *n* = 3 *Sdhb*^cko^ mice; 6 days, Ctrl versus *Sdhb*^cko^, *P* = 0.4156; 10 days, Ctrl versus *Sdhb*^cko^, *P* = 0.0047; 28 days, Ctrl versus *Sdhb*^cko^, *P* = 0.0068; Ctrl 6 days versus *Sdhb*^cko^ 28 days, *P* = 0.0317; Ctrl 10 days versus *Sdhb*^cko^ 28 days, *P* = 0.0027). GAPDH was used as a loading control. Each dot represents an independent biological replicate. A two-way ANOVA with Tukey’s post hoc test was applied. **d**, Representative gross heart images from control and *Sdhb*^cko^ mice at 4 weeks after MI. **e**–**g**, Quantification of the heart weight/body weight (HW/BW) ratio (*P* = 0.0006) (**e**), heart weight (HW) (*P* = 0.0001) (**f**) and body weight (BW) (*P* = 0.1272) (**g**) at 4 weeks after MI (*n* = 6 mice in Ctrl, *n* = 5 mice in *Sdhb*^cko^). Each dot represents an individual mouse. An unpaired two-tailed Student’s *t*-test was applied. **h**, Representative immunofluorescence images and quantification of BrdU^+^ CMs at 4 weeks after MI. CMs were identified by cTnT staining (*n* = 5 Ctrl, *n* = 4 *Sdhb*^cko^ mice; *P* = 0.0017). Arrows indicate BrdU^+^ cTnT^+^ CMs. Each dot represents one heart section from an individual mouse. An unpaired two-tailed Student’s *t*-test was applied. **i**, Representative immunofluorescence images and quantification of Ki67^+^ CMs at 4 weeks after MI (*n* = 4 Ctrl, *n* = 4 *Sdhb*^cko^ mice; *P* = 0.3536). CM nuclei were identified by PCM1 staining. Arrows indicate Ki67^+^PCM1^+^ CMs. Each dot represents one heart section from an individual mouse. An unpaired two-tailed Student’s *t*-test was applied. **j**, Quantification of pH3^+^ CMs at 4 weeks after MI (*n* = 4 Ctrl, *n* = 4 *Sdhb*^cko^ mice; *P* = 0.3559). Each dot represents one heart section from an individual mouse. An unpaired two-tailed Student’s *t*-test was applied. **k**, Representative WGA staining and quantification of CM cross-sectional area in control (*n* = 5 mice, 1,614 CMs in total included) and *Sdhb*^cko^ (*n* = 4 mice, 1,383 CMs in total included) hearts at 4 weeks after MI (*P* = 0.0001). An unpaired two-tailed Student’s *t*-test was applied. **l**, Representative Masson’s trichrome-stained serial heart sections from control and *Sdhb*^cko^ mice at 4 weeks after MI, arranged from the ligation site to the apex. **m**,**n**, Quantification of fibrotic area (*P* = 0.0098) (**m**) and fibrotic area percentage (*P* = 0.0087) (**n**) at 4 weeks after MI (*n* = 7 Ctrl, *n* = 5 *Sdhb*^cko^ mice). Each dot represents the average of six sections from an individual mouse. An unpaired two-tailed Student’s *t*-test was applied. **o**, Serial echocardiographic assessment of cardiac function before MI and at 1, 2 and 4 weeks after MI, including ejection fraction, fractional shortening, stroke volume and left ventricular internal diameter in diastole (LVIDd). Control, *n* = 5 mice for pre-MI, *n* = 6 mice for 1, 2 and 4 weeks after MI; *Sdhb*^cko^, *n* = 5 mice per time point (left to right, 0, 1, 2 and 4 weeks after MI, *P* = 0.5572, 0.0217, 0.0235 and 0.0021; *P* = 0.5458, 0.0218, 0.0218 and 0.0021; *P* = 0.3437, 0.0462, 0.0055 and 0.0001; *P* = 0.5819, 0.3442, 0.5781 and 0.3674). An unpaired two-tailed Student’s *t*-test was applied. **p**, Representative echocardiographic images from control and *Sdhb*^cko^ mice at 4 weeks after MI. Data are presented as the mean ± s.e.m. *P < 0.05, **P < 0.01, ***P < 0.001 and ****P < 0.0001. Scale bars are indicated. Source data

Considering the narrow proliferative window following *Sdhb* deletion, we next asked whether CM-specific *Sdhb* deletion after MI could promote cardiac repair. To this end, adult mice received a high-dose TAM injection 1 day before MI surgery, followed by continuous TAM chow administration until 4 weeks after MI to maintain *Sdhb* deletion (Fig. 5a). BrdU was provided in the drinking water from 1 to 4 weeks after MI to label CMs undergoing cumulative proliferation during the repair phase. SDHB protein levels were examined at 6 days, 10 days and 4 weeks after MI. Efficient SDHB depletion was detected by 10 days after MI and was maintained at 4 weeks after MI in *Sdhb*^cko^ hearts (Fig. 5b,c). Gross morphological examination at 4 weeks after MI revealed that *Sdhb*^cko^ hearts were enlarged but exhibited smaller infarct scars compared to *Sdhb*^*fl/fl*^ controls. Consistent with this, heart weight and the heart weight/body weight ratio were significantly increased in *Sdhb*^cko^ mice, whereas body weight was unchanged (Fig. 5d–g).

Analysis of BrdU incorporation revealed a marked increase in BrdU^+^ CMs in *Sdhb*^cko^ hearts at 4 weeks after MI, indicating cumulative CM proliferation between 1 and 4 weeks after injury (Fig. 5h). In contrast, the percentages of Ki67^+^ and pH3^+^ CMs were not increased at 4 weeks after MI, suggesting that the active proliferative response had largely subsided by this later stage, consistent with the transient proliferative phenotype observed after *Sdhb* deletion in uninjured hearts (Fig. 5i,j). CM size was also reduced in *Sdhb*^cko^ hearts at 4 weeks after MI, consistent with hyperplastic rather than hypertrophic growth, indicating an increase in cell number rather than cell size (Fig. 5k). Masson’s trichrome staining showed a significant reduction in scar area in *Sdhb*^cko^ hearts at 4 weeks after MI, indicating improved structural repair after injury (Fig. 5l–n). Despite reduced scar formation and increased cumulative CM proliferation, serial echocardiographic analysis at 1, 2 and 4 weeks after MI revealed progressive deterioration of systolic function in *Sdhb*^cko^ mice, as shown by reduced ejection fraction, fractional shortening and stroke volume (Fig. 5o,p). Together, these findings demonstrate that sustained CM-specific *Sdhb* deletion enhances proliferation and reduces scar formation after MI, yet fails to restore cardiac function, indicating that CM proliferation alone is insufficient for functional regeneration and the beneficial effects of transient, systemic malonate treatment require coordination across multiple cardiac cell types.

### CM-specific *Sdhb* deletion induces transient metabolic, mitochondrial and epigenetic remodeling in CMs

**Fig. 6:**
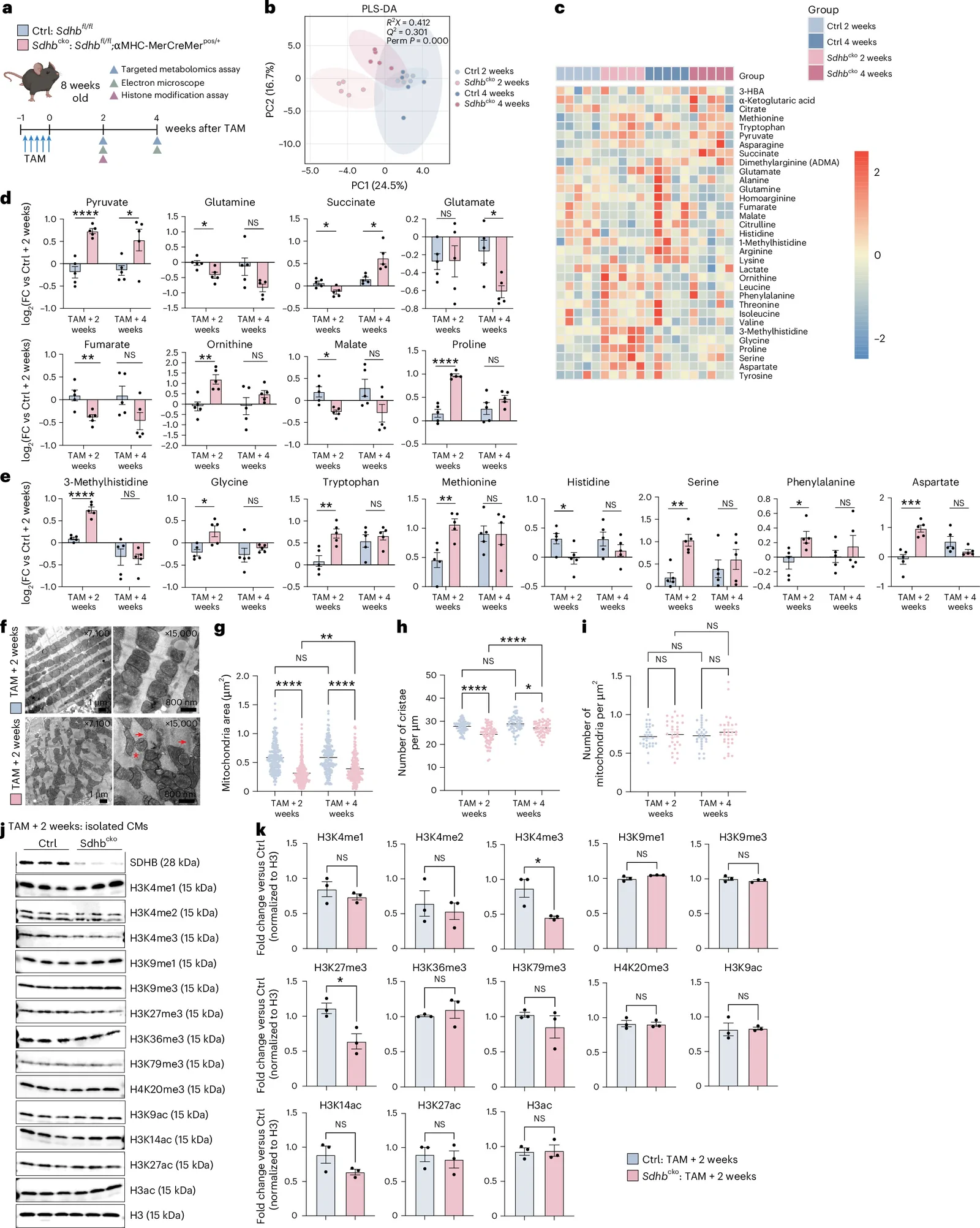
CM-specific *Sdhb* deletion induces transient metabolic, mitochondrial and epigenetic remodeling in CMs. **a**, Schematic of the experimental design for mechanistic analysis after CM-specific *Sdhb* deletion. **b**, PLS-DA plot showing metabolic separation among control and *Sdhb*^cko^ hearts at 2 and 4 weeks after TAM. Each dot represents an individual mouse. PLS-DA was performed with cross-validation and model significance was assessed using a 500-permutation test. **c**, Heat map showing relative abundance of differentially regulated organic acid and amino acid metabolites across the indicated groups. **d**, Quantification of altered mitochondrial metabolic pathways in *Sdhb*^cko^ hearts (*n* = 5 mice each group; *P* values ordered TAM + 2 weeks, TAM + 4 weeks: pyruvate, *P* = 0.0001, *P* = 0.0244; glutamine, *P* = 0.0141, *P* = 0.0971; succinate, *P* = 0.0111, *P* = 0.0194; glutamate, *P* = 0.8616, *P* = 0.0177; fumarate, *P* = 0.0094, *P* = 0.0929; ornithine, *P* = 0.0078, *P* = 0.3308; malate, *P* = 0.0109, *P* = 0.0901; proline, *P* = 0.0001, *P* = 0.2000). Each dot represents an individual mouse. An unpaired two-tailed Student’s *t*-test was applied. FC, fold change. **e**, Quantification and schematic summary of amino acid pools in *Sdhb*^cko^ hearts (*n* = 5 mice each group; *P* values ordered TAM + 2 weeks, TAM + 4 weeks: 3-methylhistidine, *P* = 0.0001, *P* = 0.7148; glycine, *P* = 0.0113, *P* = 0.5109; tryptophan, *P* = 0.0052, *P* = 0.5297; methionine, *P* = 0.0036, *P* = 0.9404; histidine, *P* = 0.0326, *P* = 0.2325; serine, *P* = 0.0034, *P* = 0.5094; phenylalanine, *P* = 0.0208, *P* = 0.4009; aspartate, *P* = 0.0007, *P* = 0.1172). Each dot represents an individual mouse. An unpaired two-tailed Student’s *t*-test was applied. **f**, Representative transmission electron microscopy images of myocardium from control and *Sdhb*^cko^ hearts at 2 weeks after TAM. The arrow indicates the disassembled sarcomere; the asterisk marks the smaller mitochondria. **g**–**i**, Quantification of mitochondrial area (TAM + 2 weeks, Ctrl versus *Sdhb*^cko^, *P* = 0.0001; TAM + 4 weeks, Ctrl versus *Sdhb*^cko^, *P* = 0.0001; Ctrl, TAM + 2 weeks versus TAM + 4 weeks, *P* = 0.9998; *Sdhb*^cko^, TAM + 2 weeks versus TAM + 4 weeks, *P* = 0.0086) (**g**), number of cristae (TAM + 2 weeks, Ctrl versus *Sdhb*^cko^, *P* = 0.0001; TAM + 4 weeks, Ctrl versus *Sdhb*^cko^, *P* = 0.0164; Ctrl, TAM + 2 weeks versus TAM + 4 weeks, *P* = 0.2238; *Sdhb*^cko^, TAM + 2 weeks versus TAM + 4 weeks, *P* = 0.0001) (**h**) and mitochondrial density (TAM + 2 weeks, Ctrl versus *Sdhb*^cko^, *P* = 0.9192; TAM + 4 weeks, Ctrl versus *Sdhb*^cko^, *P* = 0.7411; Ctrl, TAM + 2 weeks versus TAM + 4 weeks, *P* = 0.9943; *Sdhb*^cko^, TAM + 2 weeks versus TAM + 4 weeks, *P* = 0.9228) (**i**) in control and *Sdhb*^cko^ hearts at 2 and 4 weeks after TAM (*n* = 3 mice each group). A two-way ANOVA with Tukey’s post hoc test was applied. **j**, Representative western blots showing SDHB and histone modification levels in isolated CMs from control and *Sdhb*^cko^ hearts at 2 weeks after TAM. Total H3 was used as a loading control for histone marks. **k**, Quantification of histone methylation and acetylation marks in isolated CMs from control and *Sdhb*^cko^ hearts at 2 weeks after TAM (*n* = 3 mice each group; *P* values left to right, top to bottom: *P* = 0.3874, 0.6359, 0.0322, 0.0819, 0.3795, 0.0230, 0.5187, 0.3457, 0.8721, 0.8978, 0.1266, 0.6816 and 0.8903). Each dot represents an individual mouse. An unpaired two-tailed Student’s *t*-test was applied. Data are presented as the mean ± s.e.m. *P < 0.05, **P < 0.01, ***P < 0.001 and ****P < 0.0001. Scale bars are indicated. Source data

**Extended Data Fig. 6:**
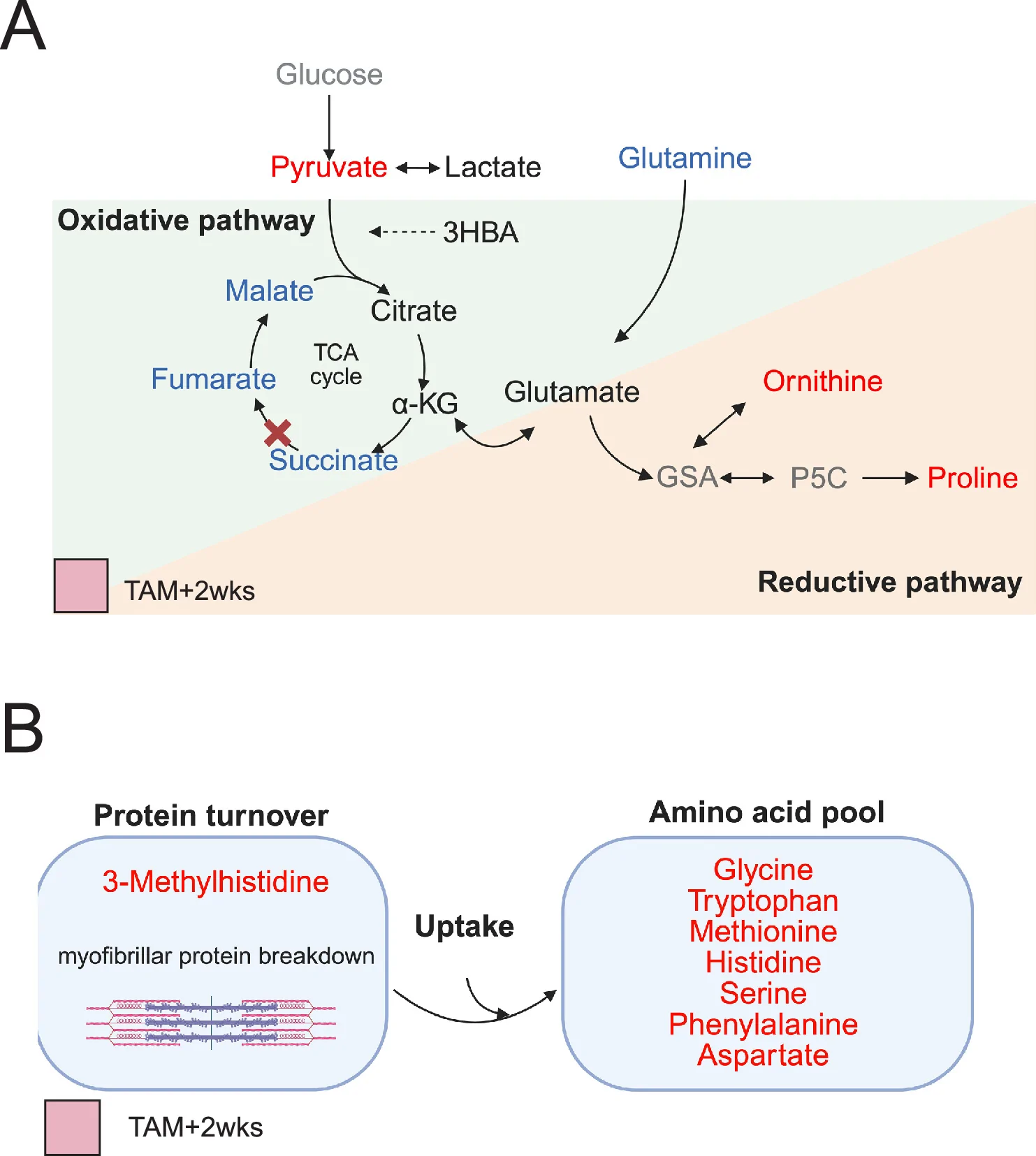
CM-specific *Sdhb* deletion alters metabolic pathways in cardiomyocytes. **a**, Schematic summary of altered mitochondrial metabolic pathways in *Sdhb*^cko^ hearts from Fig. 6d. **b**, Schematic summary of amino acid pools in *Sdhb*^cko^ hearts from Fig. 6e.

Next, we sought to define the mechanisms associated with the transient proliferative response observed in *Sdhb*^cko^ hearts at 2 weeks after induction. To this end, we performed targeted metabolomic profiling, ultrastructural analysis of mitochondria and assessment of histone methylation and acetylation marks at 2 and 4 weeks after TAM induction (Fig. 6a). Targeted metabolomic analysis of myocardial organic acids and amino acids revealed distinct metabolic remodeling in *Sdhb*^cko^ hearts. Partial least squares discriminant analysis (PLS-DA) showed clear separation of the *Sdhb*^cko^ group at 2 weeks after induction from the other three groups, including *Sdhb*^*fl/fl*^ controls at 2 and 4 weeks and *Sdhb*^cko^ hearts at 4 weeks after induction (Fig. 6b,c). Pathway analysis indicated that the major metabolic changes occurred in mitochondrial oxidative and reductive pathways (Fig. 6d and Extended Data Fig. 6a). At 2 weeks after induction, key metabolites of the oxidative TCA cycle, including succinate, fumarate and malate, were reduced in *Sdhb*^cko^ hearts. By contrast, metabolites associated with reductive metabolism, including proline and ornithine, were increased. Notably, these metabolic alterations were either reversed or stabilized by 4 weeks after induction, coinciding with the reduction in proliferation.

In addition to changes in mitochondrial metabolism, *Sdhb*^cko^ hearts showed marked remodeling of the amino acid pool at 2 weeks after induction. The myofibrillar protein breakdown marker 3-methylhistidine was significantly increased, accompanied by elevated levels of several amino acids, including glycine, tryptophan, methionine, serine, phenylalanine and aspartate (Fig. 6e and Extended Data Fig. 6b). These changes were largely stabilized by 4 weeks after induction, suggesting a transient disruption of protein turnover and amino acid homeostasis during the proliferative phase.

We next examined mitochondrial and sarcomeric ultrastructure by electron microscopy. At 2 weeks after induction, *Sdhb*^cko^ hearts displayed smaller and disorganized mitochondria, together with disrupted sarcomere architecture, features consistent with a less mature CM state (Fig. 6f, arrows and asterisks). Quantitative analysis confirmed a significant reduction in both number of cristae and mitochondrial area at this time point, with partial recovery by 4 weeks after induction (Fig. 6g,h). By contrast, mitochondrial density was not significantly altered between *Sdhb*^cko^ and *Sdhb*^*fl/fl*^ hearts at either time point (Fig. 6i). Together, these findings indicate that CM-specific Sdhb deletion induces a transient metabolic shift toward reductive metabolism and an immature mitochondrial state, which closely coincides with the window of CM proliferative activity.

Given the metabolic reprogramming observed in *Sdhb*^cko^ CMs, along with the distinct transcriptional signatures and changes in chromatin accessibility revealed by our integrated snRNA-seq and snATAC-seq analysis, we hypothesized that SDH inhibition may trigger epigenetic remodeling as a downstream effector of metabolic reprogramming. In particular, the shift in key metabolites may influence the activity of chromatin-modifying enzymes by serving as essential cofactors or substrates^52^. This raised the possibility that metabolic rewiring in *Sdhb*^cko^ CMs reshapes histone modification states to establish conditions permissive for proliferation. To investigate this, we assessed global histone methylation and acetylation marks associated with transcriptional activation and repression in CMs isolated from *Sdhb*^cko^ and *Sdhb*^*fl/fl*^ hearts at 2 weeks after induction. We identified a significant reduction in H3K4me3 and H3K27me3 levels, while all other histone lysine modifications analyzed remained unchanged (Fig. 6j,k).

**Extended Data Fig. 7:**
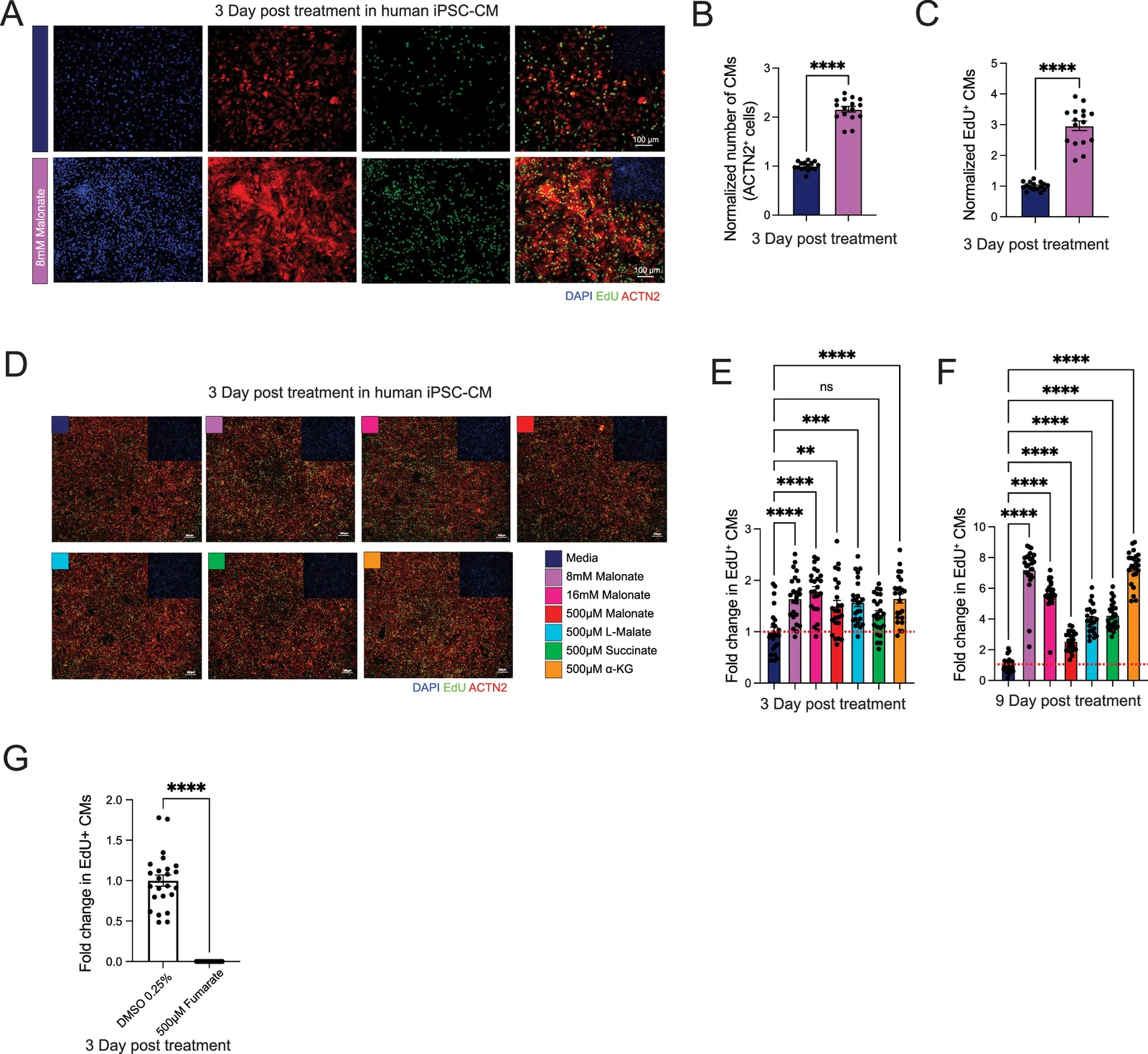
Malonate and selected TCA cycle metabolites promote proliferation of human iPSC-derived cardiomyocytes. **a**, Representative immunofluorescence images of human iPSC-derived cardiomyocytes treated with control medium or 8 mM malonate for 3 days. Cardiomyocytes were identified by ACTN2 staining, and proliferating cells were labeled by EdU incorporation. **b, c**, Quantification of normalized ACTN2+ cardiomyocyte number (P = 0.0001) (**b**) and normalized EdU+ cardiomyocytes number (P = 0.0001) (**c**) after 3 days of 8 mM malonate treatment. n = 16 independent wells each group. Unpaired two-tailed Student’s t-test was applied. **d**, Representative immunofluorescence images of human iPSC-derived cardiomyocytes treated with control medium, 8 mM malonate, 16 mM malonate, 500 μM malonate, 500 μM L-malate, 500 μM succinate, or 500 μM α-KG for 3 days. **e, f**, Quantification of fold change in EdU+ cardiomyocytes after 3 days (Media vs. 8 mM Malonate: P = 0.0001; Media vs. 16 mM Malonate: P = 0.0001; Media vs. 500 μM Malonate: P = 0.0028; Media vs. 500 μM L-Malate: P = 0.0003; Media vs. 500 μM Succinate: P = 0.1905; Media vs. 500 μM α-KG: P = 0.0001) (**e**) and 9 days (Media vs. 8 mM Malonate: P = 0.0001; Media vs. 16 mM Malonate: P = 0.0001; Media vs. 500 μM Malonate: P = 0.0001; Media vs. 500 μM L-Malate: P = 0.0001; Media vs. 500 μM Succinate: P = 0.0001; Media vs. 500 μM α-KG: P = 0.0001) (**f**) of metabolite or malonate treatment. Cardiomyocytes were identified by ACTN2 staining, and EdU+ cardiomyocytes were quantified among ACTN2+ cells. n = 24 independent wells each group. Two-way ANOVA with Tukey’s post hoc test was applied. **g**, Quantification of fold change in EdU+ cardiomyocytes after 3 days of 500 μM fumarate treatment compared with DMSO control (P = 0.0001). n = 24 independent wells each group. Unpaired two-tailed Student’s t-test was applied. Data are presented as mean ± s.e.m. ns, not significant; *P < 0.05, **P < 0.01, ***P < 0.001, ****P < 0.0001. Scale bars are indicated in the images. Source data

We next sought to identify candidate metabolites that may functionally link metabolic remodeling to CM proliferation. Using human induced pluripotent stem cell (hiPSC)-derived CMs^53,54^, we tested TCA cycle metabolites altered in *Sdhb*^cko^ hearts at 2 weeks after induction, including succinate, fumarate and malate, with α-ketoglutarate and malonate included as positive controls. Malonate increased iPSC-derived CM proliferation and total CM number at 3 days after treatment across multiple concentrations (500 μM, 8 mM and 16 mM), as assessed by EdU incorporation and ACTN2 staining^55^, with a sustained effect observed at 9 days (Extended Data Fig. 7a–c). Among the other TCA cycle metabolites tested, succinate and malate also increased iPSC-derived CM proliferation at 9 days after treatment, whereas fumarate induced marked CM toxicity and cell loss as early as 3 days after treatment (Extended Data Fig. 7d–g). These findings identify succinate and malate as candidate proproliferative metabolites and suggest that specific metabolites downstream of SDH inhibition contribute to CM proliferation through a metabolic–epigenetic–transcriptional axis.

### Myofibroblast-specific *Sdhb* deletion blocks fibroblast activation after MI

Our snRNA-seq and snATAC-seq revealed that, following malonate treatment, fibroblasts exhibited one of the most pronounced transcriptional and chromatin accessibility changes among nonmyocyte populations (Fig. 3). Remarkably, the myofibroblast subpopulation, characterized by high profibrotic gene expression after MI, was significantly diminished in malonate-treated hearts compared to saline-treated controls. Thus, we hypothesized that SDH inhibition actively limits fibroblast activation and fibrotic remodeling after MI, contributing to the multicellular response to SDH inhibition.

**Fig. 7:**
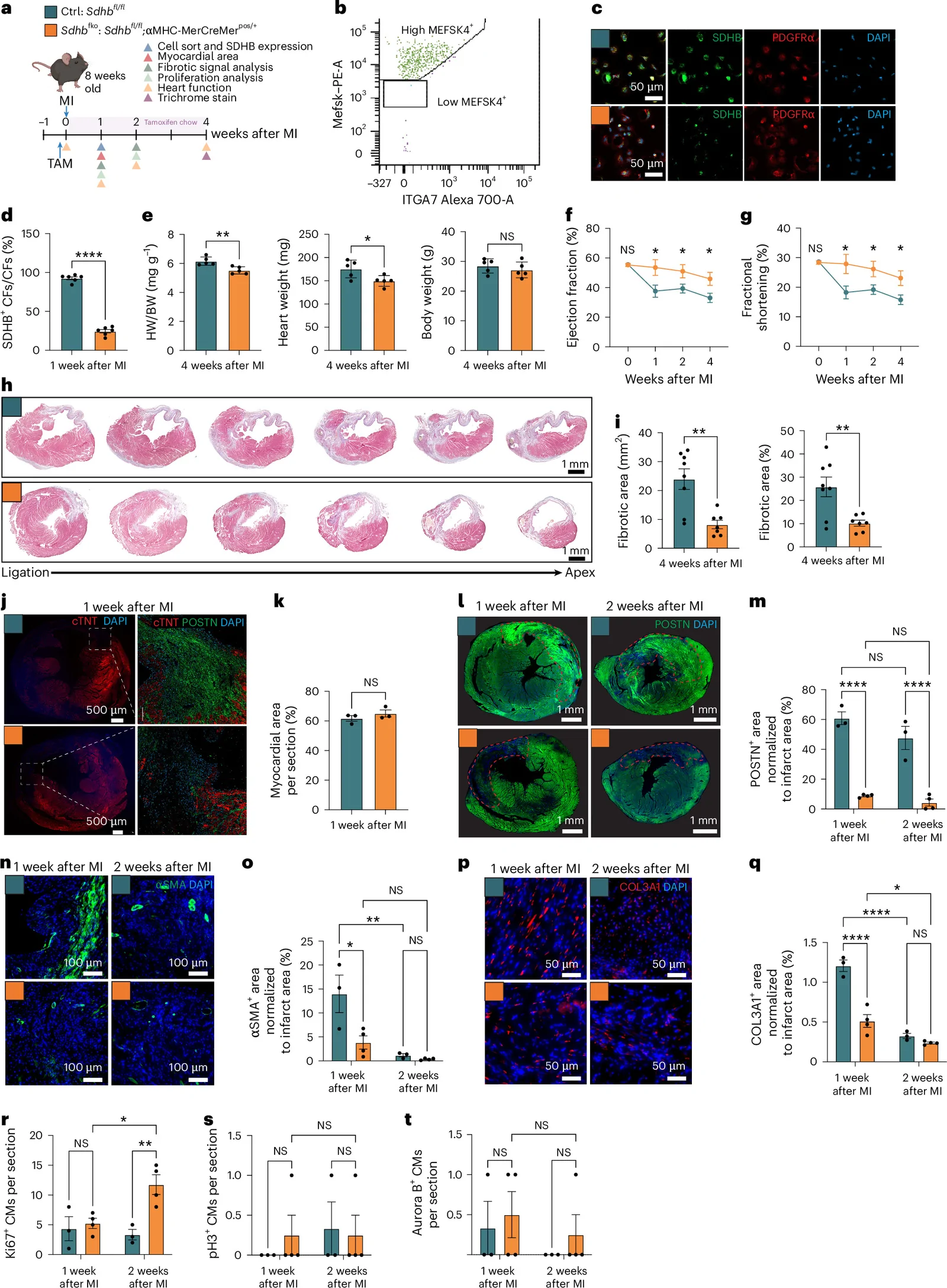
Myofibroblast-specific *Sdhb* deletion improves cardiac function after MI by suppressing fibroblast activation and fibrosis. **a**, Schematic of the experimental strategy for myofibroblast-specific *Sdhb* deletion after MI. **b**, Representative flow cytometry plot showing MEFSK4-based sorting of CFs from injured myocardium, with ITGA7 costaining used to exclude mural cell contamination. **c**,**d**, Representative immunostaining images (**c**) and quantification (*P* = 0.0001) (**d**) of SDHB^+^ PDGFRα^+^ CFs among total sorted CFs from control and *Sdhb*^fko^ hearts at 1 weeks after MI (*n* = 6 mice each group). Each dot represents an independent biological replicate. An unpaired two-tailed Student’s t-test was applied. **e**, Quantification of the HW/BW ratio (*P* = 0.0061), HW (*P* = 0.0320) and BW (*P* = 0.4315) in control and *Sdhb*^fko^ mice at 4 weeks after MI (*n* = 5 mice each group). Each dot represents an individual mouse. An unpaired two-tailed Student’s *t*-test was applied. **f**,**g**, Serial echocardiographic quantification of ejection fraction (*P* = 0.8866, 0.0328, 0.0441 and 0.0311) **f**, and fractional shortening (*P* = 0.9589, 0.0245, 0.0335 and 0.0246) (**g**) before MI and at 1, 2 and 4 weeks after MI (order of *P* values). Ctrl, *n* = 8 mice; *Sdhb*^fko^, *n* = 6 mice. An unpaired two-tailed Student’s *t*-test was applied. **h**, Representative Masson’s trichrome-stained serial heart sections from control and *Sdhb*^fko^ mice at 4 weeks after MI, arranged from the ligation site to the apex. **i**, Quantification of fibrotic area (*P* = 0.0019) and fibrotic area percentage (*P* = 0.0055) at 4 weeks after MI (*n* = 8 Ctrl, *n* = 7 *Sdhb*^fko^ mice). Each dot represents the average of six sections from an individual mouse. An unpaired two-tailed Student’s *t*-test was applied. **j**,**k**, Representative cTNT staining (**j**) and quantification (**k**) of surviving myocardial area at 1 week after MI (*n* = 3 mice each group, *P* = 0.3395). Each dot represents one section collected 250 μm from the ligation level in an individual mouse. An unpaired two-tailed Student’s *t*-test was applied. **l**,**m**, Representative POSTN immunostaining images (**l**) and quantification (**m**) of POSTN^+^ area normalized to infarct area at 1 and 2 weeks after MI (1 week after MI, *n* = 3 Ctrl, *n* = 4 *Sdhb*^fko^ mice; 2 weeks after MI, *n* = 3 Ctrl, *n* = 4 *Sdhb*^fko^ mice; 1 week, Ctrl versus *Sdhb*^fko^, *P* = 0.0001; 2 weeks, Ctrl versus *Sdhb*^fko^, *P* = 0.0001; Ctrl, 1 week versus 2 weeks, *P* = 0.1867; *Sdhb*^fko^, 1 week versus 2 weeks, *P* = 0.8226). Each dot represents one section collected 250 μm from the ligation level in an individual mouse. A two-way ANOVA with Tukey’s post hoc test was applied. **n**,**o**, Representative α-SMA immunostaining images (**n**) and quantification (**o**) of α-SMA^+^ area normalized to infarct area at 1 and 2 weeks after MI (1 week after MI, *n* = 3 Ctrl, *n* = 4 *Sdhb*^fko^ mice; 2 weeks after MI, *n* = 3 Ctrl mice, *n* = 4 *Sdhb*^fko^ mice; 1 week, Ctrl versus *Sdhb*^fko^, *P* = 0.0131; 2 weeks, Ctrl versus *Sdhb*^fko^, *P* = 0.9890; Ctrl, 1 week versus 2 weeks, *P* = 0.0044; *Sdhb*^fko^, 1 week versus 2 weeks, *P* = 0.5027). Each dot represents one section collected 250 μm from the ligation level in an individual mouse. A two-way ANOVA with Tukey’s post hoc test was applied. **p**,**q**, Representative COL3A1 immunostaining images (**p**) and quantification (**q**) of COL3A1^+^ area normalized to infarct area at 1 and 2 weeks after MI (1 week after MI, *n* = 3 Ctrl, *n* = 4 *Sdhb*^fko^ mice; 2 weeks after MI, *n* = 3 Ctrl, *n* = 4 *Sdhb*^fko^ mice; 1 week, Ctrl versus *Sdhb*^fko^, *P* = 0.0001; 2 weeks, Ctrl versus *Sdhb*^fko^, *P* = 0.7099; Ctrl, 1 week versus 2 weeks, *P* = 0.0001; *Sdhb*^fko^, 1 week versus 2 weeks, *P* = 0.0206). Each dot represents one section collected 250 μm from the ligation level in an individual mouse. A two-way ANOVA with Tukey’s post hoc test was applied. **r**–**t**, Quantification of Ki67^+^ CMs (1 week, Ctrl versus *Sdhb*^fko^, *P* = 0.9679; 2 weeks, Ctrl, versus *Sdhb*^fko^, *P* = 0.0091; *Sdhb*^fko^, 1 week versus 2 weeks, *P* = 0.0267) (**r**), pH3^+^ CMs (1 week, Ctrl versus *Sdhb*^fko^, *P* = 0.8935; 2 weeks, Ctrl versus *Sdhb*^fko^, *P* = 0.9952; *Sdhb*^fko^, 1 week versus 2 weeks, *P* = 0.9999) (**s**) and Aurora B^+^ CMs (1 week, Ctrl versus *Sdhb*^fko^, *P* = 0.9693; 2 weeks, Ctrl versus *Sdhb*^fko^, *P* = 0.9075; *Sdhb*^fko^, 1 week versus 2 weeks, *P* = 0.8872) (**t**) in control and *Sdhb*^fko^ hearts at 1 and 2 weeks after MI (1 week after MI, *n* = 3 Ctrl, *n* = 4 *Sdhb*^fko^ mice; 2 weeks after MI, *n* = 3 Ctrl, *n* = 4 *Sdhb*^fko^ mice). Each dot represents one section collected 250 μm from the ligation level in an individual mouse. A two-way ANOVA with Tukey’s post hoc test was applied. Data are presented as the mean ± s.e.m. *P < 0.05, **P < 0.01, ***P < 0.001 and ****P < 0.0001. Scale bars are indicated. Source data

To directly test the role of SDH in fibroblast activation, we generated TAM-inducible *Postn*-MerCreMer^pos/+^;*Sdhb*^fl/fl^ mice (hereafter referred to as *Sdhb*^fko^; Fig. 7a). MI was induced in 8-week-old mice 1 day after a single TAM injection, followed by continuous TAM chow feeding until 4 weeks after MI. At 1 week after MI, CFs were isolated from the injured myocardial region of *Sdhb*^fko^ and *Sdhb*^*fl/fl*^ mice and sorted by flow cytometry on the basis of MEFSK4 positivity, with ITGA7 costaining used to exclude mural cell contamination (Fig. 7b and Supplementary Fig. 4). Immunostaining and quantification confirmed efficient *Sdhb* deletion in CFs, as demonstrated by a marked reduction in the proportion of SDHB^+^PDGFRα^+^ cells among total sorted fibroblasts, from 93% in *Sdhb*^fl/fl^ hearts to 24% in *Sdhb*^fko^ hearts (Fig. 7c,d). *Sdhb*^fko^ mice displayed significantly decreased heart rate and heart weight/body weight ratio compared to *Sdhb*^*fl/fl*^ mice at 4 weeks after MI, while body weight remained unchanged (Fig. 7e). *Sdhb*^fko^ mice exhibited significant functional improvement as early as 1 week after MI, sustained for up to 4 weeks after injury (Fig. 7f,g). Quantitative analysis of trichrome-stained sections shows a significant reduction in scar area in *Sdhb*^fko^ mice compared to *Sdhb*^*fl/fl*^ mice (Fig. 7h,i).

To determine the basis for the early improvement in cardiac function after MI, the surviving myocardial area was quantified by cTNT staining at 1 week after MI, which showed no significant difference between *Sdhb*^fko^ and *Sdhb*^*fl/fl*^ mice (Fig. 7j,k), suggesting that the early functional improvement was unlikely to result from enhanced myocardial protection. Interestingly, regions of myocardial loss were accompanied by reduced POSTN signal in *Sdhb*^fko^ hearts (Fig. 7j). We next examined whether this benefit was associated with suppression of the profibrotic response. *Sdhb*^fko^ hearts showed significantly reduced expression of profibrotic markers, including POSTN, COL3A1 and α-SMA, at both 1 and 2 weeks after MI, indicating robust inhibition of MI-induced myofibroblast activation (Fig. 7l–q). Because recombination in the *Sdhb*^fko^ model requires activation of POSTN-expressing myofibroblasts, these findings suggest that SDH is not required for the initial induction of the myofibroblast program but rather for its maintenance and progression following injury. Accordingly, once activated myofibroblasts undergo *Sdhb* deletion, they exhibit reduced expression of profibrotic markers and fail to maintain a fully activated myofibroblast phenotype. To further determine whether SDH inhibition in myofibroblasts affected CM proliferation, we quantified Ki67^+^, pH3^+^ and Aurora B^+^ CMs at 1 and 2 weeks after MI; however, no significant increase in CM proliferation was observed in *Sdhb*^fko^ mice (Fig. 7r–t).

**Extended Data Fig. 8:**
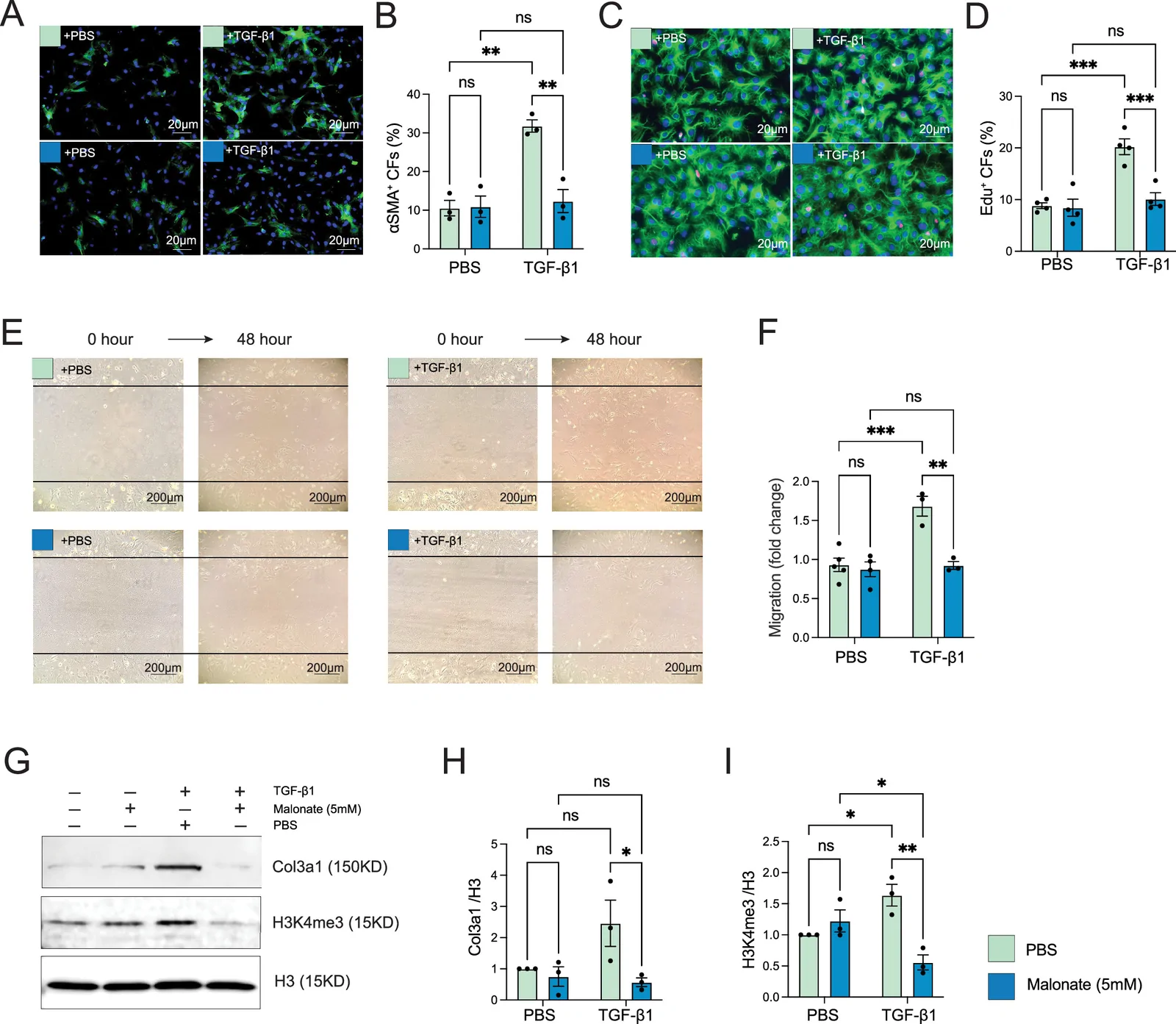
SDH inhibition by malonate suppresses TGF-β1-induced activation of neonatal mouse cardiac fibroblasts in vitro. **a-d**, Immunofluorescence analysis with quantification of the α-SMA + (PBS, PBS vs. Malonate (5 mM): P = 0.9995; TGF-β1, PBS vs. Malonate (5 mM): P = 0.0020; PBS, PBS vs. TGF-β1: P = 0.0011; Malonate (5 mM), PBS vs. TGF-β1: P = 0.9724) (**a, b**) and EdU+ (PBS, PBS vs. Malonate (5 mM): P = 0.9965; TGF-β1, PBS vs. Malonate (5 mM): P = 0.0007; PBS, PBS vs. TGF-β1: P = 0.0003; Malonate (5 mM), PBS vs. TGF-β1: P = 0.8054) (**c, d**) of neonatal mouse CFs (NMCFs), with or without 5 mM SDH inhibitor (malonate) and 10 ng/mL TGF- β1 treatments (n = 3 biological replicates). **e, f**, The migration ratio in each group. n = 5 biological replicates in PBS group; n = 4 biological replicates in PBS+malonate group; n = 3 biological replicates in TGF-β1 + PBS group; n = 3 biological replicates in TGF-β1+malonate group (PBS, PBS vs. Malonate (5 mM): P = 0.9653; TGF-β1, PBS vs. Malonate (5 mM): P = 0.0017; PBS, PBS vs. TGF-β1: P = 0.0007; Malonate (5 mM), PBS vs. TGF-β1: P = 0.9840). **g**-**i**, Western blot images (**g**) and quantification (**h, i**) of Col3a1 (PBS, PBS vs. Malonate (5 mM): P = 0.9721; TGF-β1, PBS vs. Malonate (5 mM): P = 0.0451; PBS, PBS vs. TGF-β1: P = 0.1290; Malonate (5 mM), PBS vs. TGF-β1: P = 0.9880) and H3K4me3 (PBS, PBS vs. Malonate (5 mM): P = 0.6801; TGF-β1, PBS vs. Malonate (5 mM): P = 0.0024; PBS, PBS vs. TGF-β1: P = 0.0457; Malonate (5 mM), PBS vs. TGF-β1: P = 0.0374) in each group (n = 3 biological replicates). Histone 3 (H3) was used as a loading control. Two-way ANOVA with Tukey’s post hoc test was applied. Data are presented as mean ± s.e.m. ns, not significant; *P < 0.05, **P < 0.01, ***P < 0.001, ****P < 0.0001. Scale bars are indicated in the images. Source data

We also investigated whether SDH inhibition by malonate could suppress CF activation in vitro. Neonatal mouse CFs (NMCFs) were treated with malonate for 48 h, either at baseline or following stimulation with the profibrotic cytokine TGFβ1. Malonate treatment significantly reduced TGFβ1-induced activation of NMCFs (Extended Data Fig. 8a,b), proliferation (Extended Data Fig. 8c,d) and migration (Extended Data Fig. 8e,f). Malonate treatment led to a significant reduction in H3K4me3 alongside reduced COL3A1 protein levels, suggesting that SDH inhibition suppresses myofibroblast activation in part through H3K4me3-dependent transcriptional repression of profibrotic gene expression (Extended Data Fig. 8g–i).

Together, these findings demonstrate that myofibroblast-specific SDH inhibition suppresses fibroblast activation and fibrotic remodeling after MI through epigenetic mechanisms, resulting in improved cardiac function independently of CM proliferation. Combined with the CM-specific knockout data, these results establish that SDH inhibition exerts distinct and complementary effects in CMs and fibroblasts, supporting a multicellular mechanism underlying the regenerative response to transient systemic malonate treatment.

### Malonate treatment after MI alters the cardiac chromatin state through H3k4me3 and H3k27me3 remodeling

**Fig. 8:**
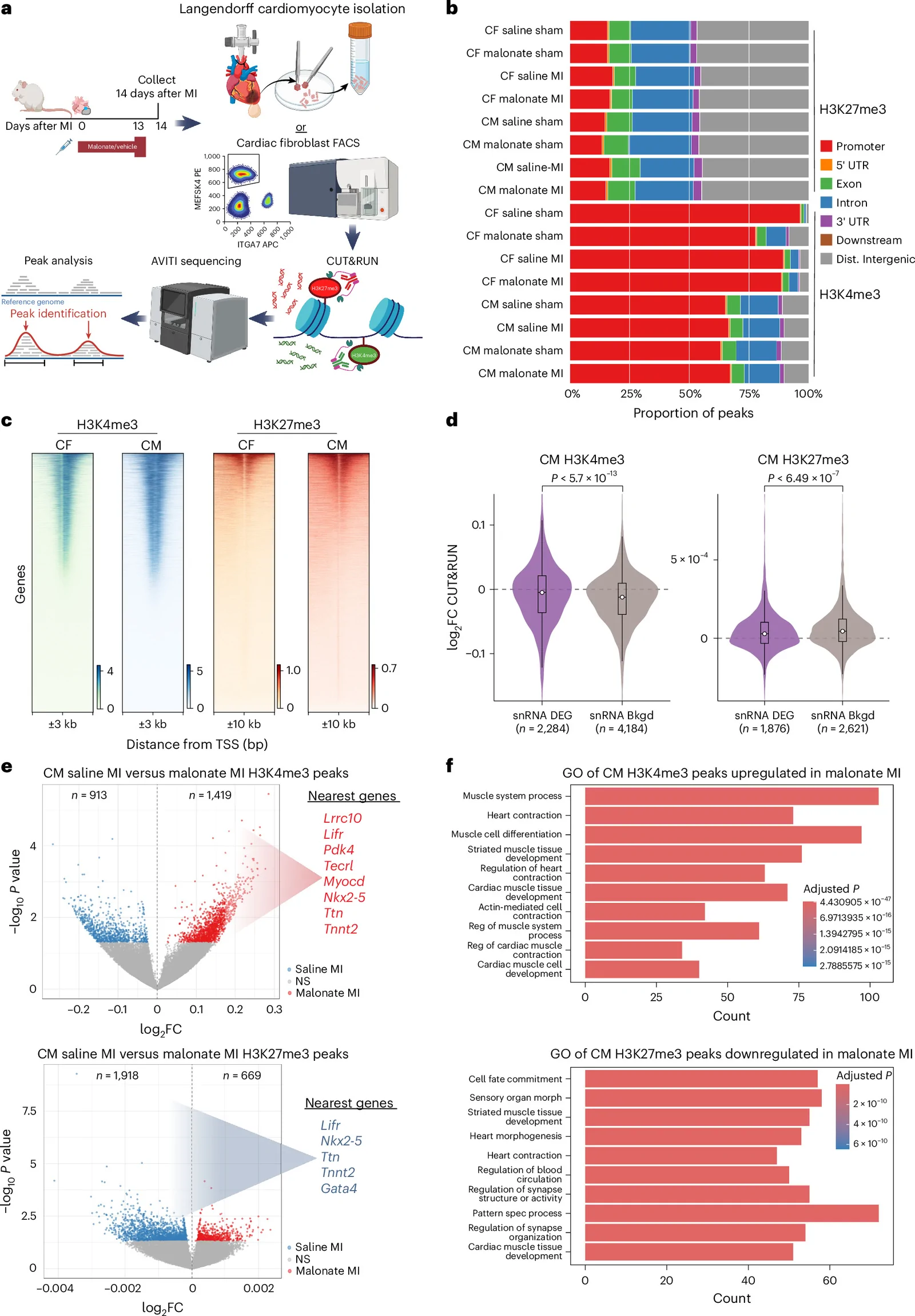
Malonate treatment remodels active and repressive chromatin states in CMs following MI. **a**, Experimental workflow. Mice were subjected to MI and treated with either saline vehicle or malonate (*n* = 2 biological replicates per group). **b**, Genomic annotation of H3K4me3 and H3K27me3 CUT&RUN peaks across all cell types and experimental conditions. H3K4me3 peaks were predominantly promoter associated, whereas H3K27me3 peaks exhibited broader genomic distributions. **c**, Aggregate CUT&RUN signal centered on TSSs for H3K4me3 (±3 kb) and H3K27me3 (±10 kb) in CFs and CMs, demonstrating the expected promoter-enriched distribution of H3K4me3 and broad repressive domains of H3K27me3. **d**, CUT&RUN signal changes at malonate MI-responsive CM genes identified by snRNA-seq. Violin plots show the log_2_FC in CUT&RUN signal (malonate MI/saline MI) for differentially expressed genes (DEGs; purple) compared to matched background (Bkgd) gene sets (gray), controlling for expression level and gene body length. The center line of each embedded boxplot indicates the median; box bounds represent the 25th and 75th percentiles (interquartile range, IQR); whiskers extend to 1.5× the IQR from the box edges. H3K4me3 signal was significantly higher at DEGs relative to background (two-sided Wilcoxon rank-sum test, *P* = 5.74 × 10^−13^. DEG median log_2_FC = −0.005, IQR: −0.036 to 0.021; background median = −0.012, IQR −0.039 to 0.010; *n* = 2,284 DEGs and 4,184 matched background genes; no adjustment for multiple comparisons). H3K27me3 signal was significantly lower at DEGs relative to background (*P* = 6.49 × 10^−7^, *r* = 0.087; DEG median = 2.8 × 10^−5^, IQR: −3.3 × 10^−5^ to 1.02 × 10^−4^; background median = 4.5 × 10^−5^, IQR: −2.1 × 10^−5^ to 1.2 × 10^−4^; *n* = 1,876 DEGs and 2,621 background genes). **e**, Differential H3K4me3 (top) and H3K27me3 (bottom) peak analysis in CMs comparing malonate MI and saline MI hearts. Volcano plots display differential chromatin accessibility identified by DESeq2 using nominal *P*  < 0.05; log_2_FC estimates were stabilized using adaptive shrinkage (ashr). The statistical test was a two-sided Wald test as implemented in DESeq2; no adjustment for multiple comparisons was applied. Peaks enriched in malonate-treated hearts are shown in red and peaks enriched in saline-treated hearts are shown in blue. A total of 1,419 H3K4me3 peaks and 696 H3K27me3 peaks were increased, while 913 H3K4me3 peaks and 1,918 H3K27me3 peaks were decreased in malonate MI CMs. Representative nearest genes associated with malonate MI-enriched H3K4me3 peaks are indicated with red text and saline-enriched H3K27me3 peaks are indicated with blue text. **f**, GO enrichment analysis of genes associated with H3K4me3 peaks enriched (top) and H3K27me3 peaks decreased (bottom) in malonate MI CMs, performed using Benjamini–Hochberg correction (adjusted *P*  < 0.05). H3K4me3-enriched biological processes include cardiac muscle development, muscle differentiation and regulation of contractile function. H3K27me3-decreased biological processes include cardiac morphogenesis, cell fate commitment, tissue remodeling and regulation of synapse organization.

To investigate whether malonate-induced chromatin changes support cardiac repair, we performed CUT&RUN profiling of the active histone mark H3K4me3 and the repressive histone mark H3K27me3 in purified CMs and CFs isolated from MI and sham hearts treated with malonate or saline for 14 days (Fig. 8a). Both marks demonstrated expected genome-wide distributions, with H3K4me3 enriched at promoter-proximal regions and H3K27me3 exhibiting broader domains surrounding TSSs (Fig. 8b,c and Supplementary Fig. 5). To determine whether transcriptional programs previously identified by snRNA-seq were accompanied by corresponding chromatin changes, we compared the CUT&RUN signal at malonate MI-responsive CM genes. Differentially expressed genes displayed significantly increased H3K4me3 and decreased H3K27me3 densities relative to background gene sets, supporting a link between malonate-dependent transcriptional and epigenetic remodeling following MI (Fig. 8d). Consistent with this observation, differential peak analysis identified 2,332 H3K4me3-associated loci and 2,587 H3K27me3-associated loci that differed between saline MI-treated and malonate MI-treated CMs (Fig. 8e and Supplementary Table 8). H3K4me3 sites enriched in malonate-treated CMs mapped to regulatory regions of malonate MI-responsive CM genes identified by snRNA-seq, including *Lrrc10*, *Lifr*, *Pdk4* and *Tecrl*, and were associated with pathways governing cardiac muscle development, muscle differentiation and contractile function (Fig. 8f and Supplementary Table 8). By contrast, H3K27me3 peaks reduced in malonate MI-treated CMs mapped to programs involved in cardiac morphogenesis, cell fate commitment and regulation of tissue remodeling. Together, these findings indicate that malonate treatment coordinately remodels H3K4me3 and H3K27me3 around malonate-responsive genes identified by snRNA-seq, supporting epigenetic regulation of cardiac developmental and tissue remodeling programs in the post-infarct heart.

## Discussion

**Extended Data Fig. 9:**
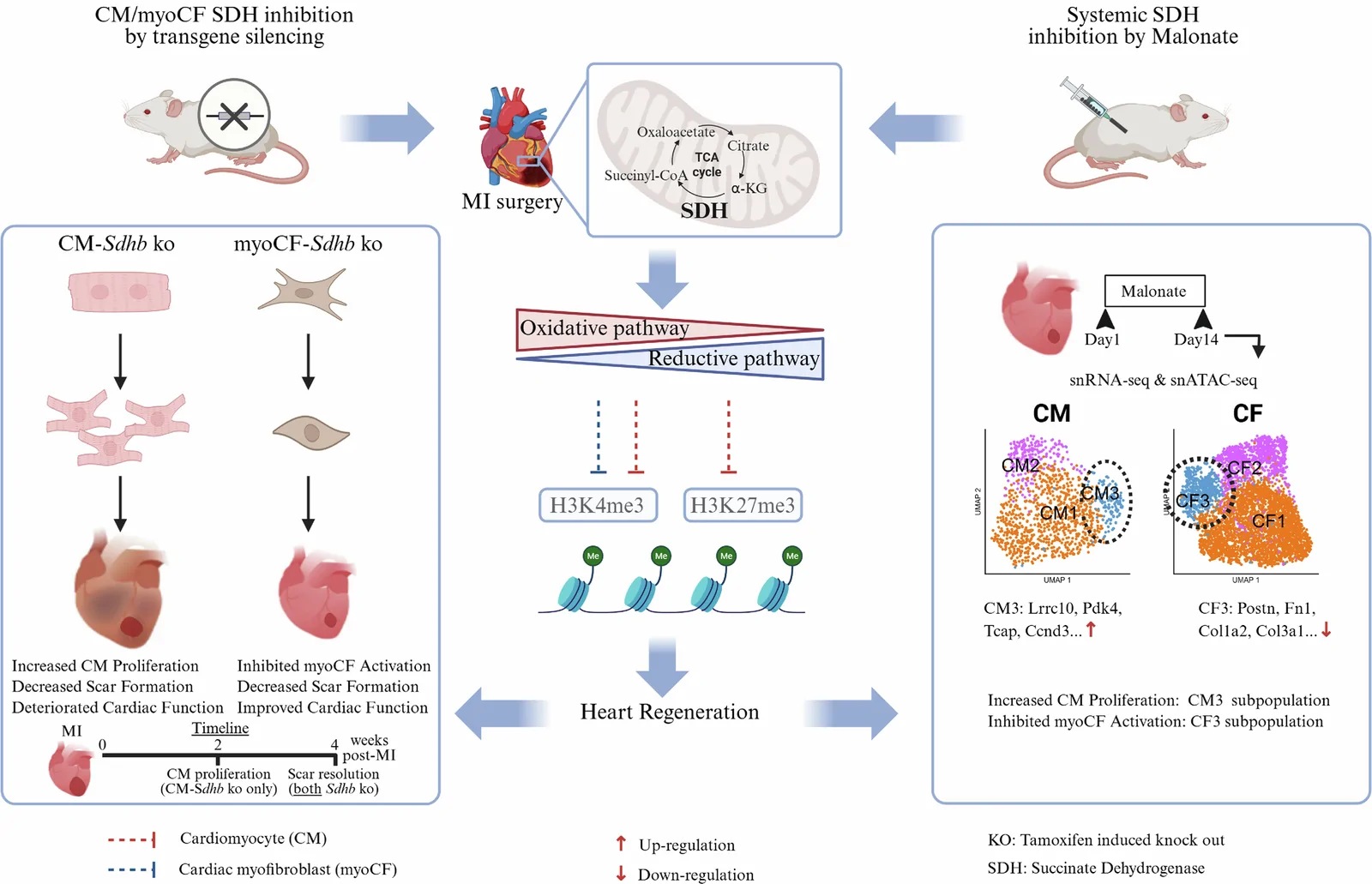
Model of SDH inhibition during heart regeneration. Schematic illustrating the cell-specific effects of SDH inhibition on cardiac regeneration, including promotion of CM proliferation and suppression of myofibroblast activation. These effects are mediated through coordinated metabolic and epigenetic reprogramming of the heart.

Transient inhibition of SDH with malonate reactivates regenerative programs in the adult heart, yet the cellular and molecular mechanisms underlying this response have remained undefined. We combined single-nucleus transcriptomic and epigenomic profiling with cell-specific genetic models and histone-resolved chromatin profiling, revealing that SDH inhibition drives CMs toward a partially immature, proliferative state while suppressing fibroblast activation and promoting an antifibrotic phenotype. These findings support a model in which transient metabolic reprogramming induces a multicellular shift from a fibrotic injury response toward regenerative features of the neonatal heart (Extended Data Fig. 9).

Mechanistically, SDH inhibition triggered reductive mitochondrial metabolism in CMs and remodeling of TCA cycle intermediates and amino acid pools. These metabolic changes coincided with reduced global H3K4me3 and H3K27me3 and increased chromatin accessibility at regeneration-associated loci. CUT&RUN profiling demonstrated that malonate treatment increased H3K4me3 at cardiac developmental genes while reducing H3K27me3 over tissue remodeling programs, linking SDH inhibition to epigenetic regulation of cardiac developmental and tissue remodeling programs. In CFs, SDH inhibition similarly reduced H3K4me3 and suppressed myofibroblast activation. Together, these data define a metabolic–epigenetic mechanism by which SDH inhibition reshapes chromatin states to favor regeneration over scarring (Extended Data Fig. 9). Moreover, succinate and malate were sufficient to promote hiPSC-derived CM proliferation, supporting a role for TCA cycle intermediates in this response.

The cell-specific *Sdhb*-knockout models provided further mechanistic insight and, importantly, revealed why genetic deletion does not phenocopy transient malonate treatment. CM-specific deletion induced a transient burst of CM proliferation at baseline and after MI and reduced scar area but failed to restore cardiac function. These findings indicate that CM proliferation alone is insufficient for functional recovery and highlight the importance of the mode and duration of SDH inhibition. Two features distinguish malonate from genetic *Sdhb* deletion; malonate produces transient, reversible inhibition and acts across multiple cell types, whereas *Sdhb* deletion permanently disables the complex.

In our prior work, malonate-treated hearts restored cardiac function by 28 days after MI, 2 weeks after treatment withdrawal, consistent with a recovery phase in which CMs reengage oxidative metabolism and undergo maturation once the metabolic block is relieved^6^. This supports a biphasic model, in which transient suppression of fatty acid oxidation and oxidative metabolism enables CM cell-cycle reentry^8^, while subsequent restoration of oxidative metabolism supports redifferentiation and completion of regeneration^56^. The malonate-responsive CM3 population was captured in a transitional state between glycolytic and toward fatty acid oxidative programs, potentially representing CMs progressing through this metabolic transition. Sustained genetic SDH loss precludes restoration of normal SDH activity and may, therefore, impair this recovery phase. Sustained SDH loss may also impose additional metabolic constraints; recent work demonstrates that SDH inhibition can limit pyrimidine biosynthesis and induce replication stress, such that elevated aspartate, which we observed in *Sdhb*-knockout hearts, is insufficient to sustain proliferation^57^. Together, these findings suggest that transient, competitive SDH inhibition uniquely permits both the proliferative and the subsequent maturation phases required for functional regeneration, whereas sustained deletion induces only transient proliferation and ultimately compromises survival.

In contrast, myofibroblast-specific *Sdhb* deletion attenuated scar formation and enhanced cardiac function after MI without inducing CM proliferation. These findings identify fibroblasts as a major cellular target of SDH inhibition. Recent work demonstrated that nuclear ATP citrate lyase promotes the myofibroblast gene program through histone acetylation-dependent chromatin remodeling following cardiac injury^58^. Consistent with this concept of metabolic regulation of fibroblast fate, SDH inhibition induced chromatin and histone methylation changes associated with suppression of myofibroblast activation. Together, these studies support a model in which metabolic remodeling converges on chromatin regulation to control fibrotic remodeling after injury.

The contrasting cell-specific models further reveal complementary effects of SDH inhibition; CM-specific *Sdhb* deletion enhanced CM proliferation without restoring cardiac function, whereas myofibroblast-specific *Sdhb* deletion improved cardiac function without inducing CM proliferation. These findings support a model in which transient, systemic SDH inhibition coordinates regenerative and antifibrotic responses across multiple cardiac cell types to achieve functional recovery. Longer-term functional studies of the cell-specific knockout models would further define the durability of their respective phenotypes, as well as the cause of mortality following CM-specific *Sdhb* deletion.

While our findings define metabolic and epigenetic mechanisms underlying the regenerative response to transient SDH inhibition, several questions remain. Although our cell-specific knockout models define the roles of SDH in CMs and fibroblasts, they do not capture other cardiac cell types that likely contribute to regeneration. Notably, myeloid populations displayed distinct transcriptional signatures between treatment groups, consistent with activation of tissue-resident macrophages that are known to promote cardiac recovery after injury^59–61^ (Extended Data Fig. 2b). Additionally, our integrated single-nucleus and CUT&RUN analyses were performed at a single time point (14 days after MI), potentially missing earlier events that initiate regeneration. Temporal profiling can help to resolve cell state transitions and intercellular signaling. Furthermore, our metabolomic profiling measured steady-state metabolite pools without isotope tracing or flux analysis. Succinate and malate were reduced in *Sdhb*^cko^ hearts yet promoted proliferation in hiPSC-derived CMs, suggesting increased use or turnover rather than reduced availability. Flux-based approaches are needed to resolve how these intermediates are routed during regeneration. Lastly, the long-term safety of transient SDH inhibition, particularly with respect to CM maturation, electrical integration and arrhythmogenic risk, remains unknown.

Together, these results identify SDH as a metabolic target linking mitochondrial function with epigenetic regulation to control cardiac regenerative capacity. Transient metabolic intervention may drive endogenous regenerative mechanisms without permanent reprogramming. This work establishes metabolic–epigenetic crosstalk as a therapeutic axis for reactivating developmental programs in the injured adult myocardium.

## Methods

### Animal models

*Sdhb* floxed (*Sdhb*^*fl/fl*^) mice (C57BL/6J background) were provided by J. Favier at INSERM. CD-1 wild-type mice were purchased from Charles River Laboratories. αMHC-MerCreMer mice (stock number 005657) and *Postn*-MerCreMer mice (stock number 029645) were obtained from the Jackson Laboratory. Primers used for genotyping included *Sdhb*fl forward (5′-CCATGCCAGTAACCCCAGAATTGG-3′), *Sdhb*^*f*^*l* reverse (5′-GTCCCACCTCACTGTAGGTCAC-3′), aMHC-MerCreMer forward (5′-TGCCGTCATTACCGGTCGATG-3′), αMHC-MerCreMer reverse (5′-CCATGAGTGAACGAACCTGGTC-3′), *Postn*-MerCreMer forward (5′-GGTGGGACATTTGAGTTGCT-3′) and Postn-MerCreMer reverse (5′-CCTTGCAATAAGTAAAACAGCTC-3′). The *Sdhb*^*fl/fl*^ mice were crossed with αMHC-MerCreMer mice to generate *Sdhb*^*fl/fl*^;αMHC-MerCreMer mice (*Sdhb*^cko^) with CM-specific knockout of *Sdhb*. The *Sdhb*^*fl/fl*^ mice were crossed with *Postn-cre* mice to generate *Sdhb*^*fl/fl*^;*Postn*-MerCreMer mice (*Sdhb*^fko^) with myofibroblast-specific knockout of *Sdhb*. Age-matched *Sdhb*^*fl/fl*^ mice with normal expression of *Sdhb* were used as control mice. All experimental procedures involving animals were approved by the Institutional Animal Care and Use Committee of Sanford Burnham Prebys and the University of Wisconsin-Madison. Experiments were conducted using age-matched mice, which were housed in individually ventilated cages under a 12-h light–dark cycle. Mice had ad libitum access to food and water.

For snRNA-seq, snATAC-seq and CUT&RUN, 8-week-old male CD-1 mice underwent MI or sham surgery followed by daily i.p. dimethyl malonate (Sigma, 108-59-8) or saline for 14 days; hearts were collected on day 14 and snap-frozen^6^. For basal *Sdhb*^cko^ studies, TAM (75 mg kg^−1^, i.p.) was given for five consecutive days and hearts were collected 2 or 4 weeks later. For MI studies in *Sdhb*^cko^ and *Sdhb*^fko^ mice, TAM (120 mg kg^−1^, i.p.) was given 1 day before MI, followed by TAM chow from day 1 after MI until collection. *Sdhb*^*fl/fl*^ littermates received identical TAM treatment and both sexes were used in all experiments.

### Drug administration

Dimethyl malonate (100 mg kg^−1^; Sigma, 108-59-8) or saline was injected i.p. daily for 14 days after MI^6^. TAM (30 mg ml^−1^; Sigma, T5648) was prepared in corn oil and administered i.p. BrdU (0.25 mg ml^−1^; MP Biomedical, B9285) was given in drinking water from day 7 after MI until collection. EdU (50 mg kg^−1^; MedChemExpress, HY-118411) was injected i.p. in saline.

### Adult mouse MI

MI was induced in 8-week-old mice by permanent left anterior descending (LAD) ligation. Mice were anesthetized with 2–4% isoflurane, intubated and mechanically ventilated. Following left thoracotomy at the fourth intercostal space, the LAD was ligated with a 6–0 suture. Successful MI was confirmed by ventricular blanching. The chest was closed and mice were extubated after recovery from anesthesia. Buprenorphine was administered for postoperative analgesia.

### Echocardiography

Cardiac function was assessed by transthoracic echocardiography using a Vevo 770 imaging system (VisualSonics). Mice were lightly anesthetized with isoflurane and maintained on a heated platform during imaging. Two-dimensional and M-mode images were acquired in the parasternal short-axis view at the level of the papillary muscles. Left-ventricular ejection fraction, fractional shortening and stroke volume were quantified from at least three consecutive cardiac cycles and averaged for analysis.

### Histology

Hearts were isolated and fixed in 4% paraformaldehyde in PBS overnight at 4 °C, then processed for paraffin embedding and sectioned in intervals. For trichrome staining following MI injury, hearts were sectioned from the ligation site to the apex. Every 50th section from each heart was selected for staining and quantification. H&E and Masson’s trichrome staining was performed according to the manufacturer’s protocol (Newcomer Supply). Scar size was quantified using six sections per heart from the ligation site to the apex. Fibrotic scar areas were analyzed using ImageJ and the average scar area for each heart was plotted.

Masson’s trichrome-stained heart sections were analyzed using ImageJ. Images were first subjected to color deconvolution using the built-in ‘Masson trichrome’ setting, which separates the blue collagen signal from the red myocardial tissue signal. For quantification of interstitial collagen deposition, high-magnification images were used for analysis. The collagen-separated images were thresholded to include visually identifiable blue collagen staining while excluding unstained or nonspecific background signal. Image analysis was performed blinded to the experimental groups and the same segmentation criteria were applied consistently to all samples. After thresholding, the collagen-positive area was automatically measured by ImageJ. Total myocardial tissue area was measured separately using the same approach, with the threshold adjusted to include the entire stained tissue area while excluding the surrounding background. Fibrosis was quantified as the collagen-positive area relative to the total myocardial tissue area. For scar quantification, complete heart cross-sections were analyzed using the same approach. The scar area was measured and expressed relative to the total cross-sectional area of the heart.

### Immunofluorescence

Cultured neonatal CFs were fixed with 4% PFA, permeabilized with 0.1% Triton X-100 and blocked with 1% BSA. Heart tissues were fixed overnight in 4% PFA, paraffin-embedded, sectioned at 5 µm and subjected to antigen retrieval (eBioscience, 00-4955-58). Cells or sections were incubated with primary antibodies overnight at 4 °C, followed by Alexa Fluor 488/555-conjugated secondary antibodies (Invitrogen). Samples were mounted with Fluoromount-G (SouthernBiotech, 0100-01) and imaged using a Nikon A1R confocal microscope or Keyence imaging system. Primary antibodies used were as follows: anti-PCM1 (Sigma, HPA023374, 1:200), anti-Ki67 (Abcam, ab279653, 1:200), anti-pH3 S10 (Invitrogen, MA5-15220, 1:200), anti-Ki67 (Abcam, ab15580, 1:200), anti-pH3 S10 (Millipore, 06-570, 1:200), anti-aurora B (Sigma, A5102, 1:50), anti-cTNT (Abcam, ab8295, 1:200), anti-BrdU (Abcam, ab1893, 1:200), anti-PDGFRα (Cell Signaling Technology, 3174, 1:500), anti-SDHB (Abcam, ab14714, 1:200), anti-Postn (Abcam, ab14041, 1:200), anti-Col3a1 (Abcam, ab184993, 1:200), anti-α-SMA (Invitrogen, 14-5698-82, 1:200) and anti-Vimentin (ABclonal, a19607, 1:200). DAPI was used for nuclear staining.

### WGA staining and CM cross-sectional area quantification

CM cross-sectional area was assessed by WGA and DAPI staining. After antigen retrieval (eBioscience, 00-4955-58), sections were permeabilized with 0.1% Triton X-100 and incubated with Alexa Fluor 488-conjugated WGA (Life Technologies, 1853992) for 1 h at room temperature. Nuclei were counterstained with DAPI and mounted with Fluoromount-G (SouthernBiotech, 0100-01). CM size was assessed in 3–5 biological replicates per group from ×200 images of the left ventricle, right ventricle and septum, with border and remote zones additionally analyzed after MI. WGA-defined CMs with clear circular cross-sections and central nuclei were quantified using ImageJ, with ≥900 CMs analyzed per sample.

### Targeted metabolomics

Adult mice underwent a 4-h fast before being killed by cervical dislocation. Hearts were collected, quickly rinsed in saline and then patted dry between two gauzes. The atria were removed and the tissue was flash-frozen in liquid nitrogen. After flash-freezing, the samples were placed into prechilled, labeled 2-ml snap-cap Eppendorf tubes and kept on dry ice for amino acid and organic acid measurements. For analysis of cardiac ventricular tissue, frozen mouse left ventricles were lyophilized overnight, powdered and homogenized in extraction solvent. Aliquots of homogenates were extracted in organic solvents for targeted liquid chromatography–mass spectrometry metabolomics (amino acids and organic acids) according to validated, optimized protocols in our previously published studies^62,63^. Specifically, to analyze amino acids, aliquots of mouse plasma and tissue homogenates were extracted with ice-cold methanol to precipitate protein before derivatization. To analyze organic acids, aliquots of tissue homogenates were derivatized and extracted into ethyl acetate by liquid–liquid extraction. These protocols use cold conditions and solvents to arrest cellular metabolism and maximize the stability and extraction recovery of metabolites. Each class of metabolites was separated with a unique HPLC method to optimize their chromatographic resolution and sensitivity. Quantitation of metabolites in each assay module was achieved using multiple reaction monitoring of calibration solutions and study samples on an Agilent 1290 Infinity UHPLC/6495 triple-quadrupole mass spectrometer^62,63^. Raw data were processed using Mass Hunter quantitative analysis software (Agilent). Calibration curves (*R*^2^ = 0.99 or greater) were either fitted with a linear or a quadratic curve with a 1/*x* or 1/*x*^2^ weighting.

### Electron microscopy

Hearts were washed with 0.2 M sodium cacodylate buffer and fixed in 2% glutaraldehyde and 4% paraformaldehyde for 24 h. Samples were processed at the University of Wisconsin-Madison Electron Microscopy Facility Core, including postfixation with 1% osmium tetroxide and 1% potassium ferrocyanide, graded ethanol/acetone dehydration and EMBed 812 resin infiltration and polymerization. Ultrathin sections (100 nm) were cut, mounted on copper grids, stained with Reynolds’ lead citrate and 8% uranyl acetate and imaged using an FEI CM120 transmission electron microscope with an AMT BioSprint12 camera.

### Western blotting

Myocardial or cell lysates were extracted in 8 M urea buffer containing protease and phosphatase inhibitors (Thermo Fisher, 78440) and protein concentrations were determined by BCA assay (Thermo Fisher, 23225). Proteins were separated on 4–20% SDS–PAGE gels, transferred to 0.22-µm PVDF membranes and incubated with primary antibodies overnight at 4 °C and LI-COR secondary antibodies for 2 h. Blots were imaged using an Odyssey FC system and quantified with ImageJ. Proteins of interest were targeted with the following antibodies: anti-Sdhb (Abcam, ab14714, 1:1,000), anti-Gapdh (Proteintech, 10494-1-AP, 1:1,000), anti-Vimentin (ABclonal, a19607, 1:1,000), anti-Col3a1 (Abcam, ab184993, 1:1,000), anti-H3K4me1 (Abcam, ab8895, 1:1,000), anti-H3K4me2 (Abcam, ab39141, 1:1,000), anti-H3K4me3 (Abcam, ab8580, 1:1,000), anti-H3K9me1 (Abcam, ab8896, 1:1,000), anti-H3K9me3 (Abcam, ab8898, 1:1,000), anti-H3K27me3 (Abcam, ab192985, 1:1,000), anti-H3K36me3 (Abcam, ab9050, 1:1,000), anti-H3K79me3 (Abcam, ab2621, 1:1,000), anti-H4K20me3 (Abcam, ab9053, 1:1,000), anti-H3K9ace (Sigma-Aldrich, 06-942, 1:1,000), anti-H3K14ace (Sigma-Aldrich, 07-353, 1:1,000), anti-H3K27ace (Abcam, ab4729, 1:1,000), anti-H3ace (Sigma-Aldrich, 06-599, 1:1,000) and anti-H3 (Abcam, ab1791, 1:1,000).

### NMCF isolation, culture and treatment

Neonatal CD-1 mouse ventricles were minced and digested with collagenase B (0.4 mg ml^−1^; Worthington, LS004176) and pancreatin (0.6 mg ml^−1^; Sigma, P3292). Cells were plated in DMEM containing 10% FBS and 1% penicillin–streptomycin and CFs were enriched by 2-h adhesion and expanded for two passages. At 80% confluence, CFs were serum-starved overnight and treated with human TGFβ1 (10 ng ml^−1^; R&D Systems, 100-21) and/or dimethyl malonate (5 mM; Sigma, 136411) for 48 h. For proliferation assays, CFs were incubated with EdU (10 µM; Invitrogen, C10338) for 24 h, fixed with 4% PFA and analyzed using the Click-iT EdU kit according to the manufacturer’s instructions. For migration assays, CFs (5 × 10^5^ per well) were seeded in six-well plates. After 24 h, monolayers were scratched with a 200-µl pipette tip, washed with PBS and cultured in serum-free medium with indicated treatments. Images were acquired at 0 and 48 h after scratching using a Leica DMi8 microscope and the migration ratio at both time points was quantified with ImageJ.

### hiPSC-derived CM differentiation, culture and treatment

hiPSCs derived from dermal fibroblasts and donated by the laboratory of J. Wu were maintained under feeder-free conditions and differentiated into CMs using a small-molecule-based cardiac differentiation protocol. In brief, iPSCs were cultured to confluency and cardiac differentiation was initiated by temporal modulation of Wnt signaling (48 h of 6 µM CHIR followed by 48 h of 2 µM C59, 24 h of medium with no insulin, 6 days with medium containing 4 mg ml^−1^ insulin and then culture in lipid-rich medium). Differentiated hiPSC-derived CMs were metabolically enriched (4 days of culture in medium containing 8 mM lactate and no glucose, days 15–19) and maintained in lipid-rich CM culture medium before treatment. For metabolite treatment experiments, hiPSC-derived CMs were replated and treated with malonate or selected metabolites, including α-ketoglutarate (Sigma, 13192-04-6), succinate (MedChemExpress, HY-Y0808), fumarate (Sigma, 624-49-7) and l-malate (Sigma, 617-55-0), at the indicated concentrations. Fresh treatment medium containing malonate or metabolites was replaced every 3 days throughout the experiment. EdU incorporation was performed 24 h before fixing the cells to label proliferating CMs. Cells were collected at the indicated time points after treatment and fixed using 4% PFA for 10 min for immunostaining. Cell were then permeabilized with 0.1% Triton X-100 in PBS for 10 min, blocked with blocking buffer for 1 h and incubated with primary antibody overnight at 4 °C, followed by secondary antibody staining for 1 h at room temperature. Cells were imaged at ×20 using an ImageXpress Micro (Molecular Devices). CMs were identified by ACTN2 staining and CM proliferation was quantified as the percentage of EdU^+^ ACTN2^+^ cells among total ACTN2^+^ CMs. Total CM number was quantified on the basis of ACTN2^+^ cells.

### Adult CM and nonmyocyte isolation

Adult CMs and nonmyocytes were isolated using separate digestion protocols. For CMs, mice were pretreated with 1% heparin (Thermo, U20K016) for 20 min and killed; hearts were cannulated and perfused on a Langendorff system (Harvard Apparatus) with prewarmed Tyrode’s solution followed by 300 U per ml collagenase II (Worthington, LS004176) at 37 °C for 5 min. Ventricles were dissociated in perfusion buffer containing 10% FBS and filtered through a 100-µm strainer; CMs were collected by sedimentation.

Because of insufficient collection of CFs from nonmyocytes from the Langendorff method, nonmyocytes for immunohistochemistry, western blot and CUT&RUN analysis were isolated from hearts using an alternate enzyme digestion method^64^. For nonmyocytes, hearts were perfused with cold Hanks’ balanced salt solution (HBSS) containing 0.8 mM CaCl_2_, minced and digested sequentially with collagenase II (500 U per ml, 30 min) and collagenase/dispase (1 mg ml^−1^; Roche, 20 min) at 37 °C. Digestion was stopped with 2% FBS in HBSS, CMs were pelleted at 30*g* for 2 min and the nonmyocyte-containing supernatant was filtered through a 40-µm strainer for downstream analyses.

### CF flow cytometry

Isolated nonmyocytes were stained with Ghost Dye violet 450 (1:1,000; Cytek, 13-0863-T100) in 2% FBS in HBSS for 15 min to discriminate viable and dead cells and then centrifuged (400*g*, 10 min). The supernatant was replaced with antibody stain, MEFSK4–PE (1:50; Miltenyi Biotech, 130-120-166) and ITGA7–Alexa Fluor 700 (1:20; Thermo Fisher Scientific, MA5-23607), in 2% FBS in HBSS for 30 min; panels were validated using no-stain and single-stain controls. Cells were washed three times (centrifugation, supernatant removal and resuspension in 2% FBS in HBSS) before final suspension in 2% FBS in HBSS with 2 mM EDTA. Sorting was performed on a BD FACSAria and analyzed with BD FACSDiva version 8.0.1 (Sanford Burnham Prebys Flow Cytometry core), gating CFs as MEFSK4^+^ (weakly expressed in mural cells)/ITGA7^−^ (mural cell marker)^22^. Cells were collected in pure FBS to improve viability and reduce adhesion to tubes.

### Statistical analysis of in vivo and in vitro experiments

All graphs are presented as the mean ± s.e.m. Statistical analysis was performed with Prism 11 (GraphPad Software). Data were assessed for consistency with the assumptions of the applied parametric tests. A two-tailed unpaired Student’s *t*-test was performed to determine the difference between the treatment group and control group. A one-way analysis of variance (ANOVA) was performed with the Tukey multiple-comparison test to determine the differences in group mean among treatment groups. A two-way ANOVA with Tukey’s post hoc test was used to determine the differences among the treatment groups at different time points. The level of significance was set at *P* < 0.05.

### snRNA-seq, snATAC-seq and CUT&RUN sequencing and analysis

#### snRNA & ATAC sequencing

Mouse hearts were collected below the ligation, snap-frozen in liquid nitrogen and then homogenized in a 4 °C cold room. Nuclear isolation used the Chromium nucleus isolation kit (10x Genomics, PN-1000493); nuclei from four mice were pooled per sample condition, with 10,000 nuclei per sample used to generate each library. RNA and ATAC libraries were constructed per manufacturer protocol for the Chromium Next GEM single-cell multiome ATAC + gene expression kit (10x Genomics, PN-1000283) by the University of Wisconsin-Madison Gene Expression Center, with quality control using Qubit (1× high-sensitivity DNA) and Agilent 2100 (high-sensitivity DNA kit). Libraries were sequenced on a NovaSeq 6000 (NovaSeq S2, 2 × 50 bp) across two lanes per sample; all processing occurred on the same day to avoid batch effects.

#### snRNA-seq quality control and filtering

Data were processed with Cell Ranger ARC 3.0 and aligned to GRCm38/mm10. Ambient RNA was removed using CellBender 0.3.0 ‘remove-background’ (--total-droplets-included=25,000, --low-count-threshold=15, --epochs=200)^65^, with expected-cells set to 1.5× the Cell Ranger ARC-estimated nuclei count to minimize barcode loss. Low-quality nuclei were excluded using fixed cutoffs (RNA unique molecular identifier (UMI) count < 200, RNA gene count < 150, mitochondrial UMI > 5%, ATAC UMI count < 50 or ATAC gene count < 50), followed by dynamic filtering to remove nuclei with RNA UMI counts below the 2.5th or above the 97.5th percentiles. Doublets were identified per sample using Scrublet (expected doublet rate = 0.8% per 1,000 recovered cells, per 10x Genomics v3 guidance), with the call_doublets_threshold set by visualizing singlet and doublet peak separation^66^. Downstream analysis used Seurat 5.0.3 and Signac 1.12.0 in R 4.3.3 (refs. ^67,68^). UMI matrices were normalized by SCTransform, regressing out mitochondrial and ribosomal percentages. Following clustering, clusters with median RNA UMI count < 1,000 or expressing markers from multiple distinct cell types were removed at both global and cell-type-specific levels (Extended Data Fig. 1). ATAC data quality was confirmed by requiring TSS enrichment score > 1 and nucleosome signal < 2 for all nuclei.

### ATAC peak calling

Peaks were called using Signac’s callPeaks function (MACS2), merged into a unified consensus peak-by-nucleus matrix and filtered to exclude peaks < 20 bp or peaks > 10,000 bp^69^. Transcription factor motif accessibility was quantified using Signac’s runChromVAR, computing per-cell deviation scores relative to a background of matched peaks.

### Library integration and batch alignment

RNA modality integration used Seurat’s IntegrateLayers with the Harmony algorithm; ATAC integration used RunHarmony, projecting cells into a shared embedding grouped by cell type^70^.

### Principal component analysis and unsupervised clustering

After integration, data were scaled and principal component analysis (PCA) was run on 5,000 highly variable genes, yielding 50 principal components for global clustering (30–50 principal components for subcluster-level analysis in both modalities, per elbow plot). Clusters were identified using Seurat’s FindNeighbors and FindClusters (*k*-nearest neighbor graph-based clustering; resolution 0.1–1), optimized for clear uniform manifold approximation and projection (UMAP) separation and >20 significantly differentially expressed genes per subcluster. This process was repeated after cell type subsetting (using cell-type-specific highly variable genes) and again after remerging into the final global dataset.

### Joint snRNA and ATAC embedding

RNA and ATAC modalities were integrated into a joint low-dimensional embedding using Seurat’s FindMultiModalNeighbors, constructing a weighted nearest neighbor graph for UMAP dimensionality reduction.

### Differentially expressed gene analysis

Marker genes were identified using FindMarkers (pairwise) and FindAllMarkers (multicluster) with the MAST (model-based analysis of single-cell transcriptomics) likelihood ratio test optimized for single-cell transcriptomic analysis^71^. Significance thresholds for two-sided analysis were as follows: Benjamini–Hochberg-adjusted (for multiple comparisons) *P* < 0.05, log_2_FC  > 0.25, min.pct > 0.05.

### GO and KEGG enrichment analysis

GO (biological process) and KEGG analysis used clusterProfiler 4.10.1 (ref. ^72^), filtered at false discovery rate *q* < 0.05 and Benjamini–Hochberg-adjusted *P* < 0.05.

### ATAC peak and motif analysis

ATAC peaks near genes of interest were visualized using Signac’s CoveragePlot, with tracks normalized by Signac’s default group-based scaling (accounting for nucleus number and mean sequencing depth per group). Differentially accessible peaks were identified using FindMarkers/FindAllMarkers (two-tailed Wilcoxon rank-sum test), with the nearest genes assigned using CHIPSeeker 1.38 (ref. ^73^). Motif analysis used the JASPAR2020 database; motif position weight matrices were added using Signac’s AddMotifs and RunChromVar was used to identify motif-associated chromatin accessibility deviations. Differentially accessible motifs were identified using FindMarkers and FindAllMarkers (two-tailed Wilcoxon rank-sum test); significance thresholds were as follows: Benjamini–Hochberg-adjusted *P* < 0.05, log_2_FC > 0.25, min.pct > 0.05.

### Cell fixation and cryopreservation for CUT&RUN

Isolated CMs and CFs were centrifuged (400*g*, 10 min) and briefly fixed in 0.1% PFA for 2 min per the manufacturer’s CUT&RUN sample preparation protocol (Cell Signaling Technology) to preserve chromatin architecture during cryopreservation. Fixation was quenched with 1.25 M glycine and cells were counted, recentrifuged (400*g*, 10 min) and resuspended in 2% FBS. For cryopreservation, cells were suspended in 100 µl per 1 × 10^5^ cells of cryopreservation solution (10% DMSO and 90% FBS) and frozen at −80 °C; cells were thawed quickly at 37 °C before CUT&RUN.

### CUT&RUN sequencing

CUT&RUN was performed using the CUT&RUN assay kit (86652S, Cell Signaling Technology) per the manufacturer’s instructions, modified for our sample types, cell numbers and antibodies, using ~250,000 cells per condition (including an H3K4me3 positive control and rabbit IgG negative control per biological group). Cells were bound to activated concanavalin A beads and incubated overnight at 4 °C with primary antibodies, followed by pAG-MNase digestion, calcium-activated fragment release and crosslink reversal (SDS and proteinase K, 65 °C). DNA was purified (Qiagen MinElute PCR purification kit, 28006) and quantified by Qubit; fragment size was assessed using an Agilent 2200 TapeStation (high-sensitivity D1000 ScreenTape). Libraries were prepared using the NEBNext Ultra II DNA library prep kit for Illumina (New England Biolabs, E7645L) with NEBNext multiplex oligos for Illumina (dual index primers set 1; New England Biolabs, E7600S), cleaned up with AMPure XP (Beckman Coulter, A63881) and sequenced on the Element Biosciences AVITI platform (2 × 75 bp, paired-end reads, ~10 million reads per sample). Antibodies used were as follows: rabbit anti-H3K4me3 (Cell Signaling Technology, 9751, 1:50), IgG XP isotype control (Cell Signaling Technology, 66362, 1:20) and rabbit anti-H3K27me3 (Cell Signaling Technology, 9733, 1:50). All conditions were performed with two biological replicates (*n* = 2).

### CUT&RUN data processing and analysis

Raw data were processed using nf-core/cutandrun pipeline 3.2.2 through Singularity, with adaptor trimming (Trim Galore), alignment to GRCm38/mm10 (Bowtie2, end-to-end mode) and duplicate removal (Picard MarkDuplicates). Signal tracks (bigWig) were generated with deepTools bamCoverage (counts per million normalization, 1-bp bin size); peak calling used both SEACR31 and MACS2 through the pipeline’s --peakcaller ‘seacr,MACS2’ parameter. SEACR 1.3 peaks were called at a threshold of 0.01 (stringent mode) for H3K4me3 and 0.05 (stringent mode) for H3K27me3. Differential histone modification signal was assessed using DESeq2 1.42, with reads from all eight BAM files (4 conditions × 2 replicates) per cell type and antibody combination counted within consensus peaks using featureCounts (Rsubread 2.14). Significant differential peaks were annotated using ChIPseeker 1.38 (ref. ^73^) with the UCSC mm10 RefSeq gene annotation (TxDb.Mmusculus.UCSC.mm10.knownGene).

### snRNA-seq cross-validation analysis

Given the limited statistical power of genome-wide differential chromatin analysis with *n* = 2 biological replicates, a targeted cross-validation approach compared the CUT&RUN signal at snRNA-seq-defined differentially expressed genes (identified through MAST, as above) against matched background genes drawn from genes clearly nondifferentially expressed in the snRNA-seq data (randomly sampled, fixed seed for reproducibility). For each test gene, up to five background genes were selected, matching simultaneously on (1) expression breadth (Seurat’s pct.1 metric, within ±0.20 of the test gene) and (2) gene body length (from Ensembl mmusculus_gene_ensembl through biomaRt), using length-matching windows set per histone mark to reflect their respective accessibility domains: ±25% of test gene length for H3K4me3, ±100% for H3K27me3. CUT&RUN log_2_FC values were compared between test and matched background gene sets using a two-sided Wilcoxon rank-sum test.

### Reporting summary

Further information on research design is available in the Nature Portfolio Reporting Summary linked to this article.

## Supplementary information

Supplementary Information Supplementary Figs. 1–5 and Supplementary Data 1 and 2.

Reporting Summary

Supplementary Tables Supplementary Table 1: Single-nucleus differentially expressed gene/accessible peak values for global population in Extended Data Fig. 1. Supplementary Table 2: Single-nucleus differentially expressed gene values for individual cell types by treatment condition in Extended Data Fig. 2 and Supplementary Fig. 1. Supplementary Table 3: Single-nucleus GO values for individual cell types in Figs. 2 and 3, Extended Data Figs. 2–4 and Supplementary Fig. 1. Supplementary Table 4: Single-nucleus KEGG values for individual cell types in Extended Data Figs. 2–4. Supplementary Table 5: Single-nucleus differentially expressed genes for individual cell types by subpopulation in Figs. 2 and 3, Extended Data Figs. 3 and 4 and Supplementary Fig. 2. Supplementary Table 6: Single-nucleus differentially accessible peak values for individual cell types in Figs. 2 and 3. Supplementary Table 7: Single-nucleus differentially accessible motif values for individual cell types in Fig. 2 and Supplementary Fig. 2. Supplementary Table 8: CUT&RUN differential peak values and GO values for Fig. 8.

## Source data

Source Data Fig. 4 Statistical source data.

Source Data Fig. 4 Unprocessed western blots.

Source Data Fig. 5 Statistical source data.

Source Data Fig. 5 Unprocessed western blots.

Source Data Fig. 6 Statistical source data.

Source Data Fig. 6 Unprocessed western blots.

Source Data Fig. 7 Statistical source data.

Source Data Extended Data Fig. 5 Statistical source data.

Source Data Extended Data Fig. 5 Unprocessed western blots.

Source Data Extended Data Fig. 7 Statistical source data.

Source Data Extended Data Fig. 8 Statistical source data.

Source Data Extended Data Fig. 8 Unprocessed western blots.

## Data Availability

snRNA-seq and snATAC-seq data are available from the NCBI Gene Expression Omnibus (GEO) under accession number GSE299884. All data generated and analyzed during this study are included in the manuscript and supplementary files.

## Code availability

Code for reproducing all bioinformatics analyses is available from Zenodo (https://doi.org/10.5281/zenodo.21987923)^74^ and GitHub (https://github.com/duttall/2026-SDH-inhibition-snMultiome-CUTnRUN).
