# Supplementary material for: A metabolic–epigenetic switch governs multicellular cardiac repair following succinate dehydrogenase inhibition
Source: Nat Cardiovasc Res. 2026 Sep 28;5(10):1034–54. doi: 10.1038/s44161-026-00881-9 (PMC13658034; doi:10.1038/s44161-026-00881-9)

Extended Data Figure 8. G

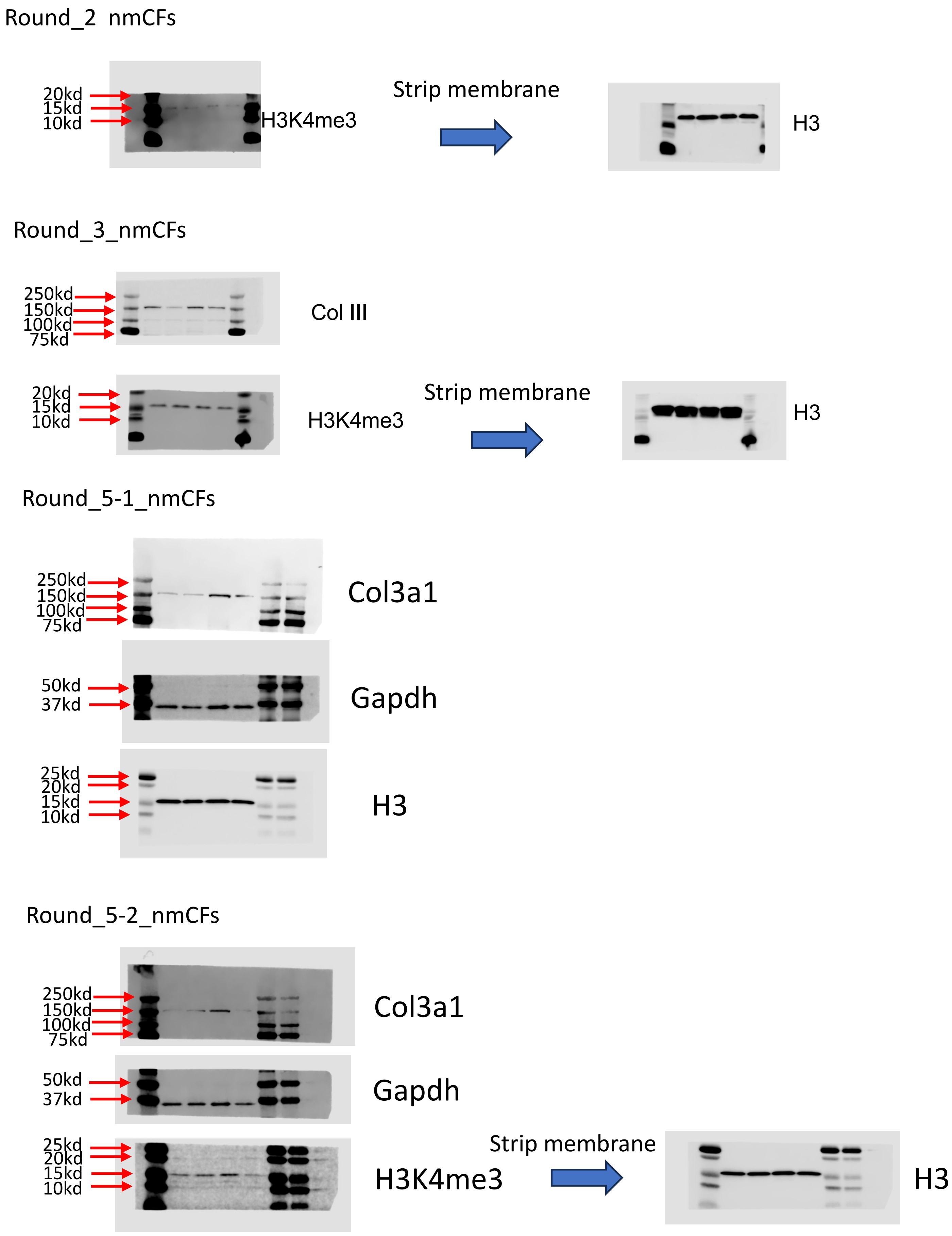

Supplement: Supplementary file 2 [file 44161_2026_881_MOESM15_ESM.pdf]
