# Supplementary material for: A metabolic–epigenetic switch governs multicellular cardiac repair following succinate dehydrogenase inhibition
Source: Nat Cardiovasc Res. 2026 Sep 28;5(10):1034–54. doi: 10.1038/s44161-026-00881-9 (PMC13658034; doi:10.1038/s44161-026-00881-9)
Supplement: Supplementary file 3 [file 44161_2026_881_MOESM1_ESM.pdf]

nature cardiovascular research

Supplementary information
https://doi.org/10.1038/s44161-026-00881-9

A metabolic–epigenetic switch governs multicellular cardiac repair following succinate dehydrogenase inhibition

In the format provided by the authors and unedited

Supplementary information

Supplementary Figures	Supplementary Figure 1. Malonate treatment alters the transcriptional profiles of CMs in uninjured mouse hearts.
	Supplementary Figure 2. CM subpopulations demonstrate distinct motif accessibility enrichment.
	Supplementary Figure 3. SDHB protein level in liver tissue from SDHBcko and control mice.
	Supplementary Figure 4. FACS gating strategy for isolating cardiac fibroblasts.
	Supplementary Figure 5. Correlation and peak distribution of CUT&RUN replicates.
Supplementary Data 1	Supplementary Figure 3_Unprocessed western blot
Supplementary Data 2	Supplementary Figure 3_statistical source data

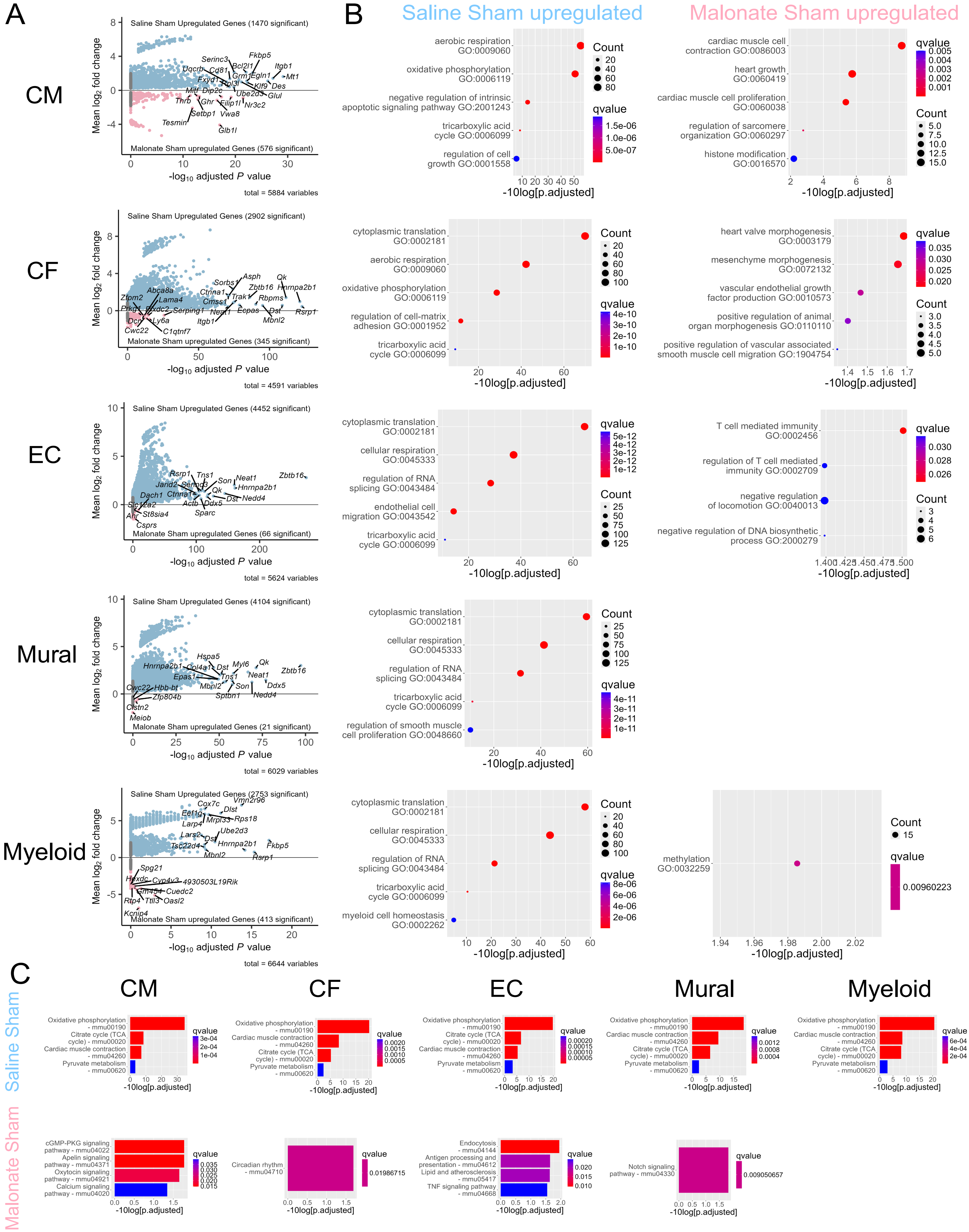
A
B
Saline Sham upregulated
Malonate Sham upregulated
CM
Saline Sham Upregulated Genes (1470 significant)
Malonate Sham upregulated Genes (576 significant)
Mean log2 fold change
-log10 adjusted P value
total = 5884 variables
aerobic respiration GO:0009060
oxidative phosphorylation GO:0006119
negative regulation of intrinsic apoptotic signaling pathway GO:2001243
tricarboxylic acid cycle GO:0006099
regulation of cell growth GO:0001558
cardiac muscle cell contraction GO:0086003
heart growth GO:0060419
cardiac muscle cell proliferation GO:0060038
regulation of sarcomere organization GO:0060297
histone modification GO:0016570
CF
Saline Sham Upregulated Genes (2902 significant)
Malonate Sham upregulated Genes (345 significant)
total = 4591 variables
cytoplasmic translation GO:0002181
aerobic respiration GO:0009060
oxidative phosphorylation GO:0006119
regulation of cell-matrix adhesion GO:0001952
tricarboxylic acid cycle GO:0006099
heart valve morphogenesis GO:0003179
mesenchyme morphogenesis GO:0072132
vascular endothelial growth factor production GO:0010573
positive regulation of animal organ morphogenesis GO:0110110
positive regulation of vascular associated smooth muscle cell migration GO:1904754
EC
Saline Sham Upregulated Genes (4452 significant)
Malonate Sham upregulated Genes (66 significant)
total = 5624 variables
cytoplasmic translation GO:0002181
cellular respiration GO:0045333
regulation of RNA splicing GO:0043484
endothelial cell migration GO:0043542
tricarboxylic acid cycle GO:0006099
T cell mediated immunity GO:0002456
regulation of T cell mediated immunity GO:0002709
negative regulation of locomotion GO:0040013
negative regulation of DNA biosynthetic process GO:2000279
Mural
Saline Sham Upregulated Genes (4104 significant)
Malonate Sham upregulated Genes (21 significant)
total = 6029 variables
cytoplasmic translation GO:0002181
cellular respiration GO:0045333
regulation of RNA splicing GO:0043484
tricarboxylic acid cycle GO:0006099
regulation of smooth muscle cell proliferation GO:0048660
Myeloid
Saline Sham Upregulated Genes (2753 significant)
Malonate Sham upregulated Genes (413 significant)
total = 6644 variables
cytoplasmic translation GO:0002181
cellular respiration GO:0045333
regulation of RNA splicing GO:0043484
tricarboxylic acid cycle GO:0006099
myeloid cell homeostasis GO:0002262
methylation GO:0032259
Count
qvalue
-10log[p.adjusted]
C
CM
CF
EC
Mural
Myeloid
Saline Sham
Malonate Sham
Oxidative phosphorylation - mmu00190
Citrate cycle (TCA cycle) - mmu00020
Cardiac muscle contraction - mmu04260
Pyruvate metabolism - mmu00620
cGMP-PKG signaling pathway - mmu04022
Apelin signaling pathway - mmu04371
Oxytocin signaling pathway - mmu04921
Calcium signaling pathway - mmu04020
Circadian rhythm - mmu04710
Endocytosis - mmu04144
Antigen processing and presentation - mmu04612
Lipid and atherosclerosis - mmu05417
TNF signaling pathway - mmu04668
Notch signaling pathway - mmu04330

Supplementary Figure 1. Malonate treatment alters the transcriptional profiles of CMs in uninjured mouse hearts.

a, Volcano plots comparing differentially expressed genes between pseudobulked Saline Sham and Malonate Sham CMs, CFs, ECs, Mural, and Myes, displaying an increase in terms associated with aerobic metabolism in all Saline Sham populations, and proliferation and growth in the Malonate Sham populations. differentially expressed genes were generated via MAST and p values were two-sided and Benjamini-Hochberg adjusted for multiple comparisons. **b**, One-sided GO plots were then generated from significant differentially expressed genes from the Saline Sham vs Malonate Sham analysis, all p values are Benjamini-Hochberg adjusted. **c**, One-sided KEGG Pathway enrichment plots generated from the same differentially expressed genes comparing Saline Sham to Malonate Sham, all p values are Benjamini-Hochberg adjusted. All adjusted p values are significant at $P < 0.05$.

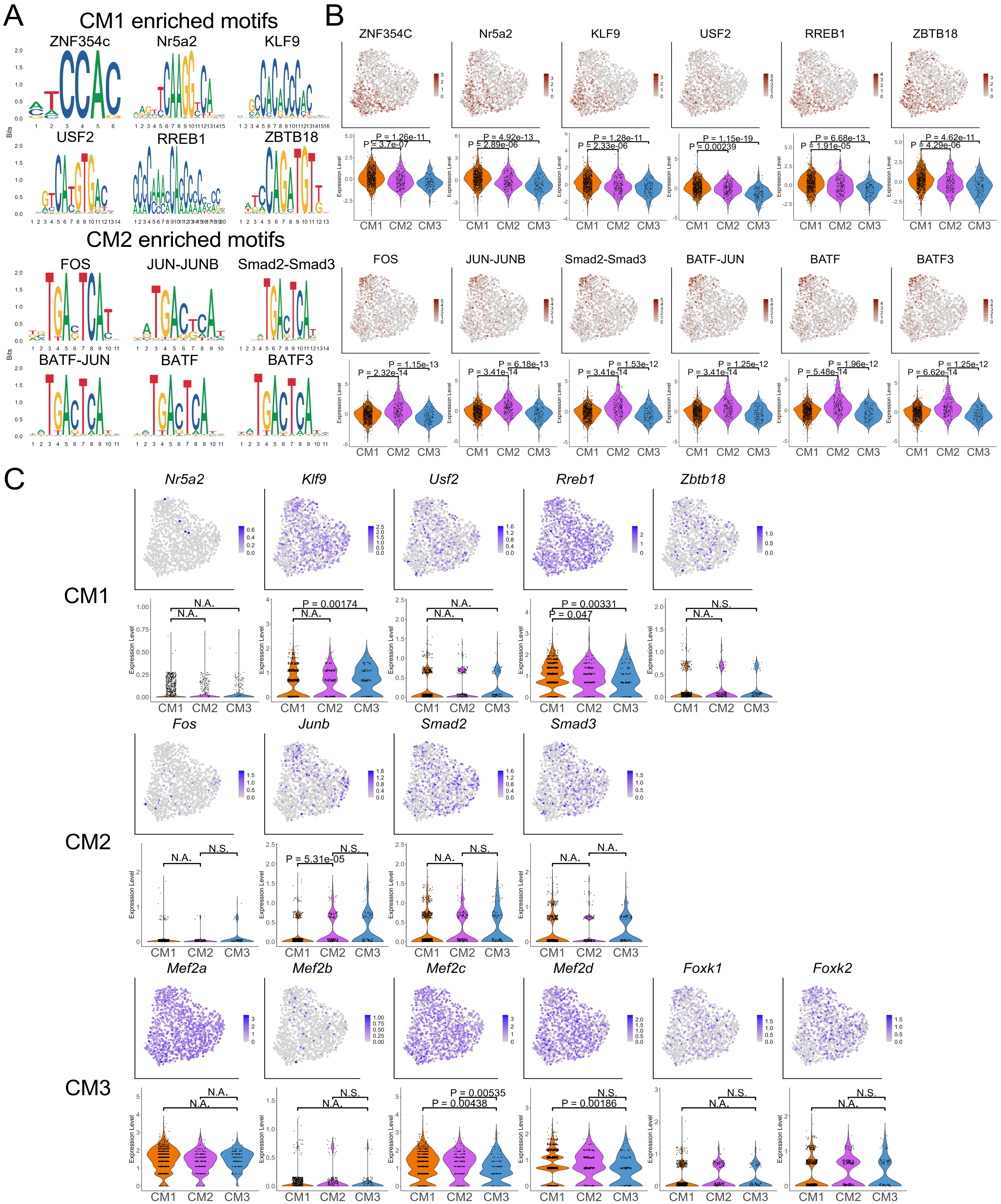
A
CM1 enriched motifs
ZNF354c
Nr5a2
KLF9
USF2
RREB1
ZBTB18
CM2 enriched motifs
FOS
JUN-JUNB
Smad2-Smad3
BATF-JUN
BATF
BATF3
Bits
B
ZNF354C
Nr5a2
KLF9
USF2
RREB1
ZBTB18
P = 1.26e-11
P = 3.7e-07
P = 4.92e-13
P = 2.89e-06
P = 1.28e-11
P = 2.33e-06
P = 1.15e-19
P = 0.00239
P = 6.68e-13
P = 1.91e-05
P = 4.62e-11
P = 4.29e-06
FOS
JUN-JUNB
Smad2-Smad3
BATF-JUN
BATF
BATF3
P = 1.15e-13
P = 2.32e-14
P = 6.18e-13
P = 3.41e-14
P = 1.53e-12
P = 3.41e-14
P = 1.25e-12
P = 3.41e-14
P = 1.96e-12
P = 5.48e-14
P = 1.25e-12
P = 6.62e-14
Expression Level
CM1 CM2 CM3
C
CM1
Nr5a2
Klf9
Usf2
Rreb1
Zbtb18
N.A.
P = 0.00174
P = 0.00331
P = 0.047
N.S.
CM2
Fos
Junb
Smad2
Smad3
P = 5.31e-05
CM3
Mef2a
Mef2b
Mef2c
Mef2d
Foxk1
Foxk2
P = 0.00535
P = 0.00438
P = 0.00186

Supplementary Figure 2. CM subpopulations demonstrate distinct motif accessibility enrichment.
a, b, (**a**) Motif enrichment plots for six of the most significant motifs with increased accessibility in CM1 (top;p values ordered: CM1 vs. CM2, CM1 vs. CM3; ZNF354C: P = 3.7x10^-07, P = 1.26x10^-11; Nr5a2: P = 2.89x10^-06, P = 4.92x10^-13; KLF9: P = 2.33x10^-06, P = 1.28x10^-11; USF2: P = 0.00239, P = 1.15x10^-19; RREB1: P = 1.91x10^-05, P = 6.68x10^-13; ZBTB18: P = 4.29x10^-06, P = 4.62x10^-11) and CM2 (bottom;p values ordered: CM2 vs. CM1, CM2 vs. CM3; FOS: P = 2.32x10^-14, P = 1.15x10^-13; JUN-JUNB: P = 3.41x10^-14, P = 6.18x10^-13, Smad2-Smad3: P = 3.41x10^-14, P = 1.53x10^-12; BATF-JUN: P = 3.41x10^-14, P = 1.25x10^-12; BATF: P = 5.48x10^-14, P = 1.96x10^-12; BATF3: P = 6.62x10^-14, P = 1.25x10^-12) and (**b**) their corresponding motif feature UMAPs (top) and violin plot, all p values are Benjamini-Hochberg adjusted (two-tailed Wilcoxon rank sum). **c**, Gene expression UMAP and violin plots for the transcription factors associated with the enriched motifs in CM1 (p values ordered: CM1 vs. CM2, CM1 vs. CM3; *Nr5a2*: N.A., N.A.; *Klf9*: P = N.A., P = 0.00174; *Usf2*: N.A., N.A.; *Rreb1*: P = 0.04703, P = 0.00331; *Zbtb18*: N.A., P = 0.4181; no data for *Znf354c*), CM2 (p values ordered: CM2 vs. CM1, CM2 vs. CM3; *Fos*: N.A., 0.1771; *Junb*: P = 5.31 × 10^-05, P = 0.6551; *Smad2*: N.A., P = 0.4603; *Smad3*: N.A., N.A.; no data for *Batf* and *Batf3*), and CM3 (p values ordered: CM3 vs. CM1, CM3 vs. CM2; *Mef2a*: N.A., N.A.; *Mef2b*: N.A., P = 0.4723; *Mef2c*: P = 0.00535; P = 0.00438; *Mef2d*: P = 0.00186; P = 0.1431; *Foxk1*: N.A., P = 0.5527; *Foxk2*: N.A., P = 0.1647) (no data for *Znf354c*, *Batf*, and *Batf3*). These statistics are Benjamini-Hochberg two-sided adjusted p values (MAST). All adjusted p-values are reported as exact values where P < 0.05 (significant); as 'N.S.' where P ≥ 0.05 (tested, not significant); or as 'N.A.' where the gene/peak did not meet minimum thresholds for inclusion in differential testing (log2FC > 0.25, min.pct > 0.05) and therefore has no associated p-value.

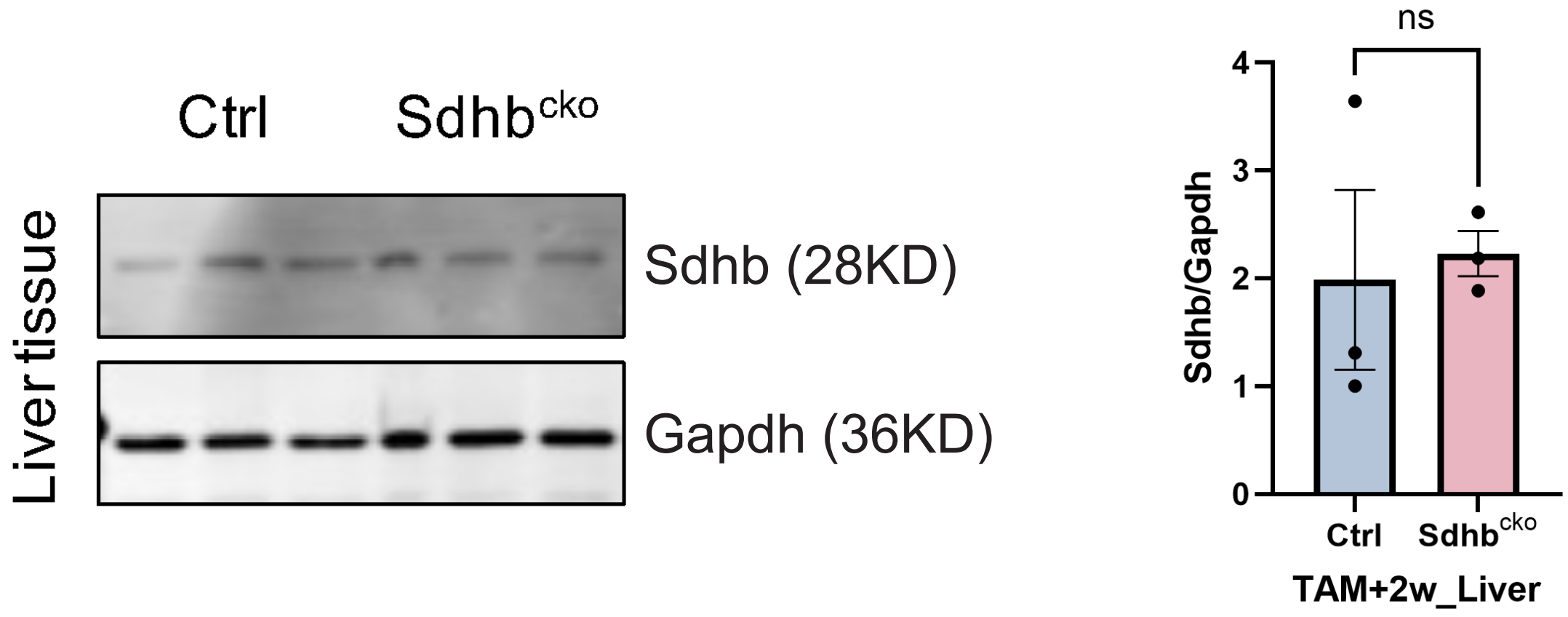

Ctrl
Sdhb^{cko}
Liver tissue
Sdhb (28KD)
Gapdh (36KD)
ns
Sdhb/Gapdh
0
1
2
3
4
Ctrl
Sdhb^{cko}
TAM+2w_Liver

Supplementary Figure 3. SDHB protein level in liver tissue from *SDHB*^{cko} and control mice. Representative western blots (left) and quantification (right) showing SDHB protein levels in liver tissue from control and Sdhbcko mice at the indicated time points (P = 0.7905). GAPDH was used as a loading control.

Single cell suspension of ventricle

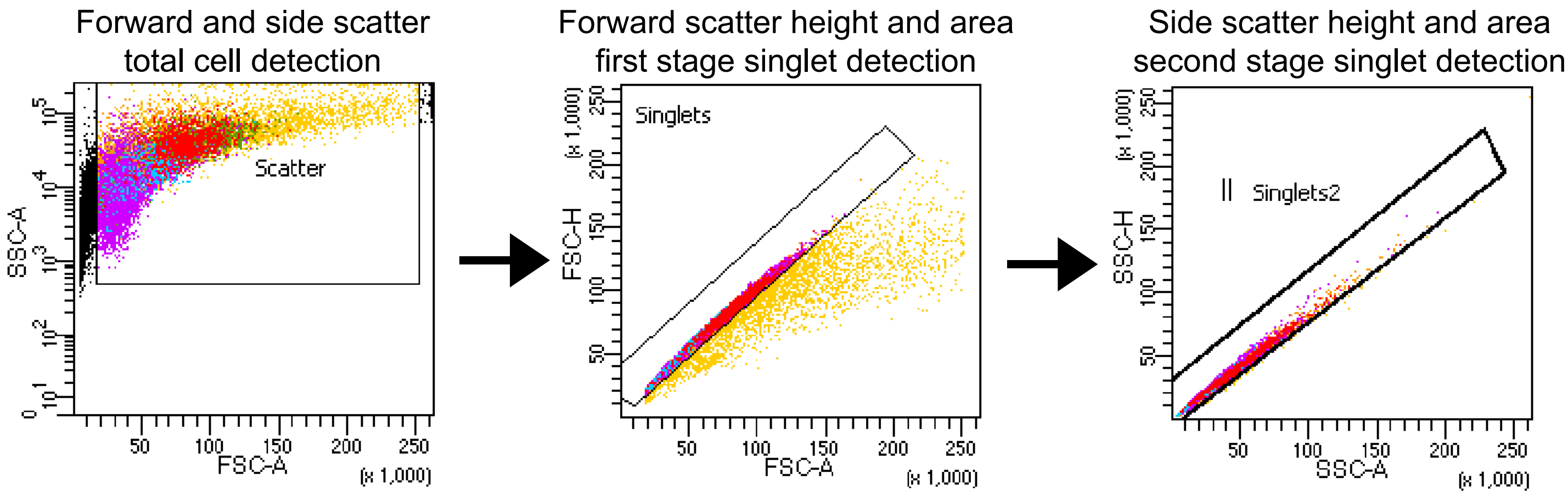

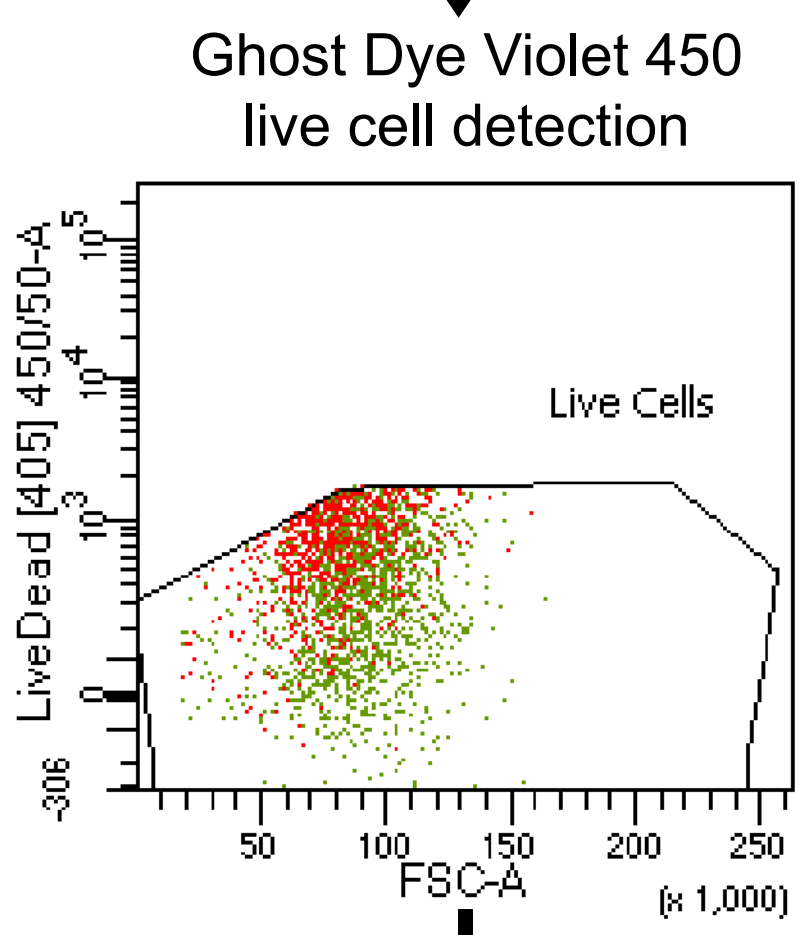

Tube: Cre			
Population	#Events	%Parent	%Total
All Events	30,000	####	100.0
Scatter	22,510	75.0	75.0
Singlets	19,355	86.0	64.5
Singlets2	19,294	99.7	64.3
Live Cells	16,187	83.9	54.0
High Mefska4+	1,236	7.6	4.1
Low Mefska4+	376	2.3	1.3
P1	1,135	7.0	3.8

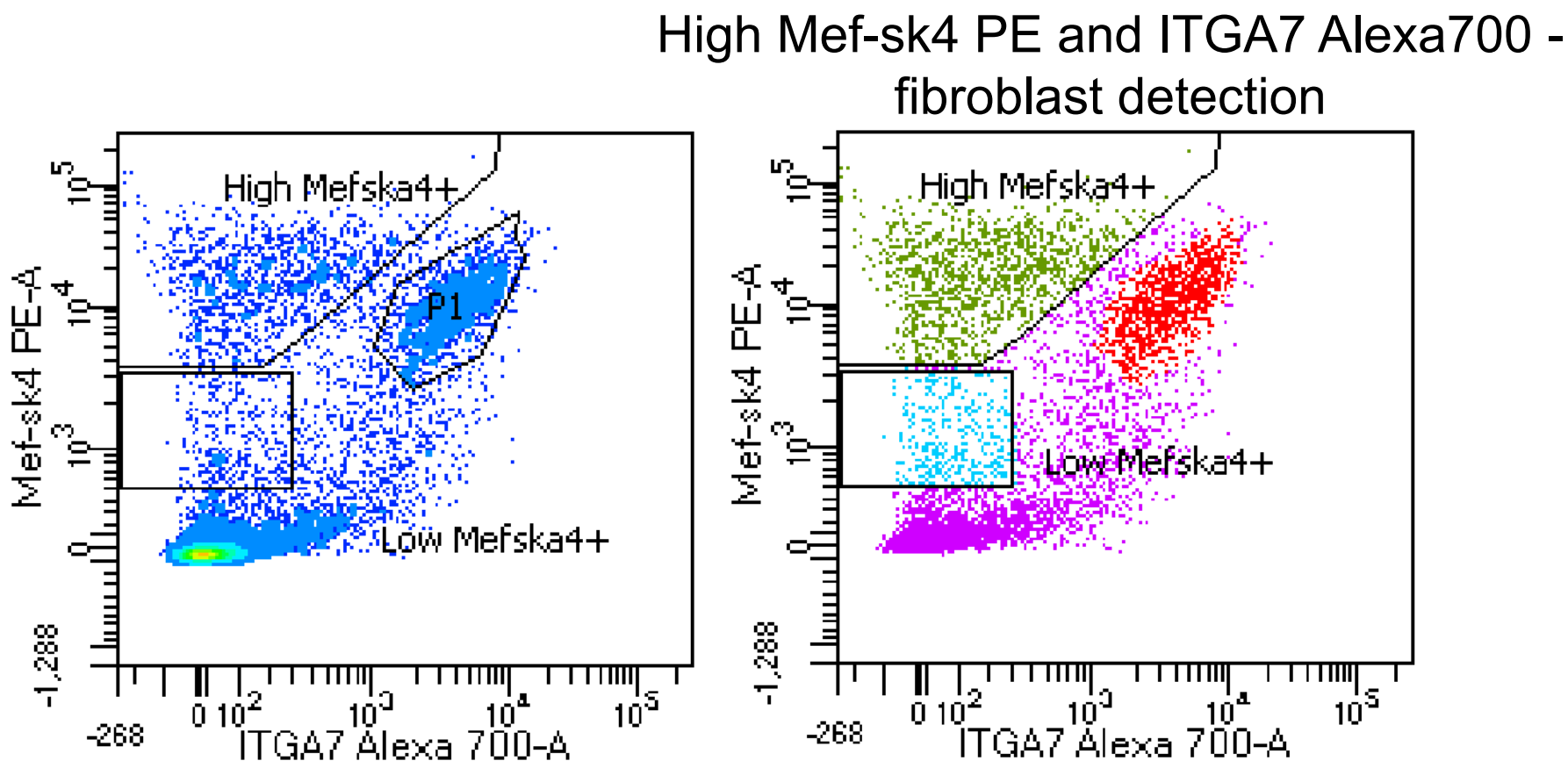

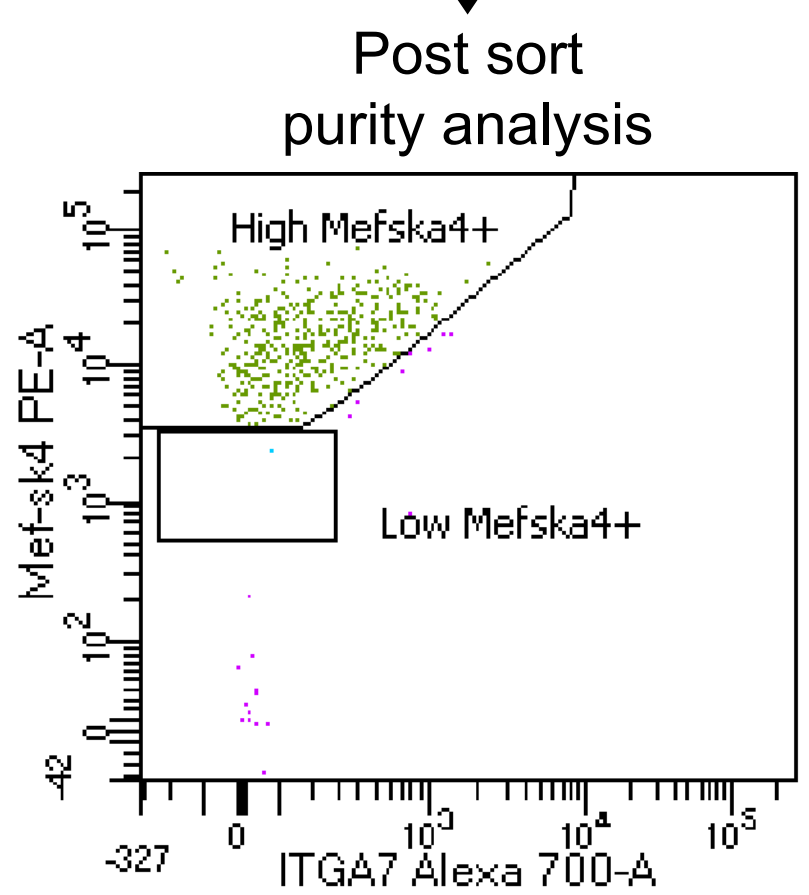

Tube: Cre High post sort			
Population	#Events	%Parent	%Total
All Events	549	####	100.0
Scatter	535	97.4	97.4
Singlets	493	92.1	89.8
Singlets2	493	100.0	89.8
Live Cells	491	99.6	89.4
High Mefska4+	467	95.1	85.1
Low Mefska4+	1	0.2	0.2

Supplementary Figure 4. FACS gating strategy for isolating cardiac fibroblasts.
A flow chart displaying the gating strategy used to isolate CFs from a single cell suspension derived from mouse ventricular tissue.

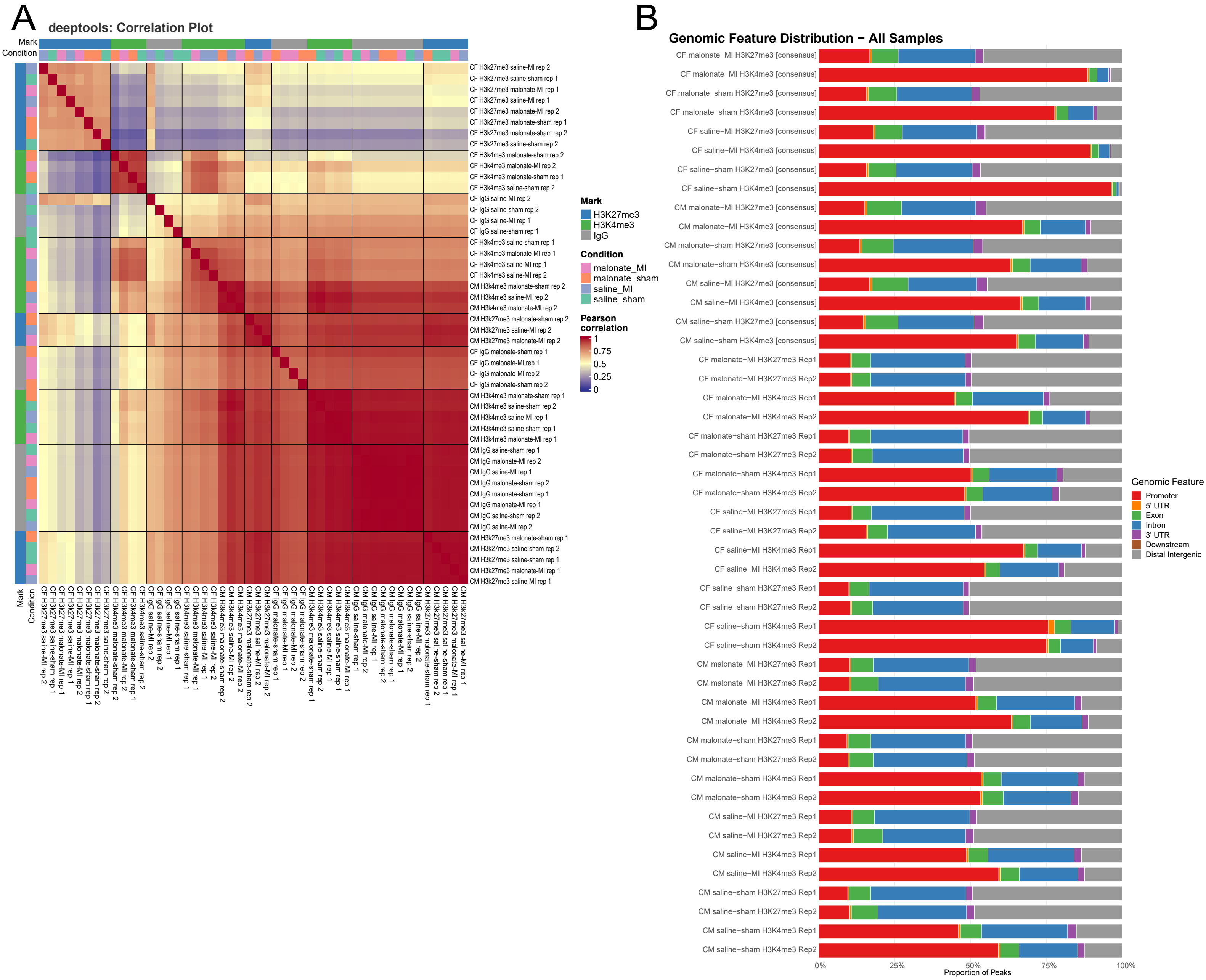
A
deeptools: Correlation Plot
Mark
Condition
CF H3k27me3 saline-MI rep 2
CF H3k27me3 saline-sham rep 1
CF H3k27me3 malonate-MI rep 1
CF H3k27me3 saline-MI rep 1
CF H3k27me3 malonate-MI rep 2
CF H3k27me3 malonate-sham rep 1
CF H3k27me3 malonate-sham rep 2
CF H3k27me3 saline-sham rep 2
CF H3k4me3 malonate-sham rep 2
CF H3k4me3 malonate-MI rep 2
CF H3k4me3 malonate-sham rep 1
CF H3k4me3 saline-sham rep 2
CF IgG saline-MI rep 2
CF IgG saline-sham rep 2
CF IgG saline-MI rep 1
CF IgG saline-sham rep 1
CF H3k4me3 saline-sham rep 1
CF H3k4me3 malonate-MI rep 1
CF H3k4me3 saline-MI rep 1
CF H3k4me3 saline-MI rep 2
CM H3k4me3 malonate-sham rep 2
CM H3k4me3 saline-MI rep 2
CM H3k4me3 malonate-MI rep 2
CM H3k27me3 malonate-sham rep 2
CM H3k27me3 saline-MI rep 2
CM H3k27me3 malonate-MI rep 2
CF IgG malonate-sham rep 1
CF IgG malonate-MI rep 1
CF IgG malonate-MI rep 2
CF IgG malonate-sham rep 2
CM H3k4me3 malonate-sham rep 1
CM H3k4me3 saline-sham rep 2
CM H3k4me3 saline-MI rep 1
CM H3k4me3 saline-sham rep 1
CM H3k4me3 malonate-MI rep 1
CM IgG saline-sham rep 1
CM IgG malonate-MI rep 2
CM IgG saline-MI rep 1
CM IgG malonate-sham rep 2
CM IgG malonate-sham rep 1
CM IgG malonate-MI rep 1
CM IgG saline-sham rep 2
CM IgG saline-MI rep 2
CM H3k27me3 malonate-sham rep 1
CM H3k27me3 saline-sham rep 2
CM H3k27me3 saline-sham rep 1
CM H3k27me3 malonate-MI rep 1
CM H3k27me3 saline-MI rep 1
Mark
H3K27me3
H3K4me3
IgG
Condition
malonate_MI
malonate_sham
saline_MI
saline_sham
Pearson correlation
1
0.75
0.5
0.25
0
B
Genomic Feature Distribution – All Samples
CF malonate–MI H3K27me3 [consensus]
CF malonate–MI H3K4me3 [consensus]
CF malonate–sham H3K27me3 [consensus]
CF malonate–sham H3K4me3 [consensus]
CF saline–MI H3K27me3 [consensus]
CF saline–MI H3K4me3 [consensus]
CF saline–sham H3K27me3 [consensus]
CF saline–sham H3K4me3 [consensus]
CM malonate–MI H3K27me3 [consensus]
CM malonate–MI H3K4me3 [consensus]
CM malonate–sham H3K27me3 [consensus]
CM malonate–sham H3K4me3 [consensus]
CM saline–MI H3K27me3 [consensus]
CM saline–MI H3K4me3 [consensus]
CM saline–sham H3K27me3 [consensus]
CM saline–sham H3K4me3 [consensus]
CF malonate–MI H3K27me3 Rep1
CF malonate–MI H3K27me3 Rep2
CF malonate–MI H3K4me3 Rep1
CF malonate–MI H3K4me3 Rep2
CF malonate–sham H3K27me3 Rep1
CF malonate–sham H3K27me3 Rep2
CF malonate–sham H3K4me3 Rep1
CF malonate–sham H3K4me3 Rep2
CF saline–MI H3K27me3 Rep1
CF saline–MI H3K27me3 Rep2
CF saline–MI H3K4me3 Rep1
CF saline–MI H3K4me3 Rep2
CF saline–sham H3K27me3 Rep1
CF saline–sham H3K27me3 Rep2
CF saline–sham H3K4me3 Rep1
CF saline–sham H3K4me3 Rep2
CM malonate–MI H3K27me3 Rep1
CM malonate–MI H3K27me3 Rep2
CM malonate–MI H3K4me3 Rep1
CM malonate–MI H3K4me3 Rep2
CM malonate–sham H3K27me3 Rep1
CM malonate–sham H3K27me3 Rep2
CM malonate–sham H3K4me3 Rep1
CM malonate–sham H3K4me3 Rep2
CM saline–MI H3K27me3 Rep1
CM saline–MI H3K27me3 Rep2
CM saline–MI H3K4me3 Rep1
CM saline–MI H3K4me3 Rep2
CM saline–sham H3K27me3 Rep1
CM saline–sham H3K27me3 Rep2
CM saline–sham H3K4me3 Rep1
CM saline–sham H3K4me3 Rep2
0%
25%
50%
75%
100%
Proportion of Peaks
Genomic Feature
Promoter
5' UTR
Exon
Intron
3' UTR
Downstream
Distal Intergenic

Supplementary Figure 5. Correlation and peak distribution of CUT&RUN replicates.
a, Correlation heatmap of all samples showing clustering of samples by assessed histone marks and by sample condition. **b**, Peak distribution across all samples for both histone marks.

Supplementary Figure 3_Unprocessed western blot

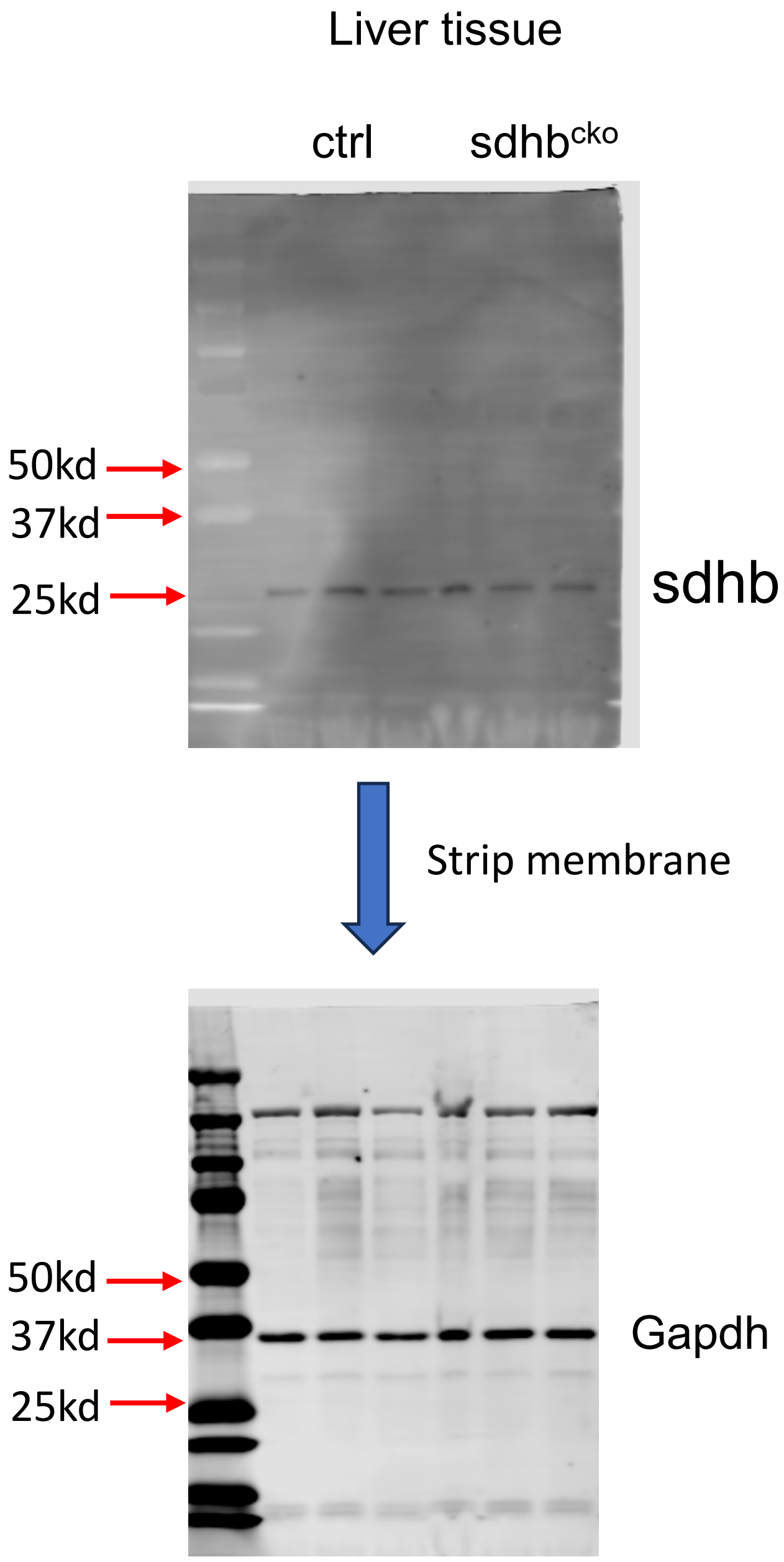

Supplementary Figure 3_statistical source data

Ctrl	$Sdhb^{cko}$
1	2.61260691
1.3096748	2.18351729
3.64049694	1.88644873
