# Supplementary material for: A metabolic–epigenetic switch governs multicellular cardiac repair following succinate dehydrogenase inhibition
Source: Nat Cardiovasc Res. 2026 Sep 28;5(10):1034–54. doi: 10.1038/s44161-026-00881-9 (PMC13658034; doi:10.1038/s44161-026-00881-9)
Supplement: Supplementary file 4 [file 44161_2026_881_MOESM2_ESM.pdf]

nature portfolio

Corresponding author(s): Ahmed I. Mahmoud

Last updated by author(s): Jun 25, 2025

Reporting Summary

Nature Portfolio wishes to improve the reproducibility of the work that we publish. This form provides structure for consistency and transparency in reporting. For further information on Nature Portfolio policies, see our Editorial Policies and the Editorial Policy Checklist.

Statistics

For all statistical analyses, confirm that the following items are present in the figure legend, table legend, main text, or Methods section.

n/a	Confirmed	
☐	☒	The exact sample size (*n*) for each experimental group/condition, given as a discrete number and unit of measurement
☐	☒	A statement on whether measurements were taken from distinct samples or whether the same sample was measured repeatedly
☐	☒	The statistical test(s) used AND whether they are one- or two-sided<br>*Only common tests should be described solely by name; describe more complex techniques in the Methods section.*
☐	☒	A description of all covariates tested
☐	☒	A description of any assumptions or corrections, such as tests of normality and adjustment for multiple comparisons
☐	☒	A full description of the statistical parameters including central tendency (e.g. means) or other basic estimates (e.g. regression coefficient) AND variation (e.g. standard deviation) or associated estimates of uncertainty (e.g. confidence intervals)
☐	☒	For null hypothesis testing, the test statistic (e.g. *F*, *t*, *r*) with confidence intervals, effect sizes, degrees of freedom and *P* value noted<br>*Give P values as exact values whenever suitable.*
☒	☐	For Bayesian analysis, information on the choice of priors and Markov chain Monte Carlo settings
☒	☐	For hierarchical and complex designs, identification of the appropriate level for tests and full reporting of outcomes
☒	☐	Estimates of effect sizes (e.g. Cohen's *d*, Pearson's *r*), indicating how they were calculated

Our web collection on statistics for biologists contains articles on many of the points above.

Software and code

Policy information about availability of computer code

Data collection	No software was used for data collection.
Data analysis	ImageJ was used for image analysis. GraphPad Prism was used for statistical analysis. The code used for bioinformatic analysis can be found at Zenodo (https://doi.org/10.5281/zenodo.21987923) and Github (https://github.com/duttall/2026-SDH-inhibition-snMultiome-CUTnRUN).

For manuscripts utilizing custom algorithms or software that are central to the research but not yet described in published literature, software must be made available to editors and reviewers. We strongly encourage code deposition in a community repository (e.g. GitHub). See the Nature Portfolio guidelines for submitting code & software for further information.

Data

Policy information about availability of data

All manuscripts must include a data availability statement. This statement should provide the following information, where applicable:

- Accession codes, unique identifiers, or web links for publicly available datasets
- A description of any restrictions on data availability
- For clinical datasets or third party data, please ensure that the statement adheres to our policy

Single nuclear RNA-seq and ATAC-seq data mice are available at the NCBI's Gene Expression Omnibus (GSE299884). All data generated and analyzed during this study are included in the manuscript and supplementary files.

Research involving human participants, their data, or biological material

Policy information about studies with human participants or human data. See also policy information about sex, gender (identity/presentation), and sexual orientation and race, ethnicity and racism.

Reporting on sex and gender	N/A
Reporting on race, ethnicity, or other socially relevant groupings	N/A
Population characteristics	N/A
Recruitment	N/A
Ethics oversight	N/A

Note that full information on the approval of the study protocol must also be provided in the manuscript.

Field-specific reporting

Please select the one below that is the best fit for your research. If you are not sure, read the appropriate sections before making your selection.

☒ Life sciences ☐ Behavioural & social sciences ☐ Ecological, evolutionary & environmental sciences

For a reference copy of the document with all sections, see nature.com/documents/nr-reporting-summary-flat.pdf

Life sciences study design

All studies must disclose on these points even when the disclosure is negative.

Sample size	No formal sample size calculation was performed. Sample sizes were determined based on available litter sizes to enable the use of littermate controls.
Data exclusions	No data was excluded.
Replication	All experiments were reproducible and successfully replicated. A minimum of three independent biological replicates was included for each experiment.
Randomization	Whenever possible, mice from the same litter were allocated to the same experimental group. Animals of both sexes were used, and experiments were conducted in a sex-blinded manner.
Blinding	Both data acquisition and analysis were blinded when possible. Blinding was implemented for experiments including imaging, echocardiography, and metabolomics. However, blinding was not possible for single-cell transcriptomic analyses.

Reporting for specific materials, systems and methods

We require information from authors about some types of materials, experimental systems and methods used in many studies. Here, indicate whether each material, system or method listed is relevant to your study. If you are not sure if a list item applies to your research, read the appropriate section before selecting a response.

Materials & experimental systems

n/a	Involved in the study	
☐	☒	Antibodies
☒	☐	Eukaryotic cell lines
☒	☐	Palaeontology and archaeology
☐	☒	Animals and other organisms
☒	☐	Clinical data
☒	☐	Dual use research of concern
☒	☐	Plants

Methods

n/a	Involved in the study	
☒	☐	ChIP-seq
☐	☒	Flow cytometry
☒	☐	MRI-based neuroimaging

Antibodies

Antibodies used	Primary antibodies used include anti-PCM1 (Sigma, HPA023374, 1:200), anti-Ki67 (Abcam, ab279653, 1:200), anti-phospho histone

Antibodies used	H3 Ser10 (Invitrogen, MA5-15220, 1:200), anti-Ki67 (Abcam, ab15580, 1:200), anti-phospho histone H3 Ser10 (Millipore, 06-570, 1:200), anti-aurora B (Sigma A5102, 1:50), anti-cardiac troponin T (Abcam, ab8295, 1:200), anti-BrdU (Abcam, ab1893, 1:200), anti-Postn (Abcam, ab14041, 1:200), anti-PDGFRα (Cell Signaling Technology, 3174,1:500), anti-Col3a1 (Abcam, ab184993, 1:200), anti-aSMA (Invitrogen, 14-5698-82, 1:200), anti-Vimentin (ABclonal, a19607, 1:200), anti-Sdhb (Abcam, ab14714, 1:1000), anti-Gapdh (Proteintech, 10494-1-AP, 1:1000), anti-Col3a1 (Abcam, ab184993, 1:1000), anti-H3K4me1 (Abcam, ab8895, 1:1000), anti-H3K4me2 (Abcam, ab39141, 1:1000), anti-H3K4me3 (Abcam, ab8580, 1:1000), anti-H3K9me1 (Abcam, ab8896, 1:1000), anti-H3K9me3 (Abcam, ab8898, 1:1000), anti-H3K27me3 (Abcam, ab192985, 1:1000), anti-H3K36me3 (Abcam, ab9050, 1:1000), anti-H3K79me3 (Abcam, ab2621, 1:1000), anti-H4K20me3 (Abcam, ab9053, 1:1000), anti-H3K9ace (Sigma-Aldrich, 06-942, 1:1000), anti-H3K14ace (Sigma-Aldrich, 07-353, 1:1000), anti-H3K27ace (Abcam, ab4729, 1:1000), anti-H3ace (Sigma-Aldrich, 06-599, 1:1000) and anti-H3 (Abcam, ab1791, 1:1000). Cell membrane was performed with WGA conjugated to Alexa Fluor 488 (50 mg/mL, Life Technologies, 1853992). Nuclear stain was performed with DAPI (ThermoFisher 62248)
Validation	Primary anitbodies were validated by either vendor, prior publications, or our lab using positive and negative controls.

Animals and other research organisms

Policy information about studies involving animals; ARRIVE guidelines recommended for reporting animal research, and Sex and Gender in Research

Laboratory animals	Animals were housed in ventilated cages under a 12-hour light/dark cycle with ad libitum access to food and water. Sdhb floxed (Sdhbfl/fl) mice were provided by Judith Favier at INSERM. CD-1 wild type mice were purchased from Charles River Laboratories. αMHC-MerCreMer mice (stock number 005657) and Periostin (Postn)-MerCreMer mice (stock number 029645) were obtained from the Jackson Laboratory.
Wild animals	This study did not involve wild animals.
Reporting on sex	For the single nucleus sequencing experiments, 8-week-old adult male mice were used. For all other experiments, both male and female mice were used, with littermates used as controls in all experiments.
Field-collected samples	This study did not involve field-collected samples.
Ethics oversight	Mouse studies were performed in accordance with the National Institutes of Health Guide for the Care and Use of Laboratory Animals and the Institutional Animal Care and Use Committee of Sanford Burnham Prebys and the University of Wisconsin-Madison.

Note that full information on the approval of the study protocol must also be provided in the manuscript.

Plants

Seed stocks	N/A
Novel plant genotypes	N/A
Authentication	N/A

Flow Cytometry

Plots

Confirm that:

- [x] The axis labels state the marker and fluorochrome used (e.g. CD4-FITC).
- [x] The axis scales are clearly visible. Include numbers along axes only for bottom left plot of group (a 'group' is an analysis of identical markers).
- [ ] All plots are contour plots with outliers or pseudocolor plots.
- [x] A numerical value for number of cells or percentage (with statistics) is provided.

Methodology

Sample preparation	Nonmyocytes for immunohistochemistry, Western blot, and CUT&RUN analysis were isolated from hearts. After euthanasia by cervical dislocation, the hearts would be perfused by cutting the right atria and inserting a 27g needle connected pump into the apex of left ventricle. Cold perfusion buffer (1x Hanks' Balanced Salt Solution (HBSS), 0.8 mM CaCl2) would then be pumped into the heart at a rate of 5 ml/min for 2 minutes. The ventricles would then be collected in a 6-well dish on ice and minced into 1 mm cubes. The tissue would then be transferred to a 50ml tube containing 10 ml per heart of collagenase II digestion solution (Worthington Laboratories, 500 units/ml in HBSS) and placed on a rotator in a 37 ℃ incubator for 30

minutes and manually agitated every 5-10 minutes. The solution would then be gently triturated using a 10 ml serological pipette and then centrifuged at 300g for 10 minutes. The supernatant was then aspirated and the pellet resuspended/ triturated with a 5 mL serological pipette in 5 ml/heart of a Collagenase/Dispase solution (Roche Diagnostics, 1 mg/ml in HBSS), transferred to a 15 ml tube and placed back in the 37 ℃ incubator for 20 minutes and manually agitated every 5-10 minutes. The digestion was terminated by adding 5 ml/heart of cold 2% FBS/HBSS. The cells were then centrifuged at 30g for 2 minutes to pellet myocytes and the supernatant containing the nonmyocytes was transferred to a new 15ml tube and filtered through a 40µm filter to further remove any potential myocytes in the supernatant.

Isolated non myocytes were stained with 1:1000 Ghost Dye Violet 450 (Cytek, Cat#13-0863-T100) to discriminate between viable and dead cells for 15 minutes in 2% FBS/HBSS and then centrifuged (400g for 10 minutes). After which, the supernatant was removed and replaced for antibody staining 1:50 Mefsk4-PE (Miltenyi Biotech; Cat# 130-120-166) and 1:20 ITGA7-Alexa Fluor 700 (Thermo Fisher Scientific; Cat# MA5-23607) in 2%FBS/HBSS for 30 minutes. All panels were validated using no-stain and single-stain controls. After staining, the cells were centrifuged, supernatant removed, washed again in 2% FBS/HBSS. The centrifugation and supernatant removal was repeated two more times before final suspension in 2 % FBS/ HBSS with 2mM EDTA.

Instrument

BD FACSAria

Software

BD FACSDiva 8.0.1

Cell population abundance

The amount of Mef-sk4 high and ITGA7- fibroblasts sorted depended largely on treatment conditions with an average proportion of 5-8% of total cells (approximately 200-300k fibroblasts per ventricle). Purity of the cells was assessed by an additional FACS sorting of the post-sorted cells. We observed on average a 95% purity from the live population of these same Mef-sk4 high and ITGA7- fibroblasts. Their identity was further confirmed through Pdgfr staining.

Gating strategy

Cell detection was performed using forward and side scatter (FSC and SSC). Singlets were identified using FSC-H and FSC-A scatter, and then again by SSC-H and SSC-A. Live cells were then gated by filtering out Ghost Dye Violet 450 + cells. Fibroblasts were then gated as high Mef-sk4 PE + and ITGA7 Alexa700 – (mural cell marker).

☒ Tick this box to confirm that a figure exemplifying the gating strategy is provided in the Supplementary Information.
