# Supplementary material for: A metabolic–epigenetic switch governs multicellular cardiac repair following succinate dehydrogenase inhibition
Source: Nat Cardiovasc Res. 2026 Sep 28;5(10):1034–54. doi: 10.1038/s44161-026-00881-9 (PMC13658034; doi:10.1038/s44161-026-00881-9)
Supplement: Supplementary file 5 [file 44161_2026_881_MOESM5_ESM.pdf]

Figure 4. B

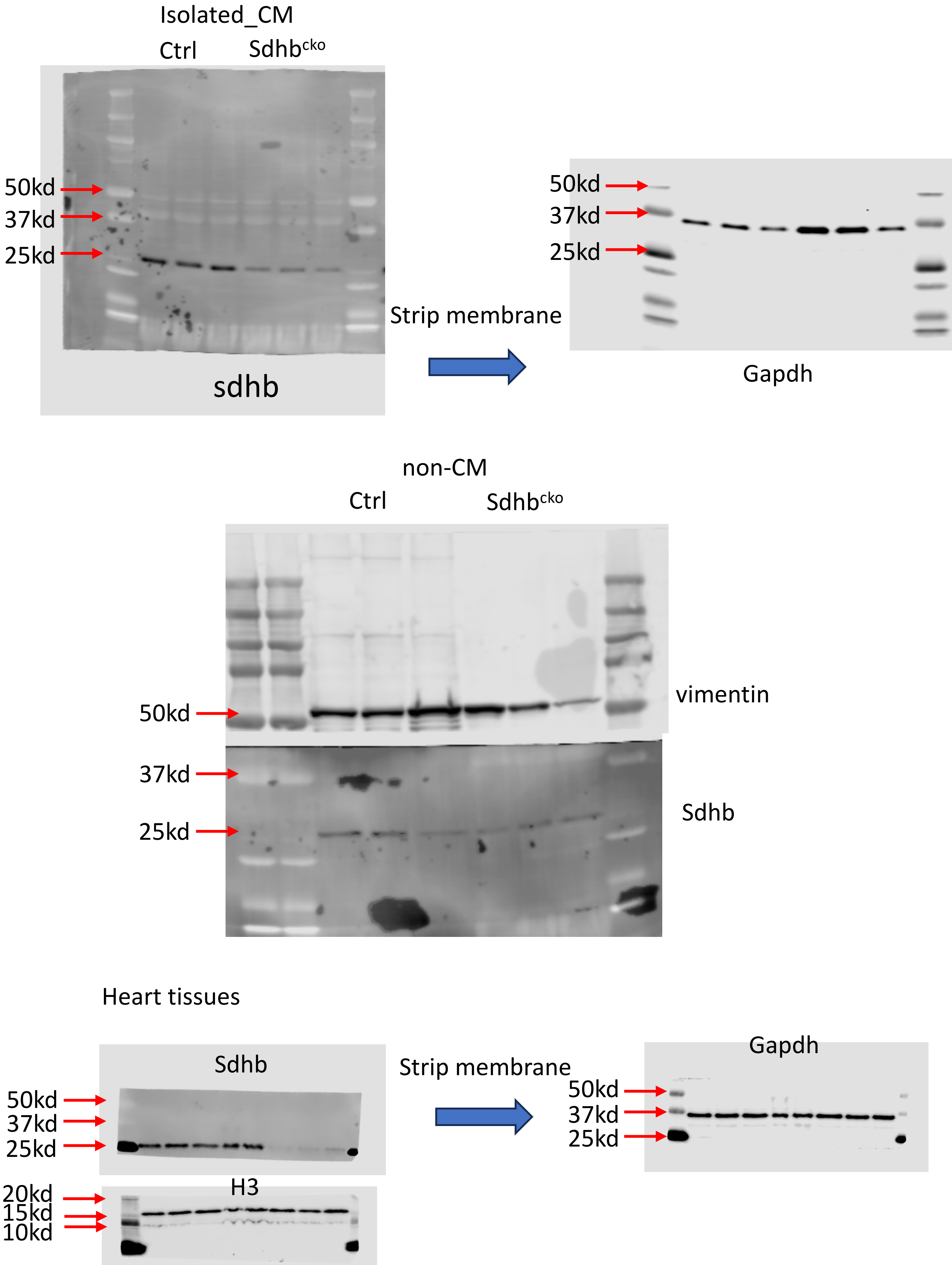

Isolated_CM
Ctrl
Sdhb^{cko}
50kd
37kd
25kd
sdhb
Strip membrane
50kd
37kd
25kd
Gapdh
non-CM
Ctrl
Sdhb^{cko}
50kd
vimentin
37kd
25kd
Sdhb
Heart tissues
Sdhb
50kd
37kd
25kd
H3
20kd
15kd
10kd
Strip membrane
Gapdh
50kd
37kd
25kd
