# Supplementary material for: A metabolic–epigenetic switch governs multicellular cardiac repair following succinate dehydrogenase inhibition
Source: Nat Cardiovasc Res. 2026 Sep 28;5(10):1034–54. doi: 10.1038/s44161-026-00881-9 (PMC13658034; doi:10.1038/s44161-026-00881-9)
Supplement: Supplementary file 6 [file 44161_2026_881_MOESM7_ESM.pdf]

Figure 5. B

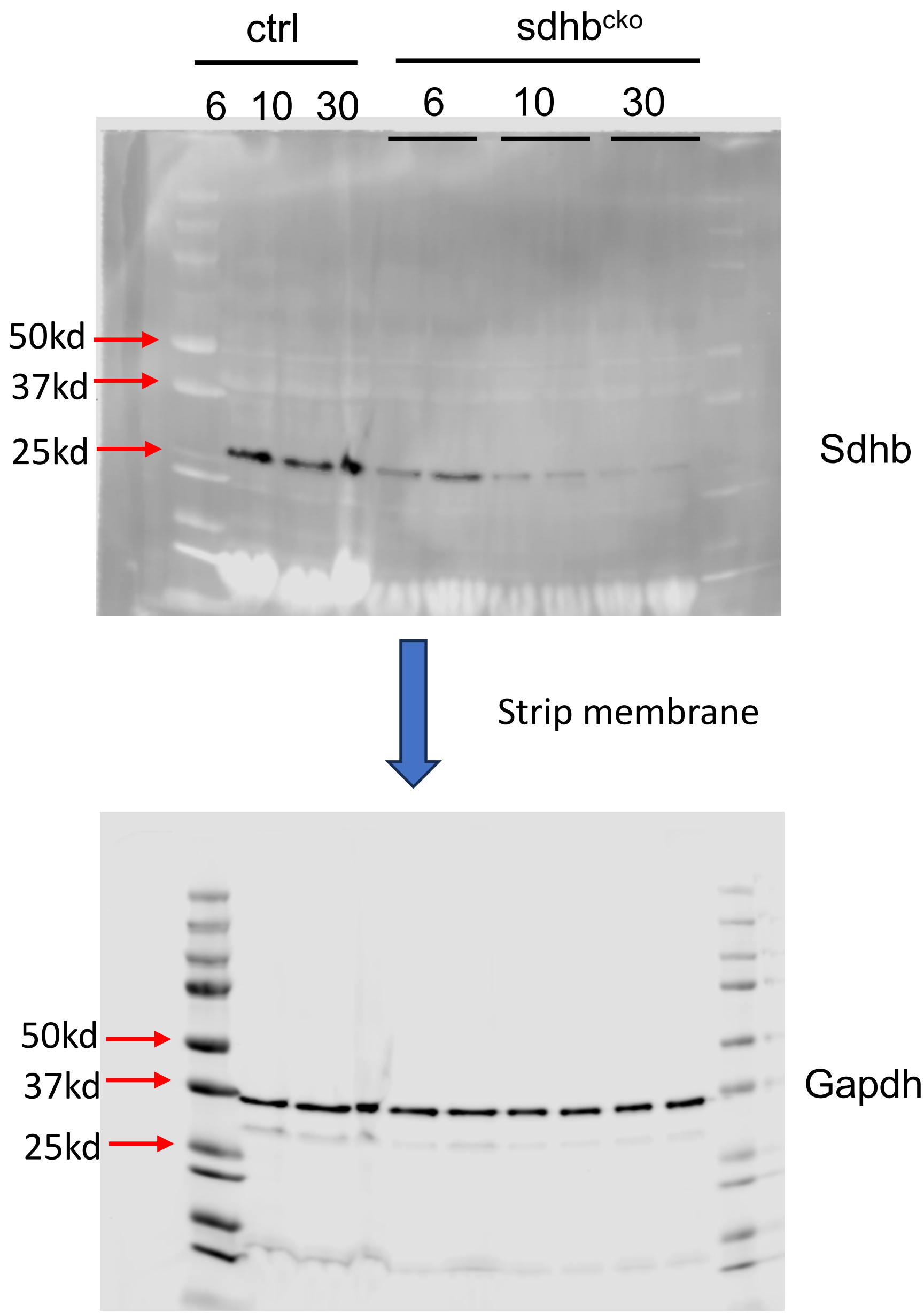

ctrl
sdhb^cko
6 10 30
6
10
30
50kd
37kd
25kd
Sdhb
Strip membrane
50kd
37kd
25kd
Gapdh
