# Supplementary material for: A metabolic–epigenetic switch governs multicellular cardiac repair following succinate dehydrogenase inhibition
Source: Nat Cardiovasc Res. 2026 Sep 28;5(10):1034–54. doi: 10.1038/s44161-026-00881-9 (PMC13658034; doi:10.1038/s44161-026-00881-9)
Supplement: Supplementary file 7 [file 44161_2026_881_MOESM9_ESM.pdf]

Figure 6. J

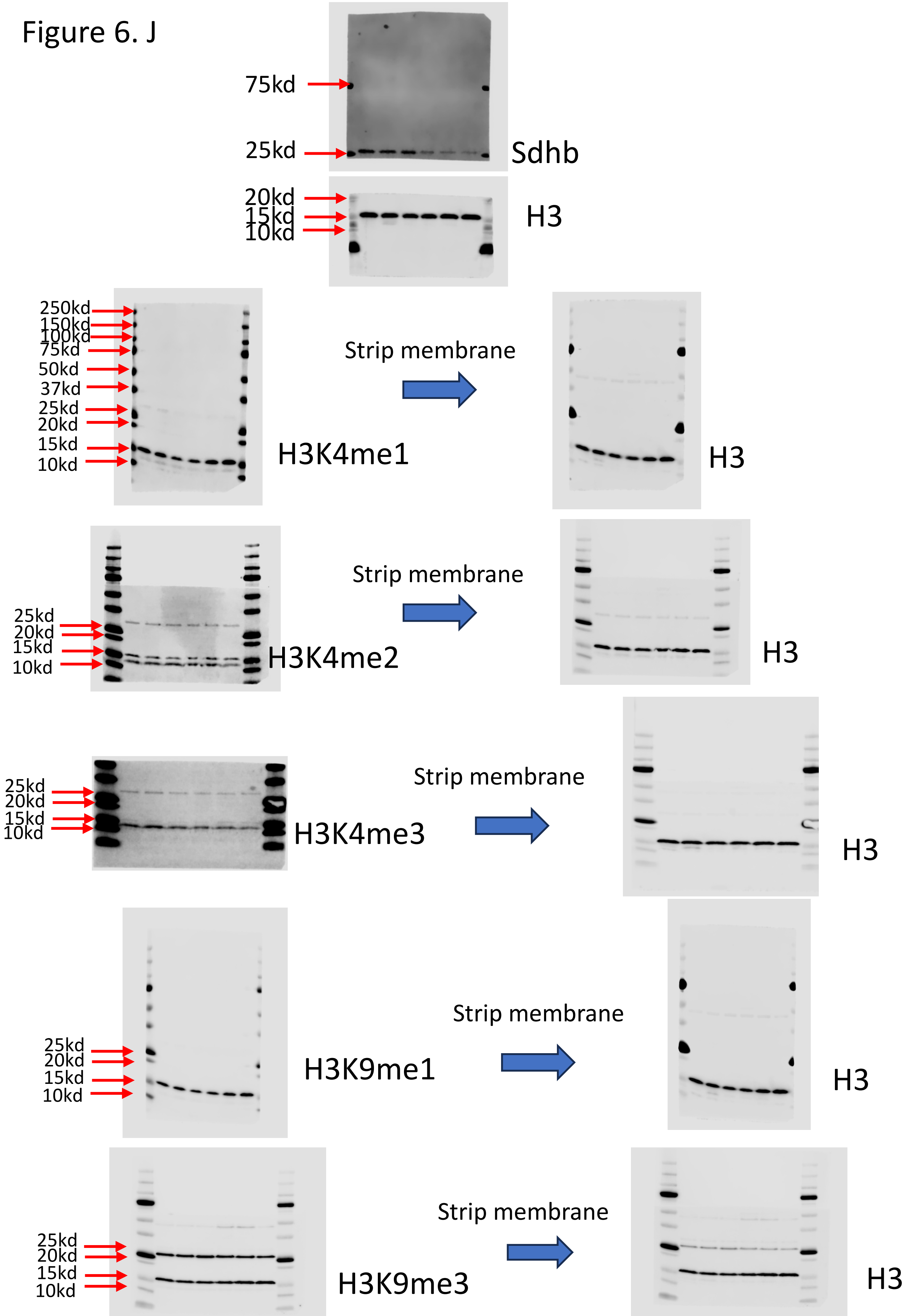

75kd
25kd
Sdhb
20kd
15kd
10kd
H3
250kd
150kd
100kd
75kd
50kd
37kd
25kd
20kd
15kd
10kd
H3K4me1
Strip membrane
H3
25kd
20kd
15kd
10kd
H3K4me2
Strip membrane
H3
25kd
20kd
15kd
10kd
H3K4me3
Strip membrane
H3
25kd
20kd
15kd
10kd
H3K9me1
Strip membrane
H3
25kd
20kd
15kd
10kd
H3K9me3
Strip membrane
H3

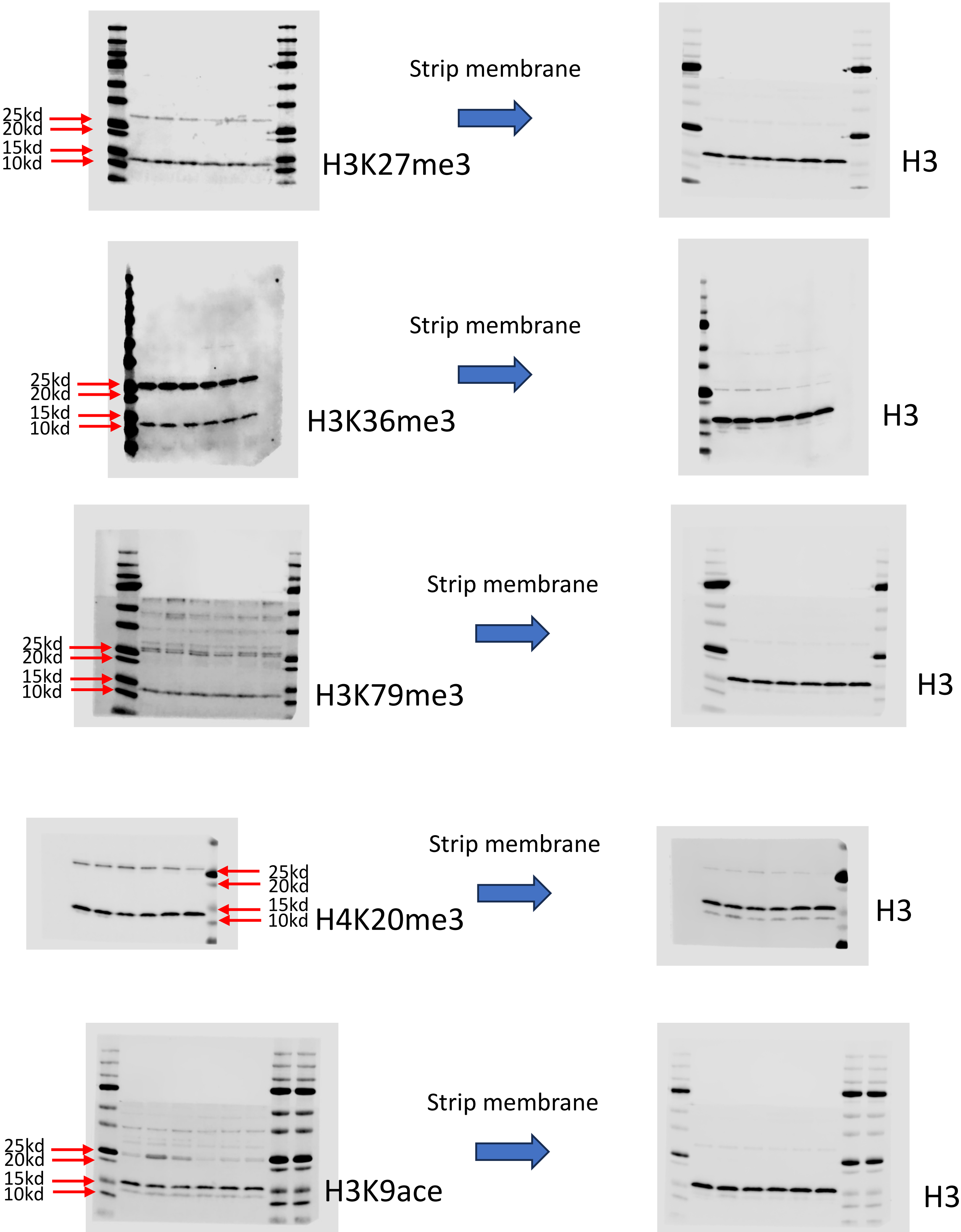
25kd
20kd
15kd
10kd
H3K27me3
Strip membrane
H3
25kd
20kd
15kd
10kd
H3K36me3
Strip membrane
H3
25kd
20kd
15kd
10kd
H3K79me3
Strip membrane
H3
25kd
20kd
15kd
10kd
H4K20me3
Strip membrane
H3
25kd
20kd
15kd
10kd
H3K9ace
Strip membrane
H3

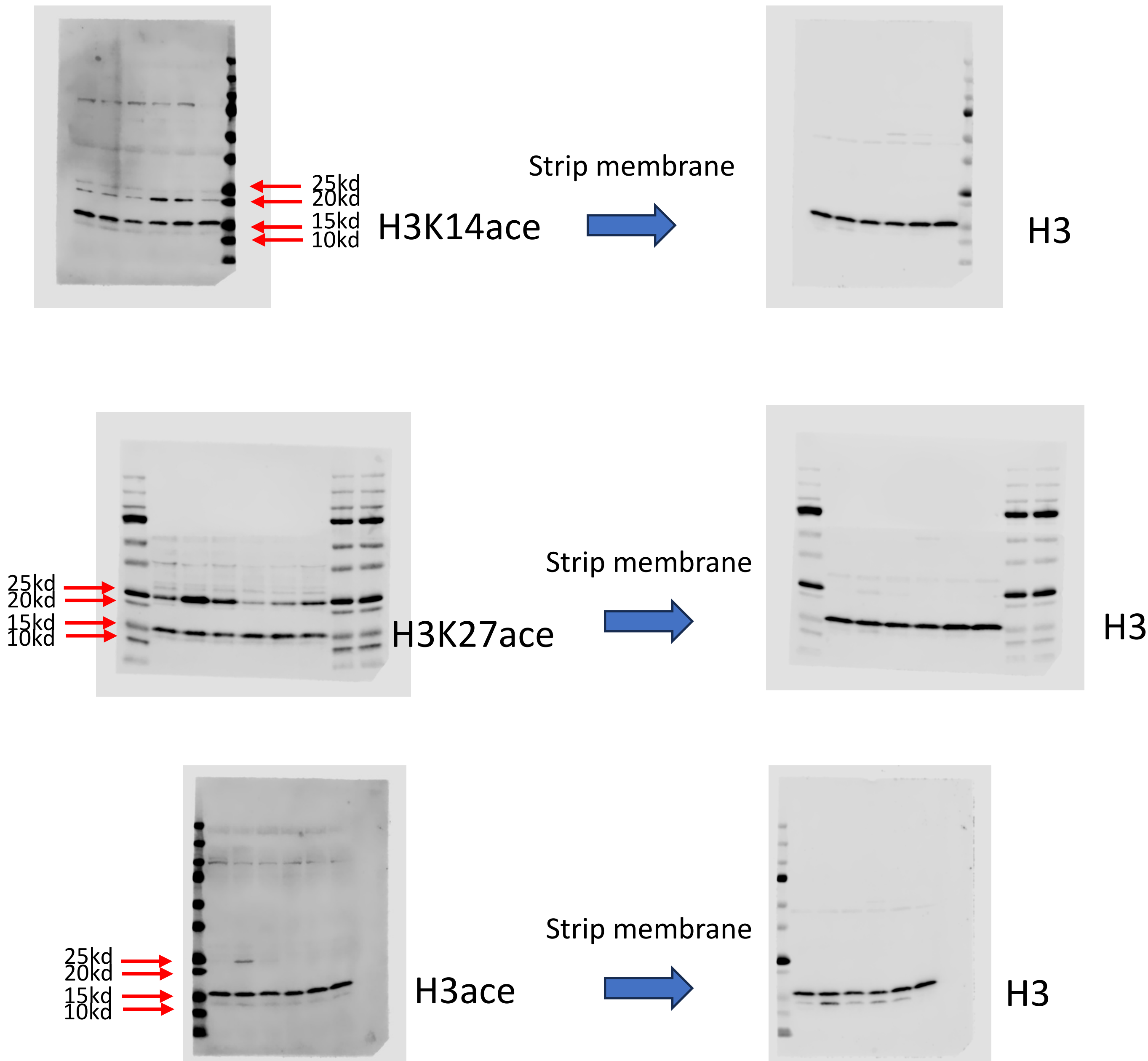
25kd
20kd
15kd
10kd
H3K14ace
Strip membrane
H3
25kd
20kd
15kd
10kd
H3K27ace
Strip membrane
H3
25kd
20kd
15kd
10kd
H3ace
Strip membrane
H3
